# Gadolinium-based contrast agent accelerates the migration of astrocyte via integrin αvβ3 signaling pathway

**DOI:** 10.1038/s41598-022-09882-7

**Published:** 2022-04-07

**Authors:** Winda Ariyani, Wataru Miyazaki, Yoshito Tsushima, Noriyuki Koibuchi

**Affiliations:** 1 Research Fellow of Japan Society for the Promotion of Science, 5-3-1 Kojimachi, Chiyoda-ku, Tokyo 102-0083 Japan; 2 Department of Integrative Physiology, Gunma University Graduate School of Medicine, 3-39-22 Showa-machi, Maebashi, Gunma 371-8511 Japan; 3 Department of Bioscience and Laboratory Medicine, Hirosaki University Graduate School of Health Science, 66-1 Hon-cho, Hirosaki, Aomori 036-8564 Japan; 4 Department of Diagnostic Radiology and Nuclear Medicine, Gunma University Graduate School of Medicine, 3-39-22 Showa-machi, Maebashi, Gunma 371-8511 Japan

**Keywords:** Neuroscience, Cell adhesion, Cell migration, Cell signalling, Mechanism of action, Metals

## Abstract

Gadolinium (Gd)-based contrast agents (GBCAs) are chemicals injected intravenously during magnetic resonance imaging to enhance the diagnostic yield. Repeated use of GBCAs causes their deposition in the brain. Such deposition may affect various neuronal cells, including astrocytes. In this study, we examined the effect of GBCAs (Omniscan, Magnescope, Magnevist, and Gadovist) on astrocyte migration, which is critical for formation of neurons during development and maintaining brain homeostasis. All GBCAs increased cell migration and adhesion with increased actin remodelling. Knockdown of integrin αvβ3 by RNAi or exposure to integrin αvβ3 inhibitor reduced astrocyte migration. GBCAs increased phosphorylation of downstream factors of αvβ3, such as FAK, ERK1/2, and Akt. The phosphorylation of all these factors were reduced by RNAi or integrin αvβ3 inhibitor. GBCAs also increased the phosphorylation of their downstream factor, Rac1/cdc42, belonging to the RhoGTPases family. Coexposure to the selective RhoGTPases inhibitors, decreased the effects of GBCAs on cell migration. These findings indicate that GBCAs exert their action via integrin αvβ3 to activate the signaling pathway, resulting in increased astrocyte migration. Thus, the findings of the study suggest that it is important to avoid the repeated use of GBCAs to prevent adverse side effects in the brain, particularly during development.

## Introduction

Astrocytes are the most abundant cell type in the central nervous system (CNS) and cover nearly half of the adult mammalian brain volume^1–3^. Their role in brain development, maintenance of homeostasis, and as defence mechanism following brain injury have been well documented^4–6^. In the early neurogenic phase, astrocytes are formed from either the embryonic radial glia in the ventricular zone (VZ) or progenitor cells in the subventricular zone (SVZ)^2,7,8^. Immature astrocytes migrate to the cortical layers where they undergo additional round proliferation before exiting the cell cycle for maturation^9,10^. During brain development, astrocytes play an important role in controlling neurogenesis (neuronal development), neuronal migration, axonal growth, synapse formation and function, maintenance of the blood–brain barrier, and controlling homeostasis^2,4,8^. Thus, astrocyte migration from the VZ or SVZ is the critical first step in the regulation of brain development by this cell type.

Cell migration is determined by the polarization, coordinated regulation of actin cytoskeletal elements, and adhesive structures^9,11^. These processes require changes in cell–cell and/or cell-extracellular matrix (ECM) adhesion properties in which cell adhesion molecules are involved in binding with other cells or ECMs^9,12^. Integrins are a group of heterodimeric transmembrane proteins and they play a vital role during astrocyte proliferation and migration^9,12^. The activation of integrins leads to activation of the downstream intracellular signaling pathways through focal adhesion kinase (FAK), Src, Rac, and Rho, which jointly define the size, duration, and strength of adhesion^13^.

Among integrins, integrin αvβ3 plays an important role during cell migration and is highly expressed in astrocytes^14,15^. Integrin αvβ3 is upregulated in astrocytes during brain development and following brain injury. Binding of the integrin αvβ3 with their ligands activates FAK that functions as a receptor-proximal regulator of cell motility^16^. FAK activity and downstream signaling can promote changes in actin and microtubule structures. FAK can influence the activity of RhoGTPases', such as Cdc42, Rac, and RhoA, via direct interaction with, or phosphorylation of protein activators or inhibitors of RhoGTPases^16,17^. Activated integrin αvβ3 induces the cell to polarize, extend filopodia and lamellipodia to the leading front, and form focal adhesions, which consist of a layer made up of bundles of actin microfilaments called stress fibers^12,15^. These changes in turn activate astrocyte adhesion with other cells, facilitating their spreading, and migration. Thus, it is reasonable to speculate that disturbances in the integrin αvβ3 signaling pathway could impair astrocyte migration.

Gadolinium-based contrast agents (GBCA) are intravenous drugs used in magnetic resonance imaging (MRI) to enhance the diagnostic yield. In the free form, Gadolinium ion (Gd^3+^) is extremely toxic. Therefore, it is chelated with an organic ligand molecule to produce highly soluble non-toxic complexes^18^. GBCAs are classified as linear or macrocyclic, based on their chemical compositions, and ionic or non-ionic based on their ion charge^19^. Following administration, GBCA is deposited in several organs, including the brain^20^. This deposition may affect not only neurons but also astrocytes^21–23^.

Although administration of GBCA during pregnancy or lactation is not recommended, it is often used for pregnant women if the imaging is essential^24^. GBCAs pass through the placental barrier and enter the fetal circulation^25^. Thus, GBCA may accumulate in fetal brain and cause adverse effect. Using animal model, we have previously shown such a possibility^26^. GBCA is also often administered during recovery after brain injury such as ischemic stroke^27^, when astrocytes migrate actively for brain repair. Thus, accumulated GBCA may affect the astrocyte function such as migration, atlhough the mechanisms of GBCA action on astrocyte function has not yet been fully classified.

Both Gd^3+^ and GBCA have been found to bind to and modulate integrin αvβ3^28–32^. Free Gd^3+^ has been found to increase the phosphorylation of FAK, extracellular regulated kinase 1/2 (ERK1/2), and Akt, a direct target for integrin αvβ3, leading to increased cell proliferation and enhanced focal adhesion^28,29^, indicating that Gd^3+^ may be at least in part dissociated from GBCA and induced such effect. However, Gd depositions in the brain consists of both soluble and insoluble forms^33,34^. The insoluble form may be Gd^3+^ that was released from GBCA and bound with organic or inorganic anions, whereas soluble forms consist of Gd-binding macromolecules and low molecular weight Gd complexes that might be the intact GBCAs. Based on these findings, we considered it important to examine the effect of GBCA on the integrin αvβ3 signaling pathway, which is responsible for astrocyte migration.

**Figure 1:**
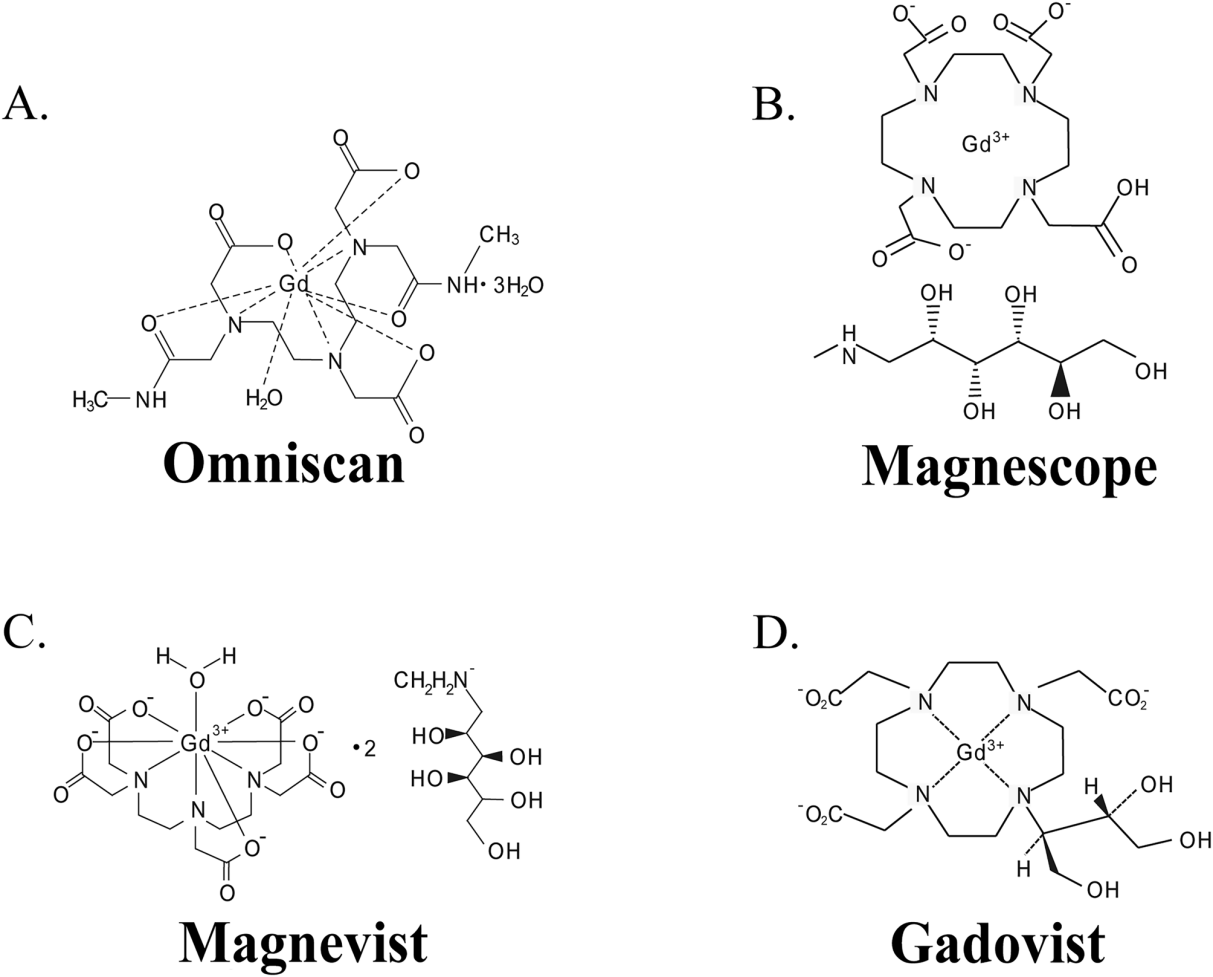
Chemicals structures of GBCAs.

The final objectives of our study is to clarify the mechanisms of various GBCAs toxicity on brain development and function. The clarification may help to select appropriate GBCA and its exposure protocol to avoid adverse effect by repeated exposure during brain development and/or regeneration. For such a purpose, we examined the effects of the four types of GBCA (Omniscan, Magnescope, Magnevist, and Gadovist) (Fig. 1) in migrating astrocytes, using astrocyte-enriched cultures of the cerebral cortex for focal adhesion and cell invasion assays, rat astrocyte-derived C6 clonal cells for wound healing assays and human astrocytoma-derived U87MG clonal cells for Western blot analysis. The reason we used clonal cells in addition to primary cultured astrocytes was because wound healing assays cannot be performed with primary cultured astrocytes since migration speed is too slow, and most antibodies for integrin downstream proteins are generated against human proteins. It was indeed confirmed that the basic nature and response to GBCAs are essentially the same among three subsets of cells used in the present study (See Supplementary Fig. S1). Our findings revealed that GBCAs caused focal adhesion and F-actin rearrangement leading to accelerated cell migration mediated by integrin αvβ3, at least in part. These effects were reduced by short interfering RNA (siRNA) knockdown of integrin αvβ3 and coexposure with cRGDfV, cyclic RGD peptides that are selective antagonists to integrin αvβ3 and αvβ5. Furthermore, activation of integrin αvβ3 by GBCAs activated the FAK/PI3K/ERK signaling pathway that induces RhoGTPases to accelerate astrocyte cell migration.

## Results

### GBCAs-accelerated astrocytes migration via integrin αvβ3

Interaction of astrocytes with its surrounding structures is crucial for physiological tissue organization and performing its functions during development and pathological conditions during brain injury^2,8^. In order for astrocytes to move and migrate through a complex three-dimensional (3D) environment, not only do their chemical and physical properties play an important role, but these cells must also quickly adapt in the short term to the physical properties of their surroundings. The 2D (two-dimensional) migration with flat plastic and 3D migration under the physical constraints of the surrounding cell and ECM has been used to elucidate the detailed mechanism of chemical and physical properties during astrocyte migration in cultured cells^35^. Therefore, we examined the effect of GBCAs in 2D and 3D cell migration using wound healing and cell invasion assays, respectively.

Before we conducted this study, we have examined the cytotoxic effect of both gadolinium and GBCAs by MTS cell proliferation assay (range 0.1 nM–100 µM). We found that GBCAs exposure starting 10 µM or gadolinium exposure starting 100 nM reduced the cell viability of astrocytes (Supplementary Fig. S6). Furthermore, the doposition of GBCAs in the brain ranges from 0.1 to 10 nM^36–38^ and the concentration of GBCA that was found in the waterwaste and sea water ranges from 0.1 to 100 nM^39,40^. Therefore, we used the doses ranging from 1 to 100 nM in the present study.

**Figure 2:**
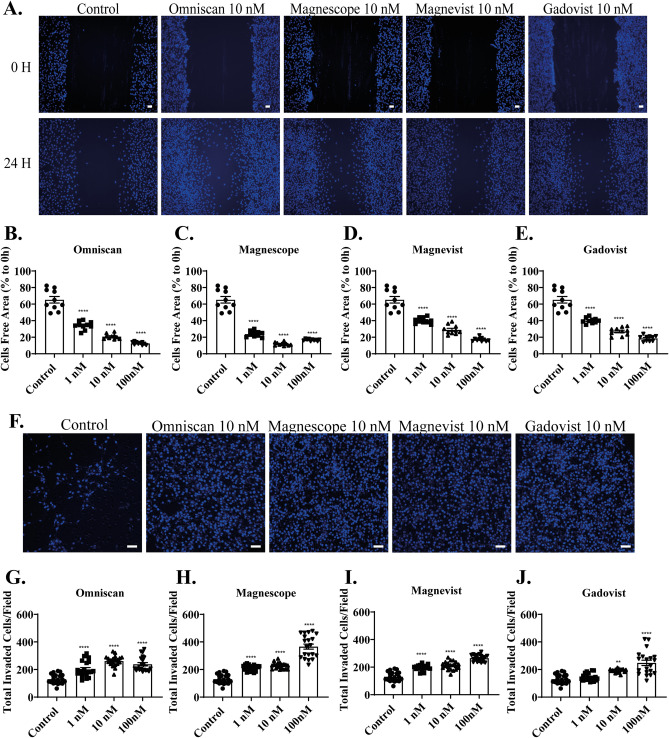
GBCAs accelerated astrocyte migration. (**A**) Representative photomicrographs showing the effects of GBCAs on 2D wound healing assays using C6 cells. Live-cell staining was performed using Cellstain-Hoechst 33,258 (Dojindo Molecular Technologies, Inc., Japan). Quantitative analysis of the effect of Omniscan (**B**), Magnescope (**C**), Magnevist (**D**), and Gadovist (**E**) (1–100 nM) on cell migration measured by wound healing assay. (**F**) Representative photomicrographs showing the effects of GBCAs on 3D matrigel invasion assays using astrocytes. Cell nuclei were stained with DAPI. Quantitative analysis of the effect of Omniscan (**G**), Magnescope (**H**), Magnevist (**I**), and Gadovist (**J**) (1–100 nM) on cell invasion was performed by matrigel invasion assay. The total number of cells was quantified using ImageJ software (NIH). Bars represent 50 μm. Data are expressed as the mean ± SEM of at least three independent experiments. ****p < 0.0001, **p < 0.01, indicates statistical significance were analysed by ANOVA continued with post hoc Bonferroni’s or Turkey test compared with the control.

In the wound healing assay, the representative photomicrograph images of cells stained using live-cell Hoechst staining after exposure with 10 nM GBCAs for 24 h are shown (Fig. 2A). GBCAs, except Magnescope, increased cell migration of C6 astrocyte clonal cells in a dose-dependent manner (Fig. 2B–E). Magnescope also significantly accelerated cell migration at all concentrations, although the magnitude decreased at 100 nM compared with control group (Fig. 2C). We also examined 3D astrocyte migration using invasion assays. Representative photomicrographs of invading astrocytes stained with 4′,6-diamidino-2-phenylindole (DAPI) after exposure to GBCAs are shown (Fig. 2F). Omniscan (Fig. 2G), Magnescope (Fig. 2H), Magnevist (Fig. 2I), and Gadovist (Fig. 2J) showed a significant acceleration of astrocyte migration in 3D migration without a clear dose-dependency. These results indicate that GBCAs can induce cell migration in C6 clonal cells and astrocytes.

**Figure 3:**
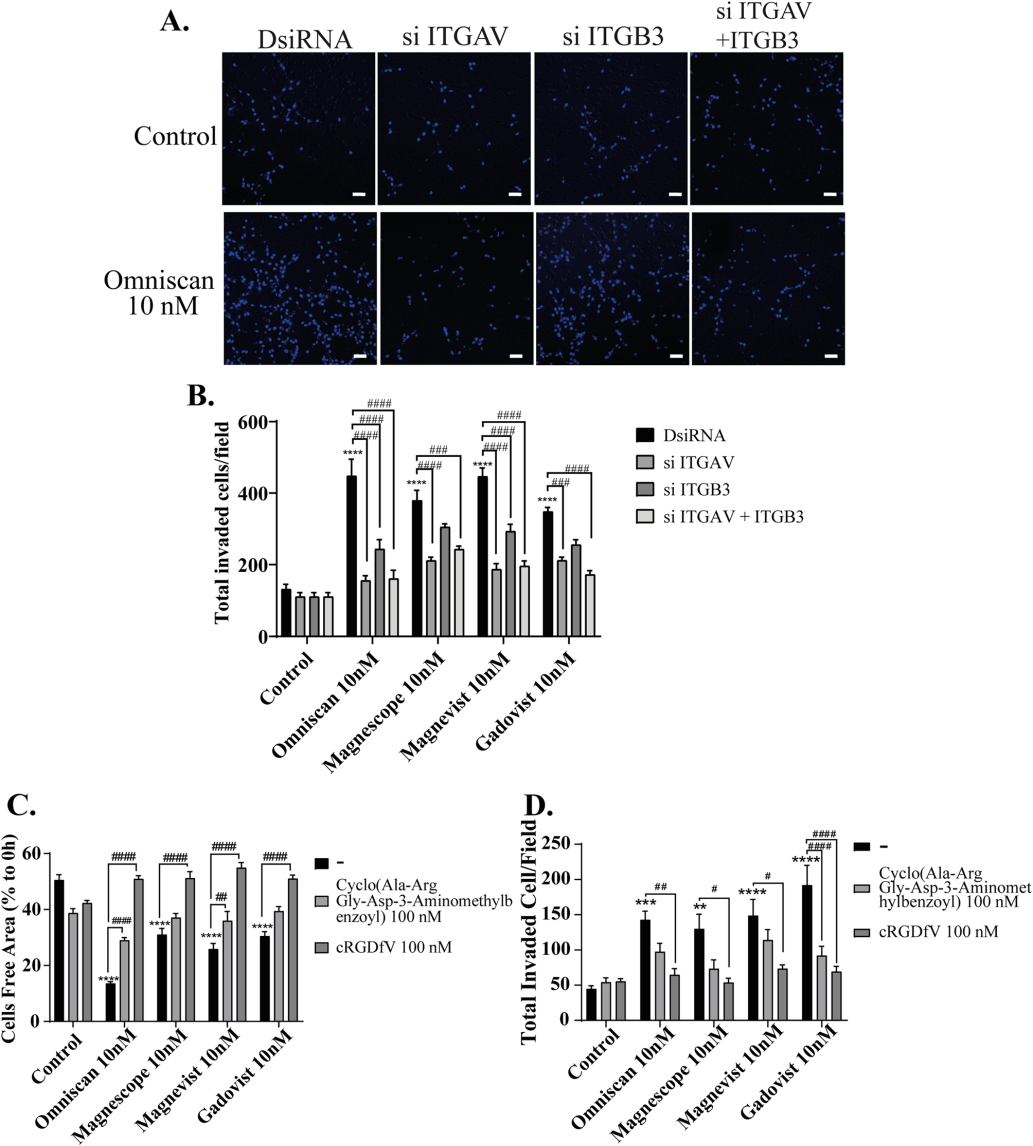
GBCAs increased astrocyte migration via integrin αvβ3. (**A**) Representative photomicrographs showing the effects of GBCAs after knockdown of ITGAV, ITGB3, or ITGAV and B3 on 3D matrigel invasion assays using astrocytes. Quantitative analysis of the effect of GBCAs after knockdown of ITGAV, ITGB3, or ITGAV and B3 on cell invasion (**B**) or coexposure with integrin inhibitor on wound healing assay (**C**) or cell invasion assays (**D**). The total number of cells was quantified using ImageJ software (NIH). Bars represent 50 μm. Data are expressed as the mean ± SEM of at least three independent experiments. ****p < 0.0001, ***p < 0.001, **p < 0.01, indicates statistical significance were analysed by two-way ANOVA continued with post hoc Bonferroni’s or Turkey test compared with the control. ^####^*p* < 0.0001, ^###^*p* < 0.001, ^##^*p* < 0.01, ^#^*p* < 0.05, indicates statistical significance were analysed by two-way ANOVA continued with post hoc Bonferroni’s or Turkey.

Our previous study showed that GBCAs disrupt the action of thyroid hormone (TH) receptors, leading to aberrant Purkinje cell development^22^. Integrin αvβ3 is known as TH binding site in the membrane, which has high possibility also to be disrupt by GBCAs. In addition, free Gd^3+^ itself is known to activate the integrin αvβ3–mediated signaling pathway^29^. Based on these findings, we considered it important to examine the effect of GBCA on the integrin αvβ3 signaling pathway, which is responsible for astrocyte migration. Therefore, to examine the involvement of integrin αvβ3 on GBCAs–induced astrocyte migration, we used siRNA against integrin αv or β3 subunit to knock–down their RNA expression in astrocytes. We also performed double knockdown of both integrin αv and β3 subunit. Representative photomicrographs of invading astrocytes stained with DAPI after GBCA exposure with knockdown of integrins are shown (Fig. 3A). DsiRNA, si ITGAV, or si ITGB3 istself did not significantly affect the astrocyte migration. Knockdown of integrin αv subunit reduced all cell migration induced by all four type of GBCAs, whereas knockdown integrin β3 subunit reduced only Omniscan- and Magnevist-induced cell migration (Fig. 3B). Furthermore, coexposure with 100 nM of cyclo (Ala-Arg-Gly-Asp-3-Aminomethylbenzoyl), cyclic RGD peptides as a specific antagonist to integrin αvβ3 or with 100 nM of cRGDfV (antagonist to integrin αvβ3 and αvβ5) reduced significantly in both Omniscan- or Magnevist-mediated cell migrations. On the other hand, Magnescope- and Gadovist-mediated cell migration was suppressed after coexposure with 100 nM of cRGDfV (Fig. 3C). Furthermore, in the 3D migration, GBCA-mediated cell migration was significantly suppressed by coexposure with 100 nM of cRGDfV (Fig. 3D). It should be noted that we have also used gadolinium chloride to examine the effect of Gd^3+^ on integrin αvβ3–mediated glial cell migration (Supplementary Fig. S7). Although Gd^3+^ activated the migration, integrin αvβ3 inhibitor did not alter the effect of Gd^3+^, indicating that Gd^3+^ affect the migration through different pathway. Because the objective of this study is to examine the effect on integrin αvβ3-mediated pathway, we did not used Gd^3+^ for further experiment. These results indicate that GBCAs-accelerated astrocytes migration was mediated via integrin αvβ3, at least in part. However, the involvement of other integrins, such as integrin αvβ5, cannot be excluded.

### GBCAs increased focal adhesion and F-actin rearrangement through the activation of integrin αvβ3

**Figure 4:**
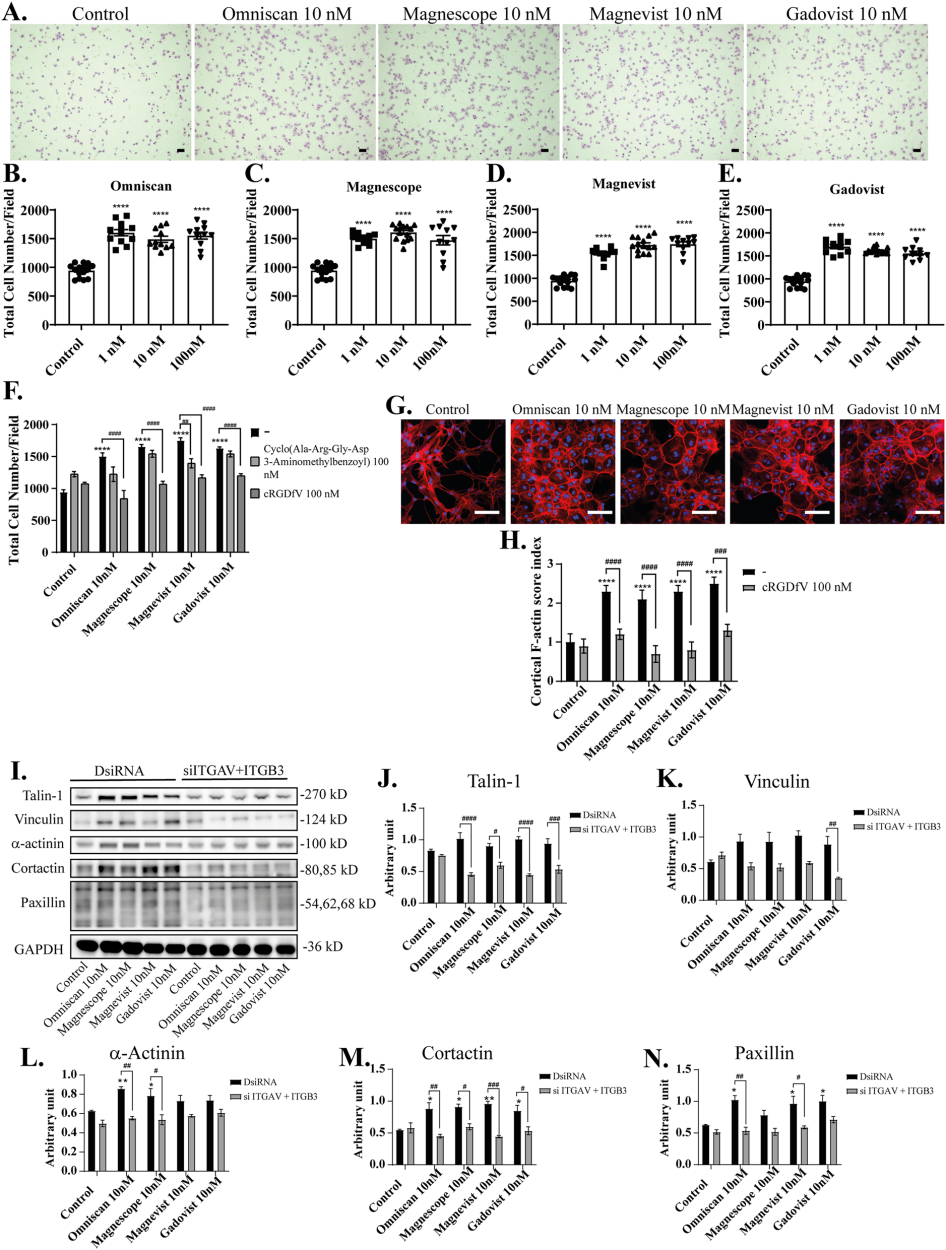
Effects of GBCAs on focal adhesion and F-actin formation in astrocytes. (**A**) Representative photomicrographs showing the effects of GBCAs on cell adhesion assays using astrocytes. Cells were stained with cresyl violet (Sigma). Quantitative analysis of the effect of Omniscan (**B**), Magnescope (**C**), Magnevist (**D**), and Gadovist (**E**) (1–100 nM) on cell adhesion. (**F**) Quantitative analysis of the effect of GBCAs after coexposure with integrin inhibitor on cell adhesion assays. Mouse primary cerebral cortex astrocytes were cultured for seven days, followed by fluorescence labeling analysis using phalloidin F-actin and DAPI. Astrocytes were exposed to GBCAs and/or cRGDfV for 60 min after serum-starvation for 6 h. (**G**) Representative photomicrographs showing the F-actin and DAPI staining to examine the effects of GBCA on cortical actin stress fibers formation. (**H**) Changes in the cortical F-actin score index after coexposure with cRGDfV. Bars represent 50 μm. (**I**) Representative blots of talin-1, vinculin, α-actinin, cortactin, paxillin, and GAPDH levels of 60 min GBCAs exposure after knockdown of ITGAV and ITGB3 in U87MG cells. Quantitative analysis of GBCAs effect on the protein expression levels of talin-1 (**J**), vinculin (**K**), α-actinin (**L**), cortactin (**M**), and paxillin (**N**). Protein band intensities (single band for talin-1, vinculin, α-actinin, and GAPDH; two bands for cortactin; three bands for paxillin) were quantified using Fiji ImageJ software (NIH). Bars represent 50 μm. Data are expressed as the mean ± SEM of at least three independent experiments. ****p < 0.0001, ***p < 0.001, **p < 0.01, *p < 0.05, indicates statistical significance were analysed by two-way ANOVA continued with post hoc Bonferroni’s or Turkey test compared with the control. ^####^*p* < 0.0001, ^###^*p* < 0.001, ^##^*p* < 0.01, ^#^*p* < 0.05, indicates statistical were analysed by two-way ANOVA continued with post hoc Bonferroni’s or Turkey.

Astrocytes expressed integrin αvβ3 that plays an essential role during cell migration and adhesion^41^. A focal adhesion complex is a structural unit composed of a high density of proteins involved in relaying the information flow from outside the cell to the inside. The extent of physical attachment depends on the nature of signaling the cell receives. Quantifying the amount of cell attachment should be proportional to the number of attached focal adhesion sites formed^42^. We thus examined focal adhesion of astrocytes by focal adhesion assay after exposure to GBCAs. The representative photomicrographs of attached astrocytes stained with cresyl violet are shown (Fig. 4A). GBCAs exposure with 1–100 nM concentrations increased astrocyte attachment without clear dose-dependency (Fig. 4B–E). These results indicate that GBCAs can induce focal adhesion of astrocytes.

Furthermore, coexposure with 100 nM cRGDfV significantly suppressed GBCAs induced focal adhesion of astrocytes (Fig. 4F), whereas no significant difference was observed after coexposure with 100 nM of cyclo (Ala-Arg-Gly-Asp-3-Aminomethylbenzoyl), except Magnevist. There are no significant effects of cRGDfV or cyclo (Ala-Arg-Gly-Asp-3-Aminomethylbenzoyl) in the focal adhesion of astrocytes. These results showed that GBCA-induced cell adhesion tends to reduce after co-exposure with 100 nM of cRGDfV (Fig. 4F). These results indicate that GBCAs increase focal adhesion of astrocytes through integrin. As for migration assays, not only integrin αvβ3 but other integrins may also be involved in the focal adhesion.

The ability of cells to attach and migrate requires complex molecular events initiated by the assembly of F-actin to alter the cellular morphology. The interaction of integrin αvβ3 and its ligands will induce the cell to polarize, extend filopodia and lamellipodia to the leading front, and form focal adhesions and bundles of actin microfilaments called stress fibers. Thus, we examined the reorganization of actin filaments using cortical F-actin score (CFS) index after visualization of F-actin with Phalloidin-iFluor 594 reagent. The CFS index was determined based on F-actin cytoskeletal reorganization for each cell^43,44^. It was scored on a scale ranging from 0 to 3, based on the degree of cortical F-actin ring formation 0, no cortical F-actin, normal stress fibers; 1, cortical F-actin deposits extending across less than half the cell border; 2, cortical F-actin deposits exceeding half the cell border; and 3, complete cortical ring formatting or total absence of central stress fibers or both. GBCAs attenuated formation of stress fibers to increase cortical actin filaments in astrocytes after 30-min of exposure (Fig. 4G). The CFS index significantly increased after 10 nM exposure of GBCAs, and these effects were reduced by coexposure with 100 nM of cRGDfV (Fig. 4H). We also examined the effects of GBCAs on focal adhesion proteins related to actin reorganization in U87MG cells. We found that GBCAs increased the levels proteins, namely, of talin-1, vinculin, α-actinin, cortactin, and paxillin, and these effects were reduced by knockdown of both integrin αv and β3 subunit also reduced the expression of focal adhesion protein, including talin-1, vinculin, α-actinin, cortactin, and paxillin (Fig. 4I–N). In addition, knock–down of integrin αvβ3 did not affect the basal levels of focal adhesion protein. These results indicate that the exposure to GBCAs induced adhesions composed of integrins, which then leads to F-actin rearrangement, accelerating cortical actin ring formation, which may result in accelerated astrocyte migration.

### GBCAs-activated FAK/ERK1/2/Akt signaling pathways via integrin αvβ3, leading to the astrocyte migration

**Figure 5:**
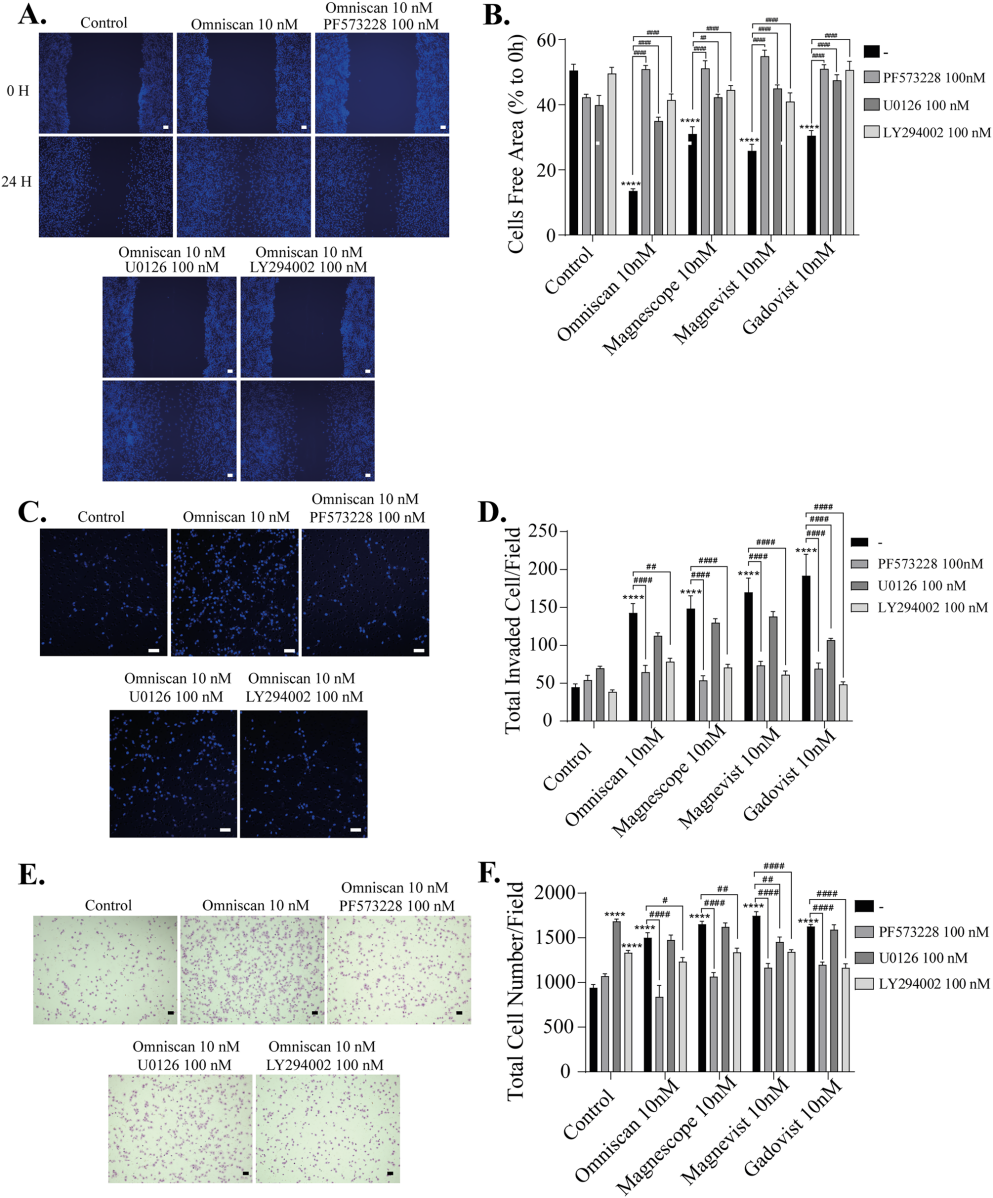
GBCAs enhanced the astrocytes migration via the activation of the FAK/PI3K/Akt/ERK1/2. (**A**) Representative photomicrographs showing the effects PF573228, a FAK inhibitor, U0126, an ERK1/2 inhibitor, and/or LY294002, a PI3K inhibitor, on Omniscan–accelerated C6 cell migration measured using a wound-healing assay. (**B**) Quantitative analysis of the effects of PF573228, U0126, or LY294002 on GBCAs-accelerated cell migration measured using wound healing assays using C6 cells. (**C**) Representative photomicrographs showing the effects PF573228, U0126, or LY294002 on Omniscan-accelerated astrocyte migration measured using matrigel invasion assays. (**D**) Quantitative analysis of the effects of PF573228, U0126, or LY294002 on GBCAs-accelerated astrocyte migration measured using matrigel invasion assays. (**E**) Representative photomicrographs showing the effects PF573228, U0126, or LY294002 on Omniscan–accelerated cell adhesion measured using cell adhesion assays. (**F**) Quantitative analysis of the effects of PF573228, U0126, or LY294002 on GBCAs–accelerated adhesion of astrocytes measured by cell adhesion assays. The total number of cells was quantified using ImageJ software (NIH). Bars represent 50 μm. Data are expressed as the mean ± SEM of at least three independent experiments. ****p < 0.0001, indicates statistical significance were analysed by two-way ANOVA continued with post hoc Bonferroni’s or Turkey test compared with the control. ^####^*p* < 0.0001, ^##^*p* < 0.01, ^#^*p* < 0.05, indicates statistical significance were analysed by two-way ANOVA continued with post hoc Bonferroni’s or Turkey.

Interaction of integrin αvβ3 with their ligands results in activation of downstream signaling pathways, promoting changes in protein expression levels or the phosphorylation process. Activation of integrin αvβ3 can induce FAK, ERK1/2, and Akt phosphorylation signaling to activate these signaling cascades, some of which may be involved in inducing F-actin rearrangement. To examine the downstream targets of GBCAs-activated integrin αvβ3, we performed immunocytochemistry and Western blot analysis to measure phosphorylation of FAK, ERK1/2, and Akt in U87MG cells. Representative photomicrograph images of cell immunofluorescence with p-FAK (Y397), pERK1/2, or pAkt, and Phalloidin-iFlour 594 or 488 reagent are shown (Supplementary Fig. S3). GBCA exposure increased p-FAK (Y397), p-ERK1/2, or p-Akt concurrent with increased cortical F-actin formation (Supplementary Fig. S3). To examine further the mechanism of GBCA–induced cell migration, we treated astrocytes with inhibitors of FAK (PF573228), ERK1/2 (U0126), or phosphatidylinositol 3-kinase (PI3K) (Upstream factor for Akt) (LY294002) before performing wound healing, invasion, and focal adhesion assays. We found that coexposure of GBCAs with PF573228, U0126, or LY294002 significantly reduced cell migration in wound healing assays (Fig. 5A–B). However, in the 3D migration (Fig. 5C–D) or focal adhesion assays (Fig. 5E–F), we found a significant difference only with PF573228 or LY294002. These results suggest that the activation of FAK, ERK1/2, and PI3K by GBCAs-integrin αvβ3 interaction is necessary to promote 2D migration. However, only the activation of FAK and PI3K by GBCAs-integrin αvβ3 interaction plays a significant role in focal adhesion or 3D migration. Taken together, the contribution of the ERK 1/2-mediated pathway on GBCA-activated astrocyte migration may be relatively weaker than other pathways.

**Figure 6:**
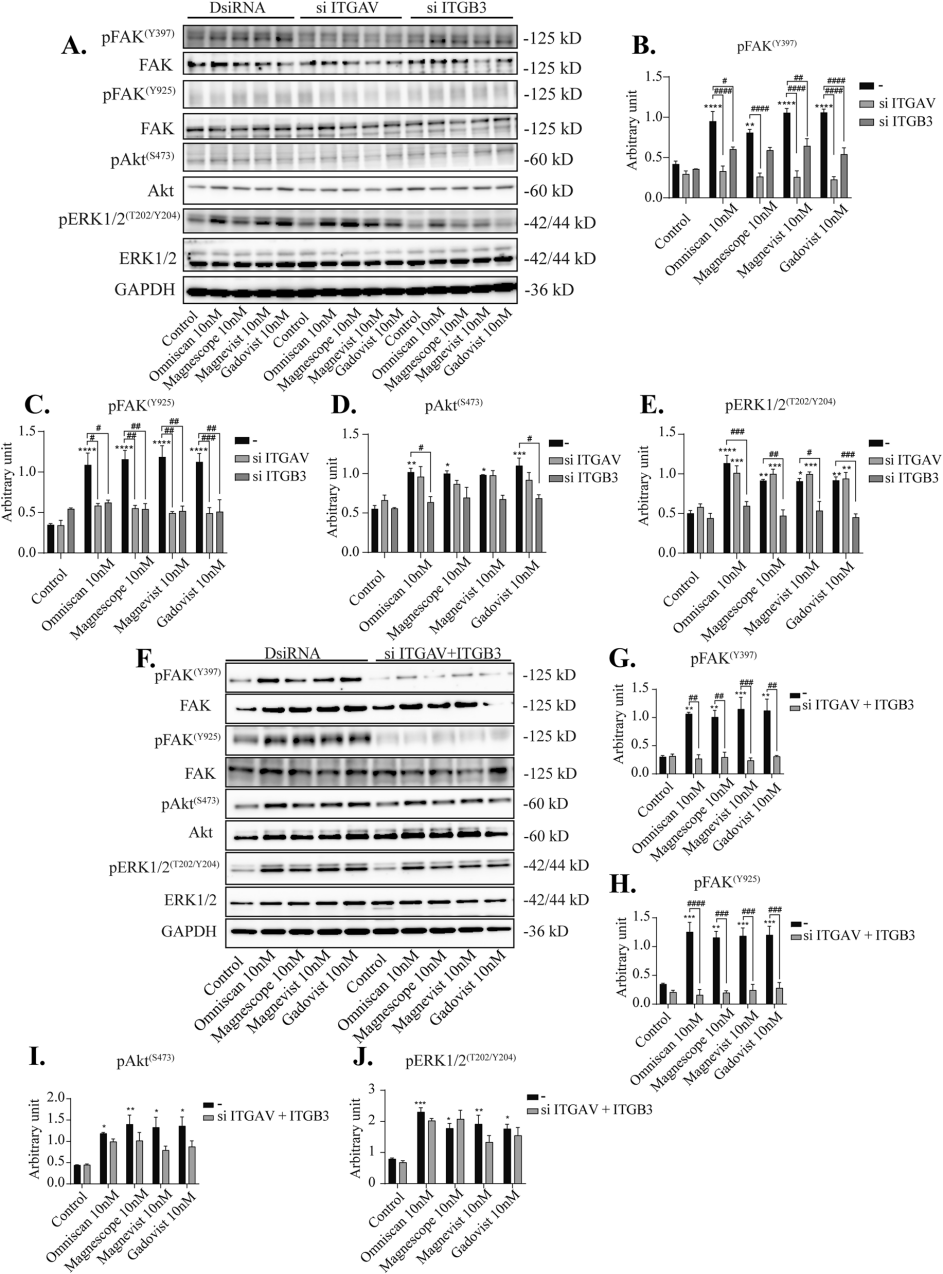
Effects GBCAs on phosphorylation of FAK, Akt, and ERK1/2. The U87MG clonal cells were transfected with DsiRNA or siRNA of ITGAV and/or ITGB3, then exposed to GBCAs for 30 min after serum-starvation for 6 h. (**A**) Representative blots of pFAK, FAK, pAkt, Akt, pERK1/2 and ERK1/2 levels of GBCAs exposure after knockdown of ITGAV or ITGB3. Quantitative analysis of GBCAs effect on the protein expression levels of FAK (Y397) (**B**), FAK (Y925) (**C**), Akt (S473) (**D**), and ERK1/2 (T202/Y204) (**E**) after knockdown of ITGAV or ITGB3. (**F**) Representative blots of pFAK, FAK, pAkt, Akt, pERK1/2 and ERK1/2 levels of GBCAs exposure after knockdown of ITGAV and ITGB3. Quantitative analysis of GBCAs effect on the protein expression levels of FAK (Y397) (**G**), FAK (Y925) (**H**), Akt (S473) (**I**), and ERK1/2 (T202/Y204) (J) after knockdown of ITGAV and ITGB3. Full-length blots are available in supplemental information. The blots were quantified using Fiji ImageJ software (NIH). Data are expressed as the mean ± SEM of at least three independent experiments. ****p < 0.0001, ***p < 0.001, **p < 0.01, *p < 0.05, indicates statistical significance were analysed by two-way ANOVA continued with post hoc Bonferroni’s or Turkey test compared with the control. ^####^*p* < 0.0001, ^###^*p* < 0.001, ^##^*p* < 0.01, ^#^*p* < 0.05, indicates statistical significance were analysed by two-way ANOVA continued with post hoc Bonferroni’s or Turkey.

To further elucidate the molecular mechanisms of astrocyte migration, we performed Western blot analysis after knockdown of integrin αv, β3, or αvβ3, or coexposure with integrin inhibitor cRGDfV. We found that phosphorylation levels of FAK (Y397) were significantly decreased by knockdown of integrin αv, β3, or αvβ3 (Fig. 6A–J). We also examined the other phosphorylation sites for FAK (Y925) and found that the phosphorylation levels were decreased after double knockdown integrin αvβ3 or coexposure with cRGDfV (Fig. 6F–J and Supplementary Fig. S5). On the other hand, no significant changes in the phosphorylation levels of Akt (S473) by knockdown of integrin αv, β3, or αvβ3 were noted, whereas coexposure with cRGDfV significantly reduced GBCA-induced phosphorylation levels of Akt(S473) (Supplementary Fig. S5), indicating the involvement of integrins other than αvβ3. On the other hand, GBCA–induced phosphorylation levels of ERK1/2 (T202/Y204) were significantly decreased by knockdown of integrin β3 subunit or coexposure with cRGDfV, whereas it was not altered by knockdown of integrin αv or αvβ3 (Fig. 6A–J and Supplementary Fig. S5), again indicating the involvement of integrins other than αvβ3. In addition, knockdown of integrin αvβ3 or co-exposure with the inhibitor did not affect the basal levels of phosphorylation protein. Taken together, FAK may be a target for GBCA action mediated by integrin αvβ3, leading to cell migration. In addition, GBCAs may also induce other signal transduction pathways through another integrin subunit or membrane receptor that increases the phosphorylation levels of Akt or ERK1/2.

### GBCAs increased phosphorylation of several RhoGTPase family proteins and induced astrocyte migration

**Figure 7:**
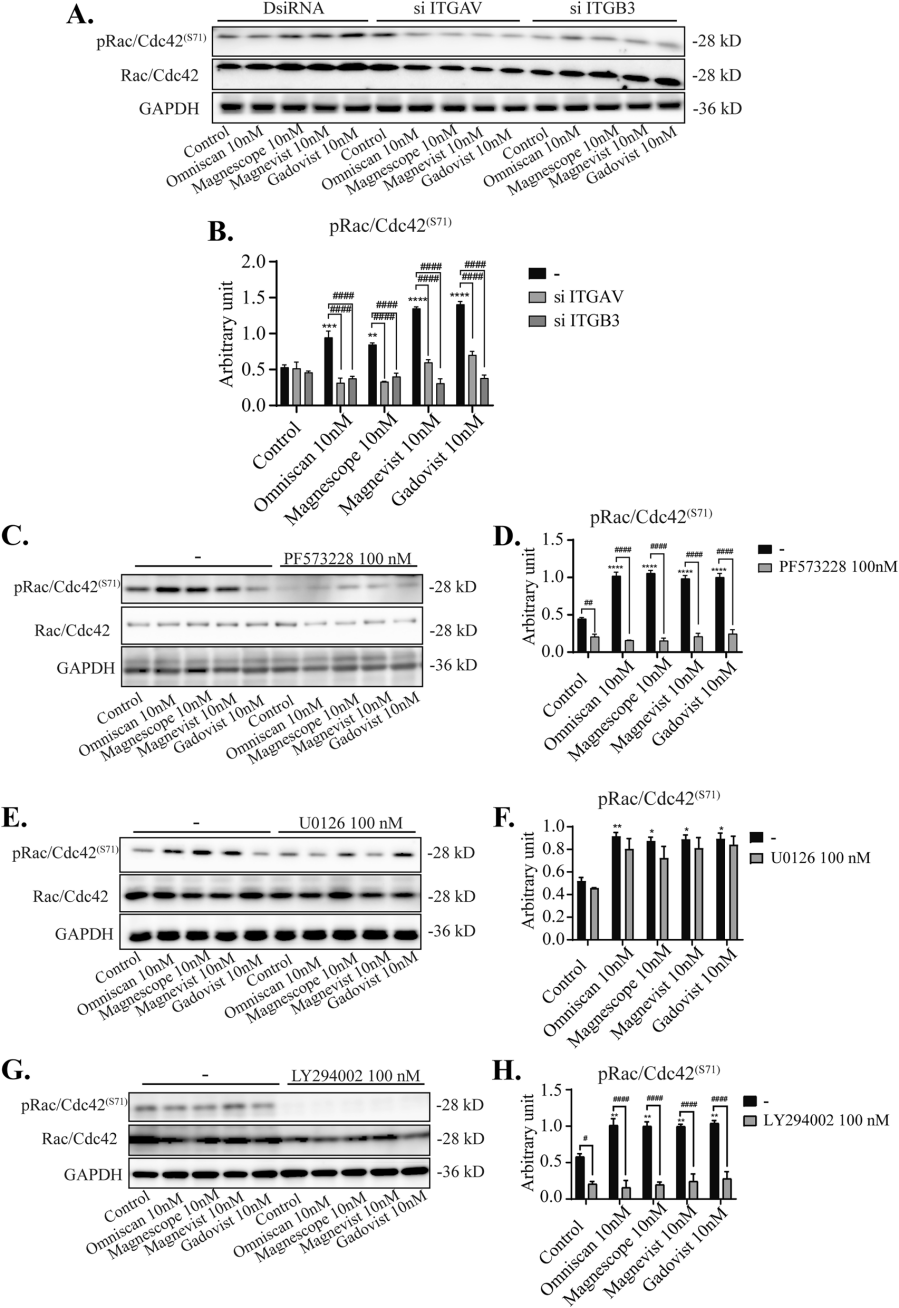
Effect of GBCAs on phosphorylation of RhoGTPase. The U87MG clonal cells were transfected with DsiRNA or siRNA of ITGAV and/or ITGB3, then exposed to GBCAs for 30 min after serum-starvation for 6 h. Representative blots of pRac1/Cdc42 levels of GBCAs exposure after knockdown of ITGAV or ITGB3 (**A**) or coexposure with PF573228 (**C**), U0126 (**E**), or LY294002 (**G**). Quantitative analysis of GBCAs effect on the protein expression levels of Rac1/Cdc42 (S71) after knockdown of ITGAV or ITGB3 (**B**) or coexposure with PF573228 (**D**), U0126 (**F**), or LY294002 (**H**). Full-length blots are available in supplemental information. The blots were quantified using Fiji ImageJ software (NIH). Data are expressed as the mean ± SEM of at least three independent experiments. ****p < 0.0001, ***p < 0.001, **p < 0.01, *p < 0.05, indicates statistical significance were analysed by two-way ANOVA continued with post hoc Bonferroni’s or Turkey test compared with the control. ^####^*p* < 0.0001, ^#^*p* < 0.05, indicates statistical significance were analysed by two-way ANOVA continued with post hoc Bonferroni’s or Turkey.

**Figure 8:**
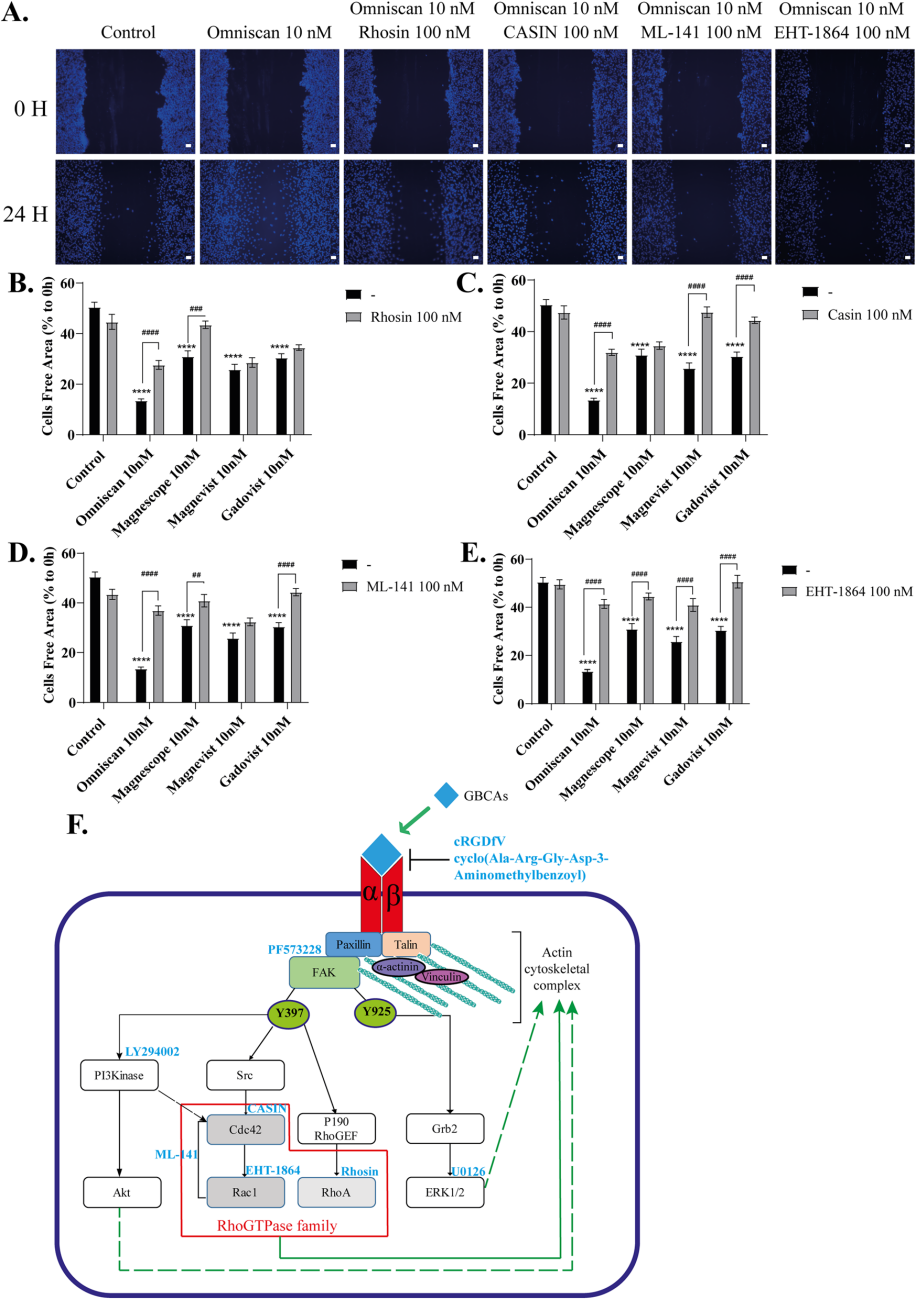
GBCAs increased astrocytes migration via RhoGTPase signalling pathway. (**A**) Representative photomicrographs showing the effects of Rhosin, a RhoA inhibitor, CASIN, a Cdc42 inhibitor, ML–141, a Rac1/Cdc42 inhibitor, and/or EHT-1864, a Rac1 inhibitor, on Omniscan-accelerated C6 cell migration measured using a wound-healing assay. (**D**-**E**) Quantitative analysis of the effects of Rhosin, CASIN, ML–141, and/or EHT–1864 on GBCAs-accelerated cell migration, measured using wound healing assays using C6 cells. (**F**) The proposed mechanism of GBCAs effect in astrocyte migration. Bars represent 50 μm. Data are expressed as the mean ± SEM of at least three independent experiments. ****p < 0.0001, indicates statistical significance were analysed by two-way ANOVA continued with post hoc Bonferroni’s or Turkey test compared with the control. ^####^*p* < 0.0001, ^###^*p* < 0.001, ^##^*p* < 0.01, indicates statistical significance were analysed by two-way ANOVA continued with post hoc Bonferroni’s or Turkey.

Initial cell adhesion and spreading occur in parallel with the activation of several RhoGTPase family proteins such as Rac1, Cdc42, and RhoA, leading to enhanced actin-mediated protrusion. On the other hand, integrin regulates several members of the RhoGTPase family, which control the growth or contraction of F-actin fibers^17^. Thus, we examined the effects of GBCAs on RhoGTPases signaling using Western blot, wound healing, cell migration, and focal adhesion assays. Western blot analysis showed that GBCA-induced phosphorylation levels of Rac1/Cdc42 (S71) were significantly reduced after knockdown of integrin αv, β3, or αvβ3, and coexposure with integrin inhibitor cRGDfV (Fig. 7A–B and Supplementary Fig. S5). Co-exposure with PF573228 or LY294002 also reduced basal level and GBCA-induced phosphorylation levels of Rac1/Cdc42 (S71) significantly, but there were no changes in GBCA-induced phosphorylation levels after coexposure with U0126 (Fig. 7C–H). We then proceeded to examine the roles of RhoGTPases in GBCA-induced cell migration in C6 cells. Representative photomicrograph images of cells stained using live-cell Hoechst staining after the exposure of GBCAs with representative RhoGTPase inhibitor (Fig. 8A). Coexposure of GBCAs and selective RhoA inhibitor, Rhosin (Fig. 8B), Cdc42 inhibitor, CASIN (Fig. 8C), Rac1/Cdc42 inhibitor, ML-141 (Fig. 8D), or Rac1 inhibitor, EHT-1864 (Fig. 8E), significantly decreased GBCA-induced cell migration. A similar trend was also observed in cell migration and focal adhesion assays (Supplementary Fig. S2). These results indicate that GBCA activates phosphorylation of FAK and PI3K through integrin, leading to activation of Rac1/Cdc42 and RhoA resulting in accelerated cell migration.

## Discussion

In our study, we examined the effects of GBCAs on astrocyte migration that is activated by integrin αvβ3 signaling. This study has revealed a novel mechanism of action of GBCAs in cell migration via integrin αvβ3 that may play an important role during brain development and regeneration. We found that GBCAs increased 2D and 3D cell migration as well as focal adhesion of astrocytes. Accelerated cell migration caused by GBCAs was suppressed by knockdown of integrin αvβ3 or coexposure with integrin inhibitor. However, inhibition of integrin αvβ3 did not alter Gd^3+^-mediated migration, indicating that integrin αvβ3 may not be involved in Gd^3+^ action. Thus, we did not use Gd^3+^ for further analysis. GBCA exposure also increased focal adhesion protein, leading to increased cortical actin fibers to induce cell migration. GBCA-integrin interaction also increased phosphorylation of FAK, ERK1/2, and Akt. Knockdown of integrin αv, β3, or αvβ3, and coexposure with integrin inhibitor cRGDfV, significantly suppressed FAK phosphorylation, whereas knockdown of αv did not inhibit the GBCA-mediated phosphorylation of Akt and ERK 1/2. Moreover, coexposure of GBCAs with cyclo (Ala-Arg-Gly-Asp-3-Aminomethylbenzoyl), which is a cyclic RGD peptide and a specific antagonist to integrin αvβ3, only partially blocked GBCAs-induced cell migration. These results indicate the involvement of other factors than integrin αvβ3, such as other integrins containing αv or β3 subunit. While the inhibition of FAK, PI3K, or ERK1/2 phosphorylation suppressed the effect of GBCAs-induced 2D migration of astrocytes (Fig. 5A,B), the inhibition of FAK and PI3K, but not ERK 1/2, suppressed 3D migration (Fig. 5C,D). Furthermore, phosphorylation levels of RhoGTPase signaling, including Rac1 and Cdc42, which play a major role in F-actin assembly, were increased by GBCAs. Such responses were suppressed by either inhibition of PI3K or FAK (Y397) phosphorylation, indicating that Rac1 and Cdc42 proteins are the downstream factors for Integrin-FAK on GBCA-mediated signal transduction. Furthermore, suppression of phosphorylation of RhoGTPase family proteins (Rac1, Cdc42, and RhoA) significantly suppressed C6 cell migration, indicating the involvement of these factors on astrocyte migration. Our results have demonstrated the novel mechanism of action of GBCAs in promoting astrocyte migration via the integrin αvβ3 signaling pathway by modulating the phosphorylation of downstream signal proteins.

It is well known that integrin αvβ3 plays an essential role during focal adhesion and migration. During focal adhesions, integrin clusters can recruit up to 160 different proteins, which form a physical bridge between integrin αvβ3 engaged with the ECM and actin cytoskeletal^35^. Talin and paxillin are integrin–binding proteins that recruit FAK and vinculin to focal contacts^16^. Activated FAK then phosphorylates α-actinin, a cytoskeletal protein that binds to vinculin and crosslinks actomyosin stress fibers and tethers them to focal contacts. In the absence of talin or paxillin, as in the absence of integrins, focal adhesion cannot be performed properly^16,42,45^. GBCA–integrin αvβ3 interaction has been shown to induce integrin αvβ3 clustering and increase the expression level of focal adhesion proteins (Supplementary Fig. S3 and Fig. 4). These findings indicate that exposure to GBCA may influence integrin αvβ3 to form focal adhesions and cause accelerated migration of astrocytes.

FAK functions as a receptor-proximal regulator of cell motility. FAK phosphorylation at Tyr397 can activate FAK–Src complex–mediated recruitment and phosphorylation of the scaffolding protein p130Cas then promote membrane extension on cell migration^16,17^. Activated Src also phosphorylates FAK at Tyr925, which creates an SH2-binding site for GRB2 adaptor protein, leading to activation of Ras and ERK2/mitogen-activated protein kinase (MAPK) cascade^16^. GBCAs increased phosphorylation levels of FAK (Y397), FAK (Y925), and ERK1/2 (T202/Y204). GBCA–increased phosphorylation levels of FAK(Y397) and FAK (Y925) were significantly reduced after knockdown of integrin αvβ3 or coexposure with cRGDfV. However, GBCA–induced increased phosphorylation levels of ERK1/2 (T202/Y204) that is located downstream of FAK (Y925) only reduced after knockdown of integrin β3 subunit or coexposure with cRGDfV, but not by knockdown of integrin αv. Thus, we hypothesized that the increase in phosphorylation levels of ERK1/2 (T202/Y204) might have occurred independently from FAK (Y925) phosphorylation through the activation of other integrins. This could also be a possible reason why inhibition of ERK 1/2 phosphorylation only partially inhibited GBCA–activated 3D migration (Fig. 5C,D) and cell adhesion (Fig. 5E,F), both of which were significantly inhibited by the knockdown of either β3, or αβv3 (Fig. 6).

PI3K/Akt axis is widely known to induce actin filament remodelling and cell migration^46,47^. The association of FAK with PI3K in response to integrin activation has been demonstrated in migration of astrocytes^14,48^. In our study, however, no significant changes in GBCA-induced phosphorylation levels of Akt (S473) after knockdown of integrin αvβ3 (Fig. 6) were observed, whereas the GBCA-induced phosphorylation levels of Akt (S473) were significantly reduced after coexposure with cRGDfV, indicating the involvement of other integrins on the GBCA-induced Akt phosphorylation. Besides, coexposure of GBCAs with PI3K inhibitor significantly reduced 2D or 3D cell migration and cell adhesion. Furthermore, inhibition of PI3K significantly inhibited GBCA-induced phosphorylation of Rac/cdc42 (Fig. 7G,H), indicating PI3K activates astrocyte migration by activating this cascade, rather than activating Akt and downstream targets. Further studies may be required to clarify the involvement of Akt on GBCA-induced cell migration.

The association of FAK with both activators and/or inhibitors of various small GTPase proteins (Rho, Rac, Cdc42, and Ras) alters the polymerization or stabilization of actin and microtubule filaments. Rac1 and Cdc42 can also control the cellular directionality of migrating cells when activated by the mechanical stretch of E–cadherin and P–cadherin. Rac1 signaling can relay directional information from the external environment to the actin cytoskeleton via E–cadherin mediated mechano-sensing so that the cell can migrate toward the target^49^. These results are consistent with our hypothesis that GBCA–integrins interaction activated FAK that regulates the activity of RhoGTPase to promote F–actin rearrangement resulting in increased astrocyte cell migration.

Recently, in vivo and in vitro model have been used to examine GBCA deposition effects on various subsets of the cells in several regions of the brain. Transmission electron microscopy showed that gadodiamide deposited in the DCN, choroid plexus, and granular layer of cerebellar cortex, whereas gadobenate deposited in the DCN and choroid plexus of the cerebellum^50^. The laser ablation ICP–MS showed the deposition of linear GBCAs (gadopentetate, gadobenate, and gadodiamide) highly deposited in DCN and granular layer of the cerebellar cortex, whereas the macrocyclic GBCAs (gadobutrol, gadoterate, and gadoteridol) distributed throughout the cerebellar without any region–specific accumulation and exhibited low Gd concentrations^51^. Intracerebroventricular injection of gadodiamide or gadopentate dimeglumine showed the hypertrophy and hyperplasia of astrocytes and Bergmann glia, incoordination in walking several hours after injection, and also development of ataxia and seizure^52,53^. Again, these results support the previous hypothesis that Gd deposition may affect various subsets of cells in the brain, including glial cells. However, the mechanisms of GBCA toxicity has not yet been clarified. Our present study has shown the possibility that GBCA exposure disrupts integrin αvβ3–activated intracellular signal transduction pathway in the astrocytes and disrupts their function.

In conclusion, our results have shown that exposure to GBCAs accelerated the migration of astrocytes mainly via the integrin αvβ3 signaling pathway and subsequent activation of FAK/PI3K/Akt/RhoGTPase signaling pathway, which induces F-actin rearrangement (Fig. 8F). The activation of FAK by GBCAs may also activate the PI3K/Akt and ERK1/2, although such activation may not be exerted through the direct action of the GBCA-integrin αvβ3 pathway. FAK phosphorylation also induces RhoGTPase signalling activity that alters the polymerization or stabilization of actin and microtubule filaments. This present study highlights the novel mechanism of action of GBCAs in the integrin signaling pathway resulting in accelerated migration of astrocytes during brain development or brain injury. Thus it becomes important to use GBCAs very carefully for imaging purpose in order to avoid adverse side effects, particularly in the context of brain development, brain injury, and cerebral infarction. Further studies that provide new information to prevent GBCA-related toxicity, treat existing GBCA-related health issues, guide the use of existing GBCAs, and direct the design of safer MRI contrast agents are essentially needed. However, we may have to be more cautious in repeated administration of GBCAs, particularly during pregnancy and lactation.

## Materials and methods

### Ethics statement

All animal experiment protocol in the present study was ethically reviewed and approved by the Animal Care, and Experimentation Committee, Gunma University (19-075, 4 December 2019). All mice were treated according to Fundamental Guidelines for Proper Conduct of Animal Experiment and Related Activities in Academic Research Institutions under the jurisdiction of the Ministry of Education, Culture, Sports, Science and Technology of Japan. All efforts were made to minimize animal suffering and the number of animals used. The ARRIVE guidelines^54^ were followed in the preparation of this paper. All experiments performed in this study were carried out in accordance with the approved guidelines and regulations.

### Chemicals

Omniscan, Magnescope, Magnevist, and Gadovist (Fig. 1) were obtained from their respective manufacturers and then diluted with distilled water to their final concentrations. Cyclo (Ala-Arg-Gly-Asp-3-Aminomethylbenzoyl) was purchased from Sigma (St. Louis, MO, USA). cRGDfV, PF573228, LY294002, U0126, CASIN, ML–141, and EHT–1846 were purchased from Cayman Chemical (Ann Arbor, MI, USA). Rhosin HCl was purchased from Tocris Bioscience (Avonmouth, Bristol, UK). The purity of all chemicals was > 98%.

### Clonal cell culture

C6 rat glioma^55^ and U87MG human astrocytoma^56^ clonal cells were obtained from ATCC (Virginia, USA) and maintained in Dulbecco's modified Eagle's medium (DMEM) supplemented with 10% fetal bovine serum (FBS) and antibiotics (100 U/mL penicillin and 100 µg/mL streptomycin) at 37 °C with 5% CO_2_. It should be noted that, although it would be identical to use the same subset of cells to examine the effect of GBCAs, there is a limitation to use primary cultured astrocytes. Wound healing assay cannot be performed using primary cultured astrocytes because the migration speed is very slow. Thus, we had to use C6 cells. On the other hand, to examine the signal transduction pathway, a Western blot for phosphoproteins needs to be conducted. However, most antibodies were generated against human proteins. Thus, we had to use U87MG cells. We have confirmed that GBCAs increased cell invasion of all three cell types, indicating that the effect of GBCA on these cells is comparable (Supplementary Fig. S1).

### Primary culture of mouse cerebral cortex astrocytes

Primary culture of mouse cerebral cortex astrocytes was prepared as previously described^44,57^. Pregnant C57BL/6 strain mice were purchased from Japan SLC (Hamamatsu, Japan). Briefly, postnatal day 1, mouse cerebral cortices were dissected and digested with 2.5% trypsin (Wako, Japan) in Hank's balanced salt solution (Wako) for 30 min with continuous shaking at 37 °C. Cells were resuspended in an astrocyte culture medium (high-glucose Dulbecco's Modified Eagle Medium, 10% heat-inactivated fetal bovine serum, and 1% penicillin/streptomycin), and 10–15 million cells were plated on 10-cm dishes coated with Collagen I (Iwaki, Japan). Cells were incubated at 37 °C in a CO2 incubator. On day 3 in vitro (DIV3), the astrocyte culture medium was replaced with phosphate-buffered saline (PBS). Dishes were then shaken by hand for 30–60 s until only the adherent monolayer of astrocytes was left. The PBS was then replaced with a fresh astrocyte culture medium. Astrocytes were harvested on DIV7 using 0.25% trypsin 1 mM disodium EDTA (Wako) and then plated on 12 or 24 well dishes. Cells were used for cell invasion assay or F-actin staining.

### In vitro wound healing (scratch) assay

The in vitro wound healing (scratch) assay have been described in previous study^44^. C6 cells were plated in a 24-well plates and cultured until confluent. Prior to making a scratch, cells were serum-starved in FBS-free DMEM for 6 h. A wound was created by scratching the monolayer with a 200-µL pipette tip. Floating cells were washed away using PBS. Serum-free DMEM and/or GBCAs, cyclo (Ala-Arg-Gly-Asp-3-Aminomethylbenzoyl), cRGDfV, PF573228, LY294002, U0126, Rhosin, CASIN, ML-141, and/or EHT-1846 were added to the wells and incubated for a further 24 h. At 0 and 24 h, live-cell staining was performed using Cellstain-Hoechst 33,258 solution (Dojindo Molecular Technologies, Inc., Japan) according to the manufacturer's protocol. Images of the scratched area were taken at 0 and 24 h in the same position. The cells were then visualized using a fluorescence microscope (Keyence BZ9000, Keyence Corporation of America, Itasca, IL, USA). Cell migration was determined at the edges of the wound, and the percentage migration was determined as the ratio between migrated distance and initial distance of the wound.

### Matrigel invasion assay

In vitro invasion assays were performed using a 24-well Millicel hanging cell culture insert and a Corning^®^ Matrigel^®^ Matrix according to the manufacturer's instructions (https://www.corning.com/, accessed on 5 January 2022). In brief, astrocytes were seeded at a density of 1 × 10^5^ /mL in serum-free DMEM in the upper chamber. The lower chamber was filled with serum-free DMEM and/or GBCAs, cyclo (Ala-Arg-Gly-Asp-3-Aminomethylbenzoyl), cRGDfV, PF573228, LY294002, U0126, Rhosin, CASIN, ML-141, and/or EHT-1846. After 16–18 h of incubation, noninvading cells in the upper chamber were removed with a sterile cotton swab. The filters from the inserts were fixed with 4% paraformaldehyde (PFA) and stained with DAPI. The cells were then inspected using a laser confocal scanning microscope (Zeiss LSM 880, Carl Zeiss Microscopy GmbH, Jena, Germany). The number of invading cells on the lower surface of the filter was counted.

### Cell adhesion assay

Astrocytes or C6 cells were plated in 35 mm dish until 80% of confluence. Prior to seeding, cells were serum-starved in FBS-free DMEM for 6 h. In brief, astrocytes were seeded at a density of 2 × 10^5^ /mL in serum-free DMEM in the 24-well plate. Wells were filled with serum-free DMEM and/or GBCAs, cyclo (Ala-Arg-Gly-Asp-3-Aminomethylbenzoyl), cRGDfV, PF573228, LY294002, U0126, Rhosin, CASIN, ML-141, and/or EHT-1846. After 2 h of incubation, non-adherent cells were removed together with medium, and the adherent cells were washed with PBS. The cells were fixed with 4% paraformaldehyde (PFA) and stained with cresyl violet, then visualized using a fluorescence microscope (Keyence BZ9000). The number of adherent cells on the well were counted with Fiji ImageJ (NIH).

### Immunocytochemistry analysis of protein phosphorylation and F-actin formation

The immunocytochemistry for phosphorylation protein and F-action formation were described in previous studiesy^43,44,57^. Cultured cells were exposed to GBCA for 30 min, then rinsed three times with PBS, fixed with 4% PFA, and blocked with 2% FBS. Cells were then incubated with rabbit monoclonal anti-phospho-FAK (Y397) XP (1:200; Cell Signaling, MA, USA), anti-phospho-Akt (S473) (D9E) XP (1:200; Cell Signaling), or anti-phospho-p44/42 MAPK (ERK1/2) (T202/Y204) (1:200; Cell Signaling) antibodies, followed by CytoPainter Phalloidin-iFluor 594 or 488 reagents (Abcam) and donkey anti-rabbit IgG (H + L) secondary antibodies, Alexa Fluor® 488 or 594 conjugate (1:200; Thermo Fisher Scientific, Inc, Waltham, MA, USA). Cell nuclei were also stained with DAPI. The cells were then inspected using a laser confocal scanning microscope (Zeiss LSM 880, Carl Zeiss Microscopy GmbH).

### RNA interference assay

Astrocyte-enriched cultures or U87MG clonal cells were transfected with siRNAs for integrin αv (ITGAV) or β3 (ITGB3) (Integrated DNA Technologies, Inc., Coralville, IA, USA), or negative control RNAs (negative control DsiRNA [catalog no. 51-01-14-03; Integrated DNA Technologies, Inc.]), using lipofectamine RNAiMAX reagent (Thermo Fisher Scientific) according to the manufacturer's protocol. The list of siRNA sequences used in this study is listed in Supplementary Fig. 4 (S4). Briefly, siRNA lipid complexes [1 nM of control siRNA (DsiRNA), ITGAV, or ITGB3 siRNA] were incubated for 20 min and then added to astrocytes or U87MG cells at approximately 80% confluency in 35-mm dishes. After 16–24 h, the cells were subjected to matrigel invasion assay or Western blot. The efficacy of the siRNA knockdown was verified by quantitative real-time PCR (qRT-PCR). Total RNA was extracted using *SuperPrep* cell lysis and RT kit for qPCR reagent (TOYOBO Bio-Technology, Japan) according to the manufacturer's instructions. qRT-PCR was performed using THUNDERBIRD SYBR qPCR Mix (TOYOBO) as per the manufacturer's instructions and using a StepOne RT-PCR System (Thermo Fisher Scientific). The list of primers used in this study is listed in Supplementary Fig. 4 (S4). qRT-PCR was performed as follows: denaturation at 95 °C for 20 s, followed by amplification at 95 °C for 3 s and at 60 °C for 30 s (40 cycles). All experiments were repeated three times, using independent RNA preparations to ensure the consistency of the results. All mRNA levels were normalized to that of glyceraldehyde-3-phosphate dehydrogenase (GAPDH). This is done because quantitative expression levels of genes are normalized to the levels of expression of so called controls or housekeeping genes such as GAPDH.

### Western blot analysis

Cultured cells were homogenized in RIPA buffer (Cell Signaling) and protease inhibitors (Complete; Roche, IN, USA). Protein concentration was measured using the Bradford protein assay (Bio-Rad) according to the manufacturer's instructions. After boiling for 5 min, protein samples (5 µg) were subjected to 5%–20% SDS–polyacrylamide Supersep Ace (Wako) gel electrophoresis, and the separated products were transferred to nitrocellulose membranes. Membranes were blocked with 5% nonfat dry milk in Tris-buffered saline containing 0.1% Tween 20, followed by overnight incubation with the appropriate diluted primary antibodies for p-FAK (Y397) or (Y925) (1:1000; Cell Signaling), FAK(1:1000; Cell Signaling), p-ERK1/2 (T202/Y204) (1:1000; Cell Signaling), ERK1/2 (1:1000; Cell Signaling), p-Akt (S473) (1:1000; Cell Signaling), Akt (1:1000; Cell Signaling), p-Rac1/Cdc42 (S71) (1:1000; Cell Signaling), Rac1/Cdc42 (1:1000; Cell Signaling), Talin-1 (1:1000; Cell Signaling), Vinculin (1:1000; Cell Signaling), α-Actinin (1:1000; Cell Signaling), Paxillin (1:1000; Cell Signaling), Cortactin (1:1000; Merck Millipore), and GAPDH (1:1000; Proteintech, IL, USA). After washing with Tris-buffered saline containing 0.1% Tween 20, membranes were incubated with horseradish peroxidase-conjugated anti-rabbit or anti-mouse IgG secondary antibody (1:3000; Cell Signaling) for one h at room temperature and detected using an ECL detection system (Wako). GAPDH was once again used as loading control.

### Statistical analysis

Data are expressed as mean ± SEM of three individual experiments performed in triplicate. One-way or two-way ANOVA followed by post hoc Bonferroni's and Turkey multiple comparison tests were performed using GraphPad Prism version 9.1.1 (GraphPad Software, San Diego, USA, www.graphpad.com) or IBM SPSS Statistics 27 for Windows. All *p*-values < 0.05 were considered statistically significant.

## Supplementary Information

Supplementary Information 1.

Supplementary Information 2.

Supplementary Information 3.

Supplementary Information 4.

Supplementary Information 5.

Supplementary Information 6.

Supplementary Information 7.

Supplementary Information 8.

Supplementary Information 9.

Supplementary Information 10.
