# Supplementary figures and images for: Gadolinium-based contrast agent accelerates the migration of astrocyte via integrin αvβ3 signaling pathway
Source: Sci Rep. 2022 Apr 7;12:5850. doi: 10.1038/s41598-022-09882-7 (PMC8990080; doi:10.1038/s41598-022-09882-7)

A. Cell Invasion Assays in C6 cells

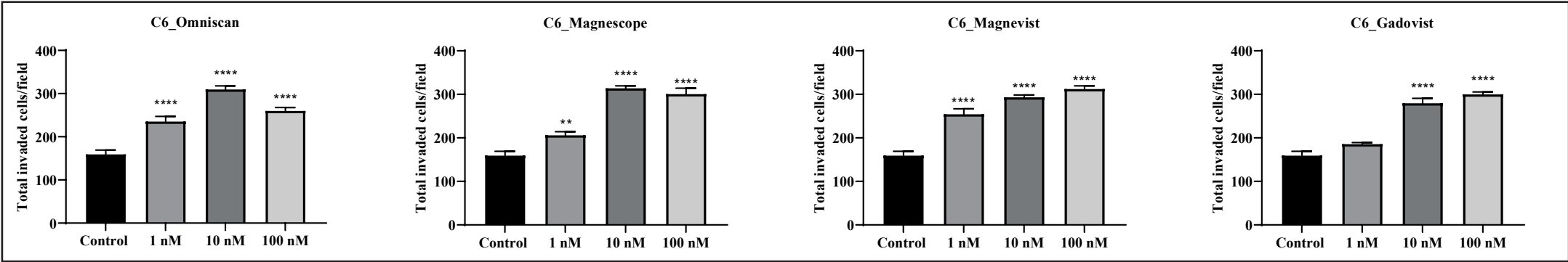

B. Cell Invasion Assays in U87MG cells

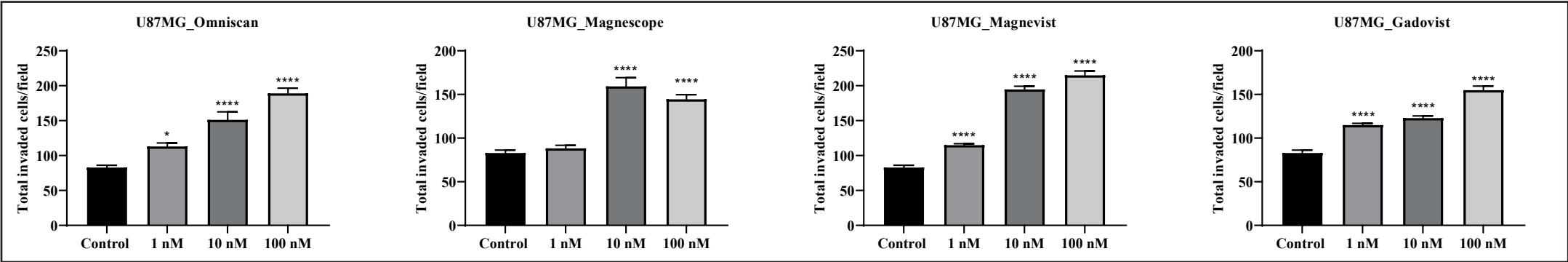

Supplement: Supplementary file 1 — Supplementary Information 1. [file 41598_2022_9882_MOESM1_ESM.pdf]

Supplementary Figure S2.

A. Cell Invasion Assays

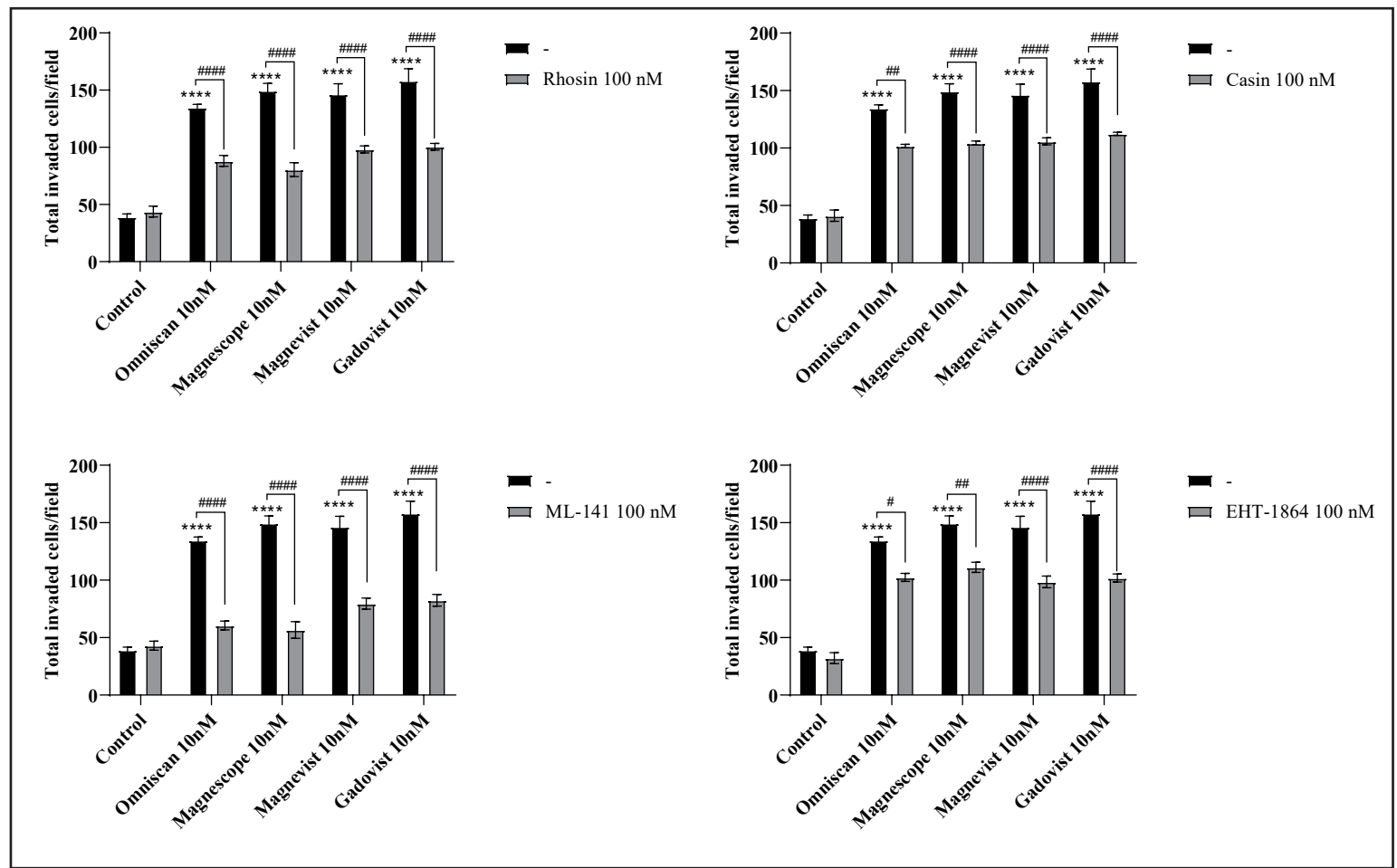

B. Cell Adhesion Assays

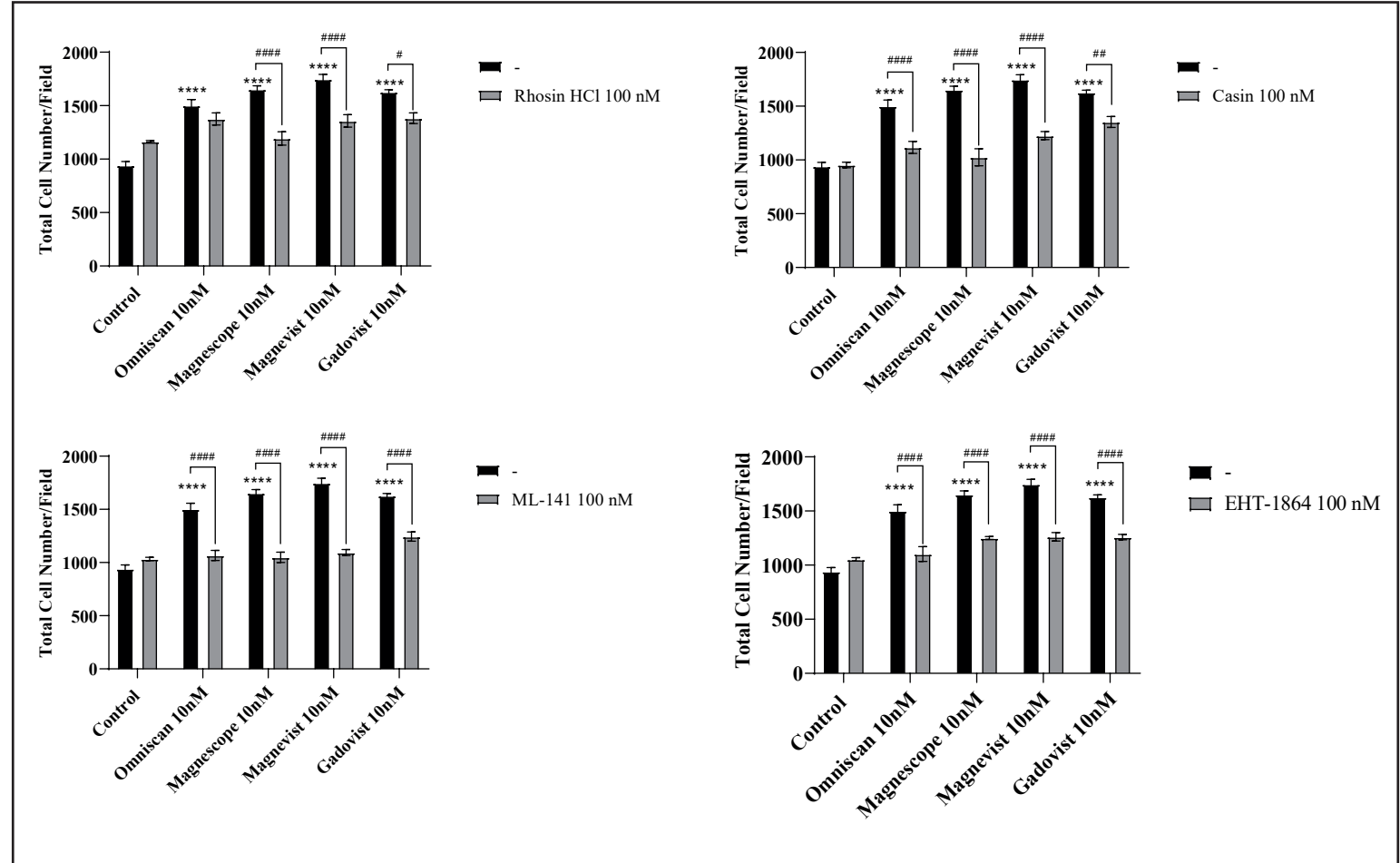

Supplement: Supplementary file 2 — Supplementary Information 2. [file 41598_2022_9882_MOESM2_ESM.pdf]

Supplementary Figure S3.

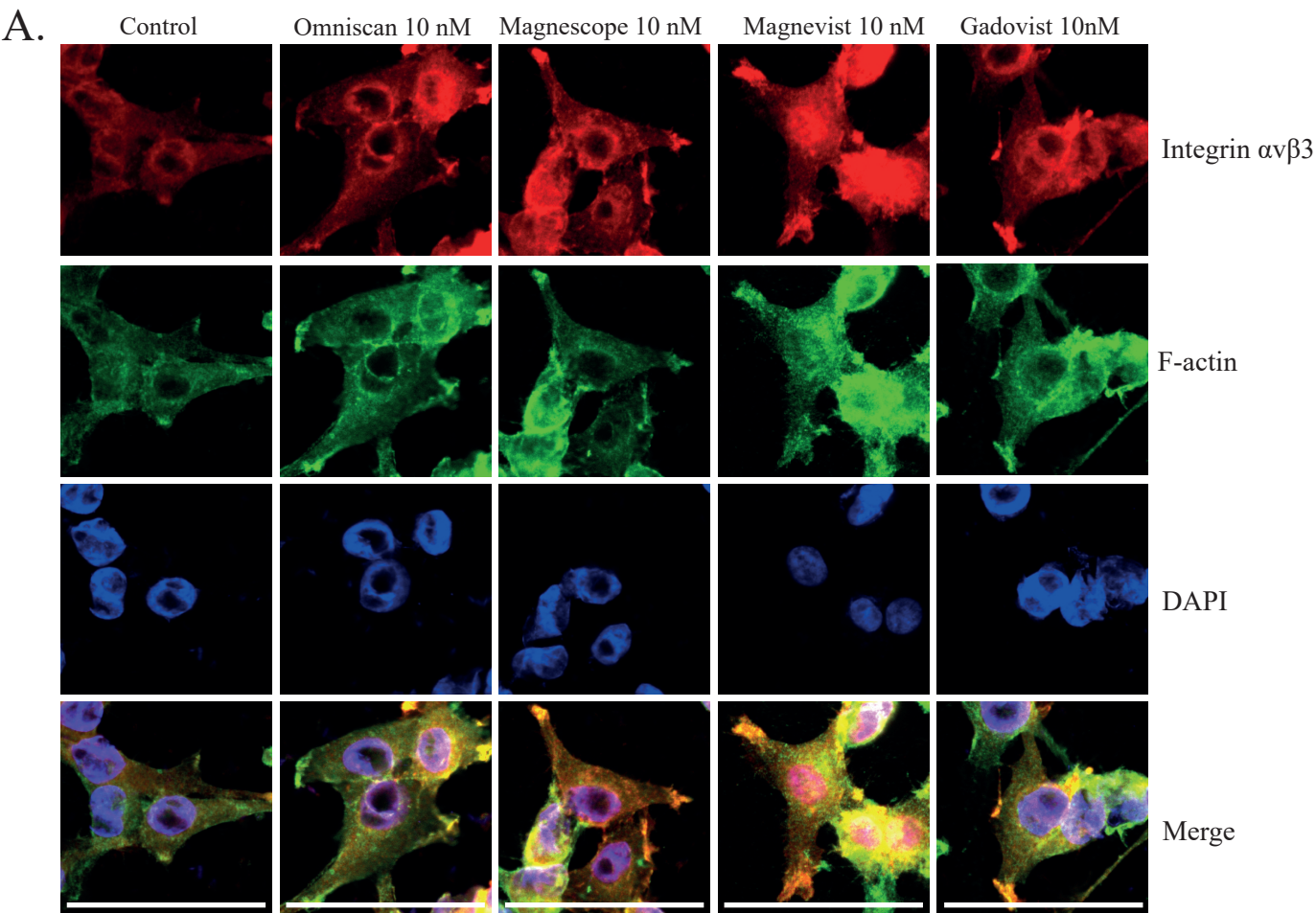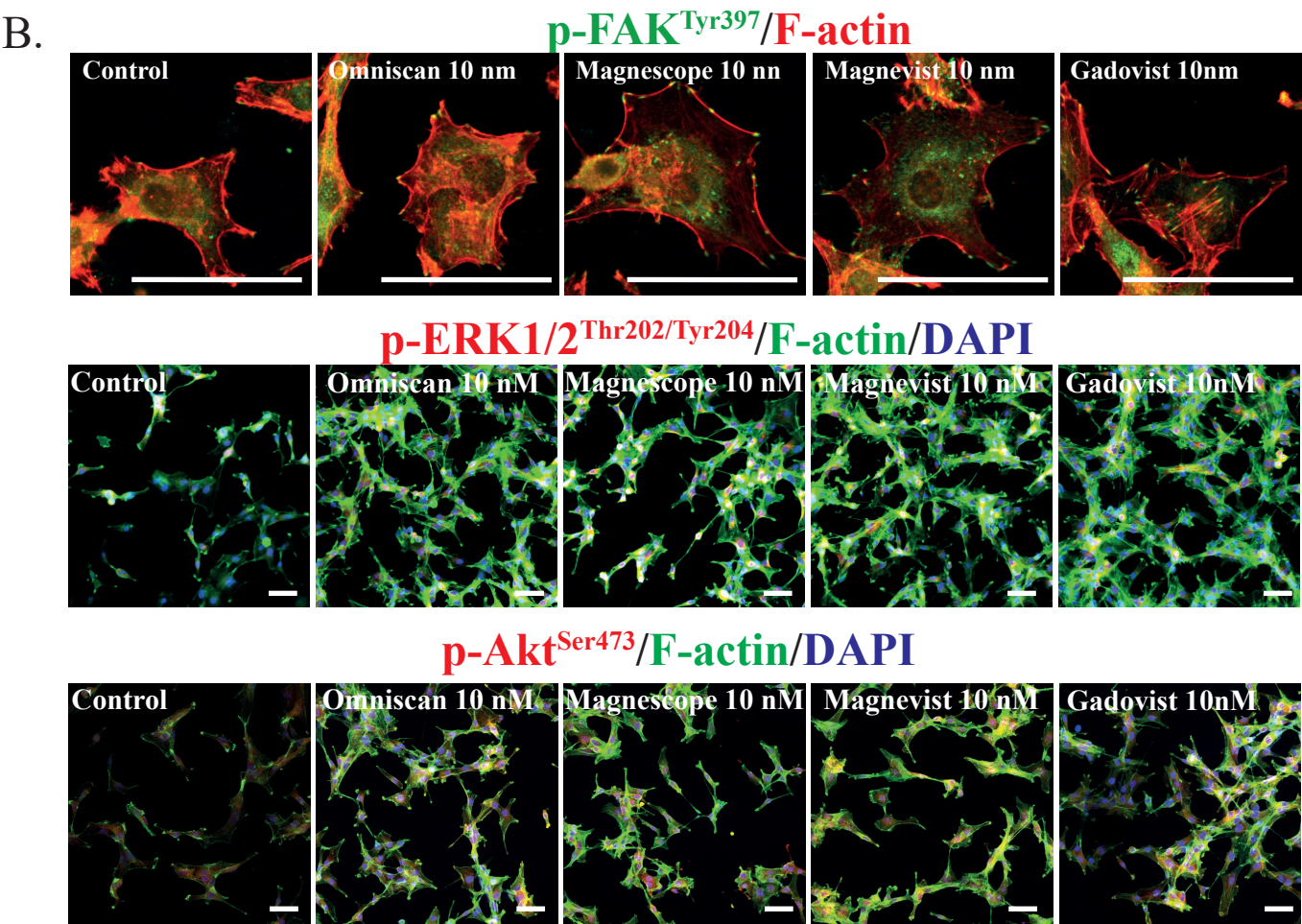

Supplement: Supplementary file 3 — Supplementary Information 3. [file 41598_2022_9882_MOESM3_ESM.pdf]

Supplementary Figure S4.

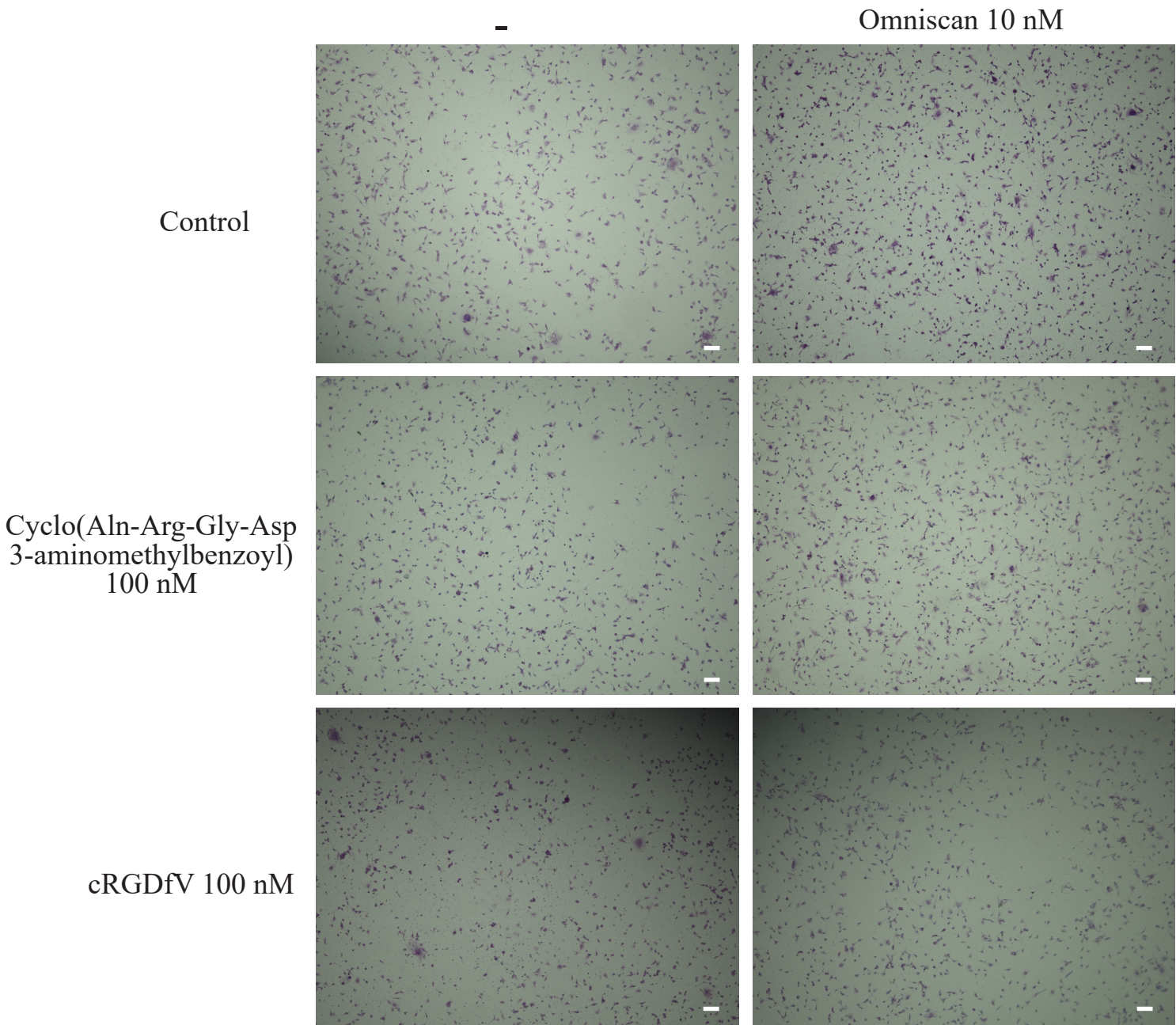

Supplement: Supplementary file 4 — Supplementary Information 4. [file 41598_2022_9882_MOESM4_ESM.pdf]

# Supplementary Figure S6.

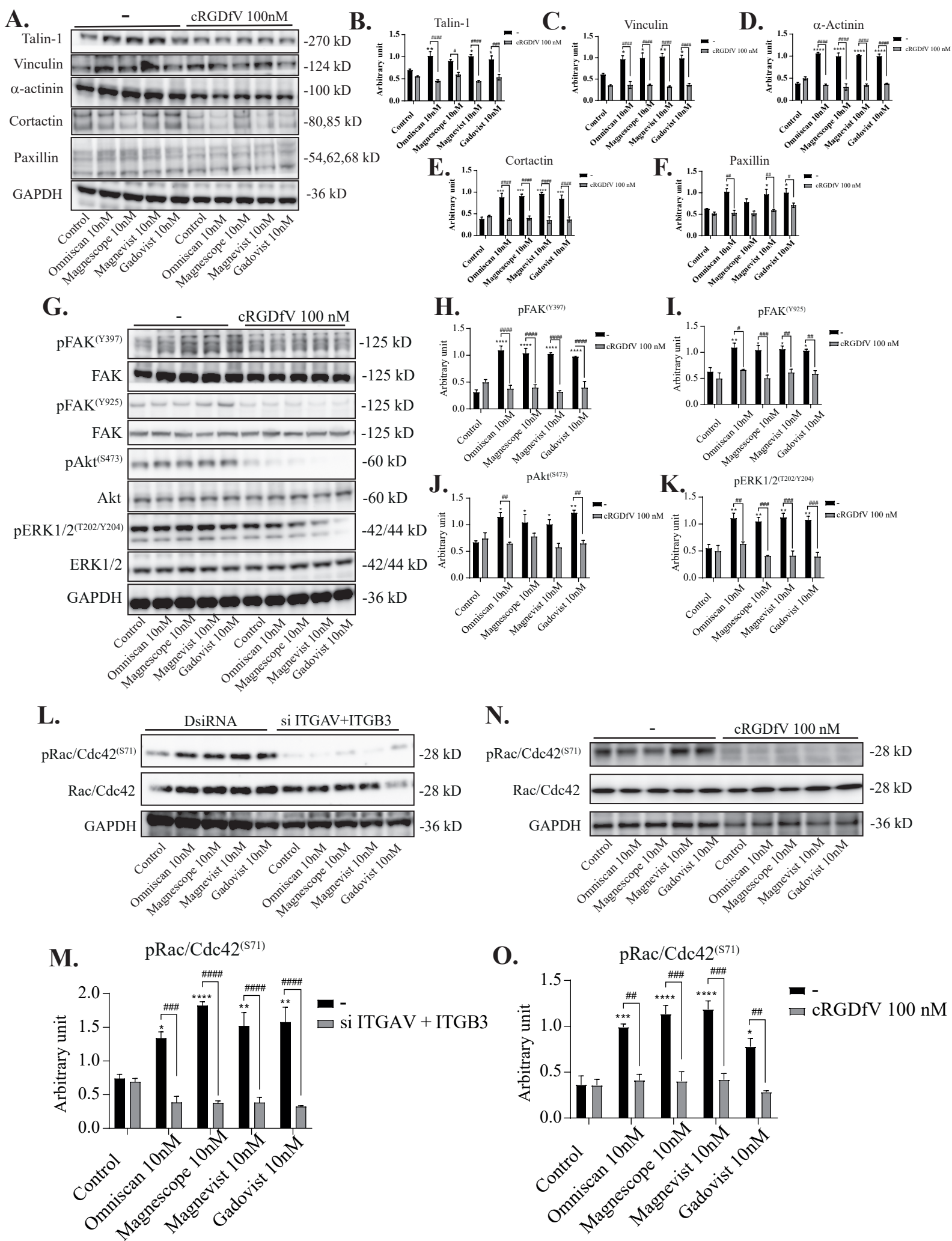

Supplement: Supplementary file 6 — Supplementary Information 6. [file 41598_2022_9882_MOESM6_ESM.pdf]

A. GdCl<sub>3</sub>

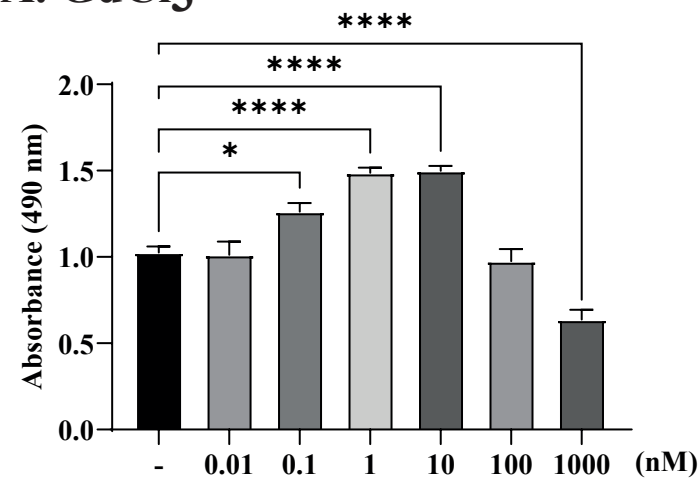

B. Omniscan

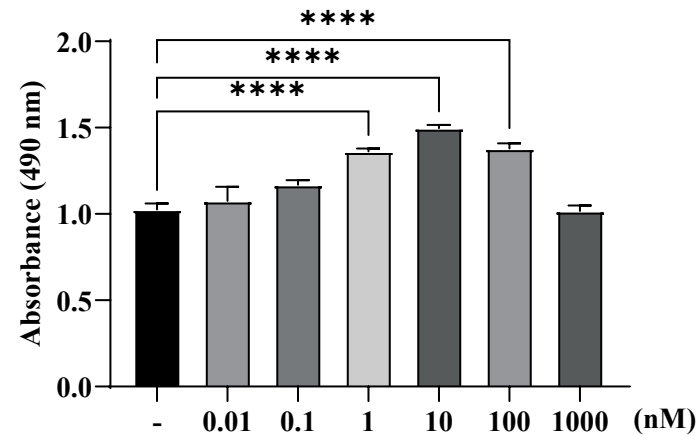

C. Magnescope

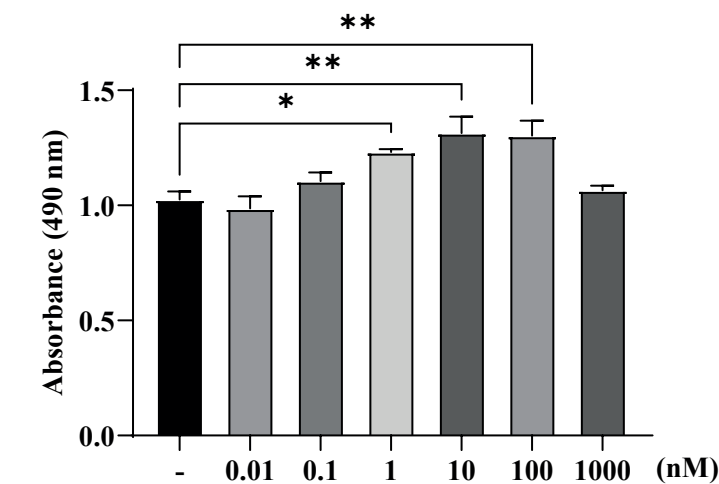

D. Magnevist

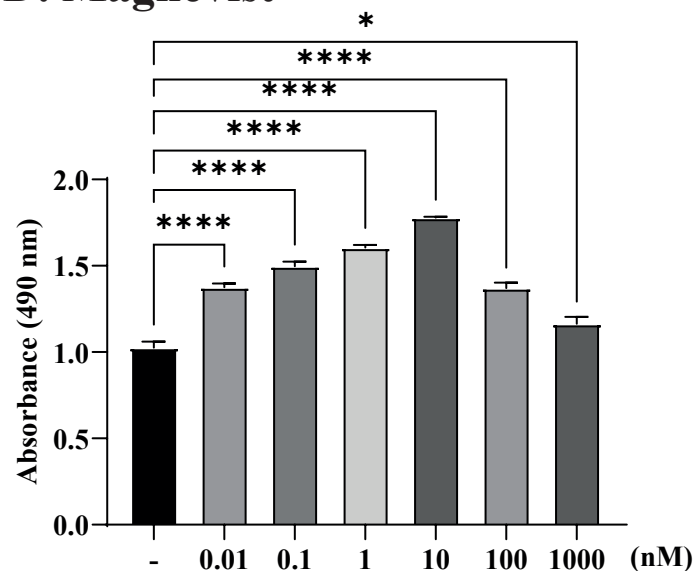

E. Gadovist

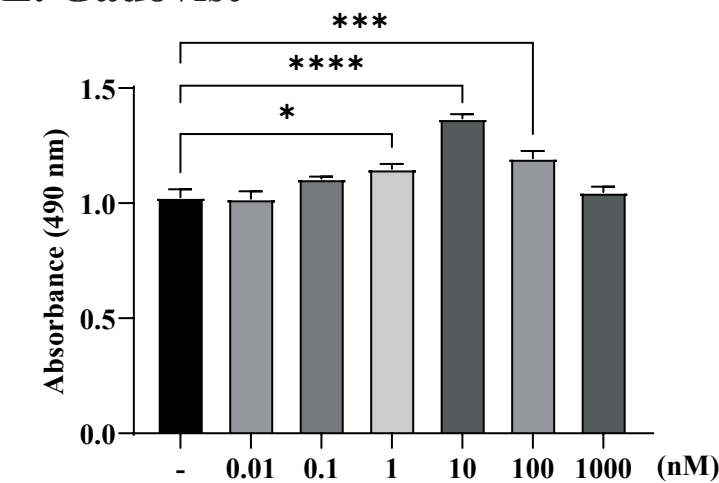

Supplement: Supplementary file 7 — Supplementary Information 7. [file 41598_2022_9882_MOESM7_ESM.pdf]

Supplementary Figure S7.

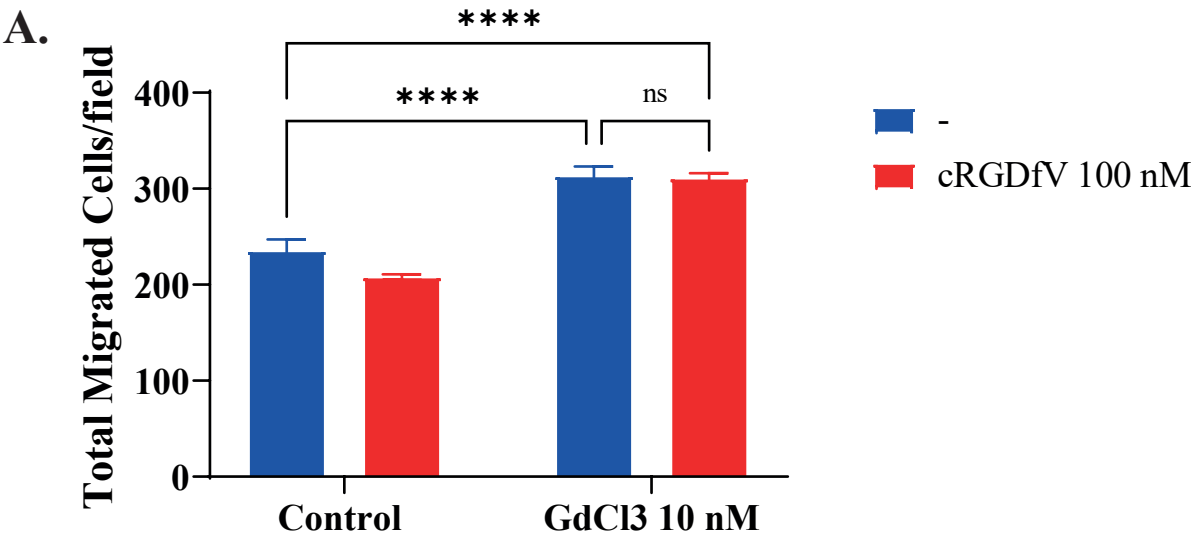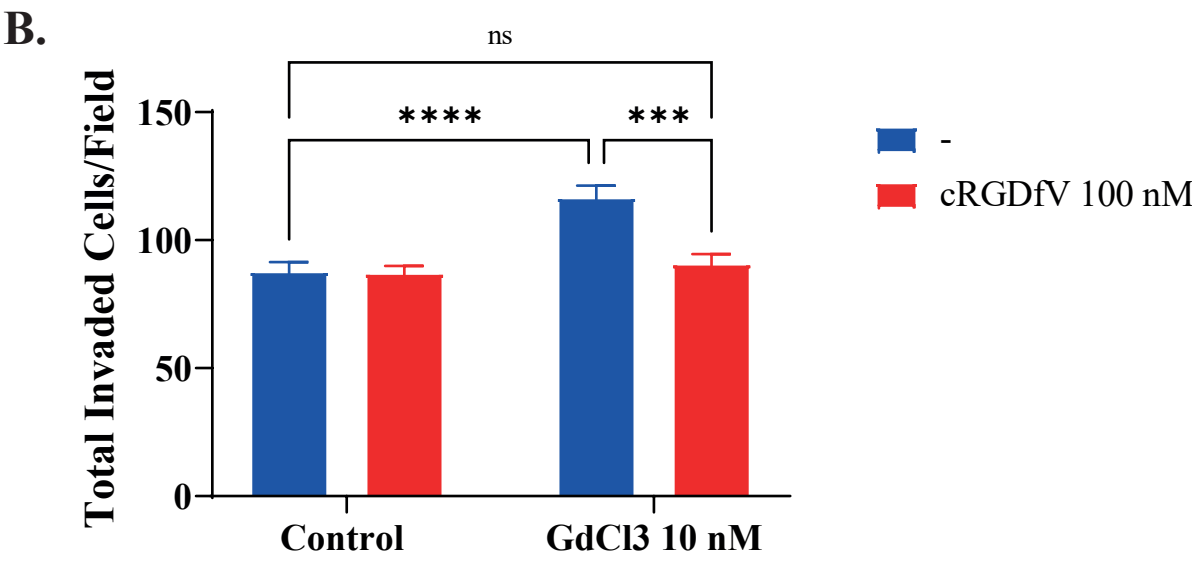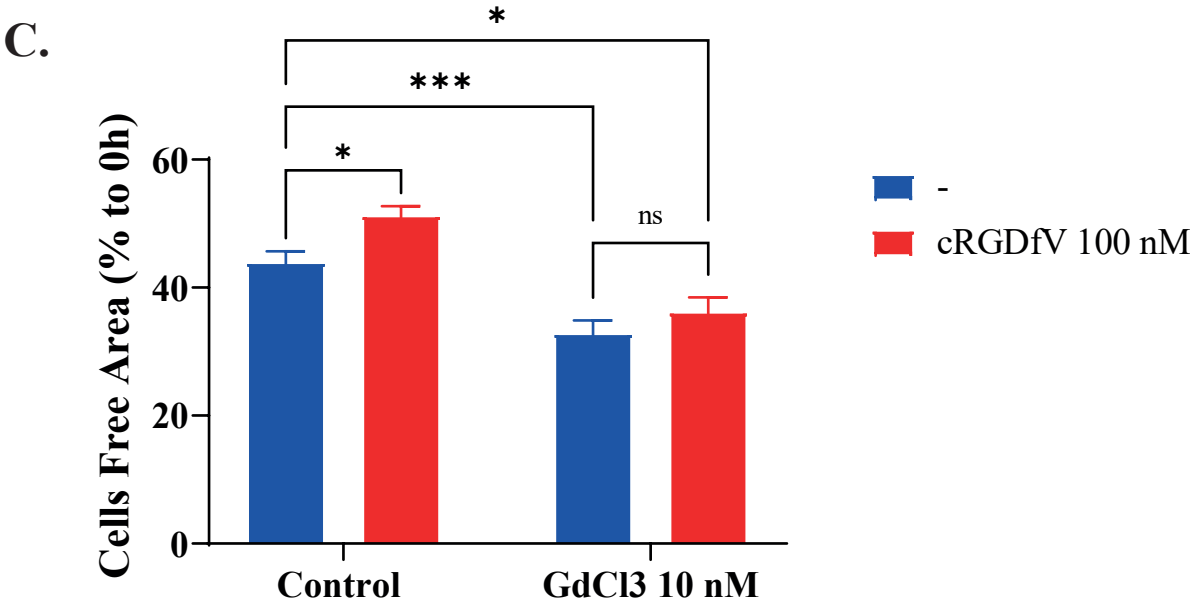

Supplement: Supplementary file 8 — Supplementary Information 8. [file 41598_2022_9882_MOESM8_ESM.pdf]
