# Supplementary material for: Gadolinium-based contrast agent accelerates the migration of astrocyte via integrin αvβ3 signaling pathway
Source: Sci Rep. 2022 Apr 7;12:5850. doi: 10.1038/s41598-022-09882-7 (PMC8990080; doi:10.1038/s41598-022-09882-7)
Supplement: Supplementary file 5 — Supplementary Information 5. [file 41598_2022_9882_MOESM5_ESM.pdf]

Supplementary Figure S5.

A.

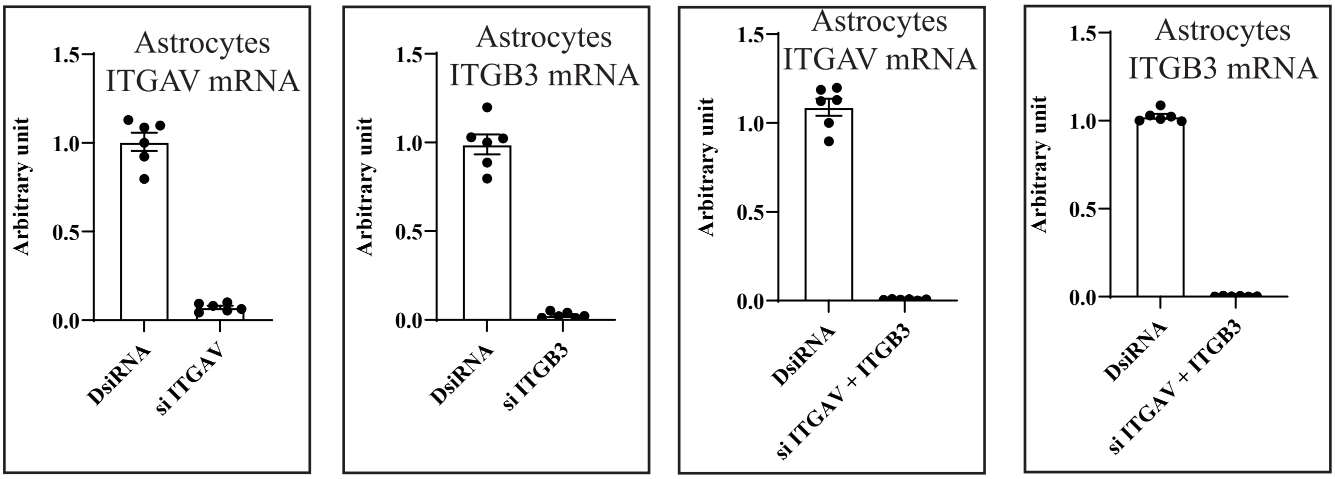

B.

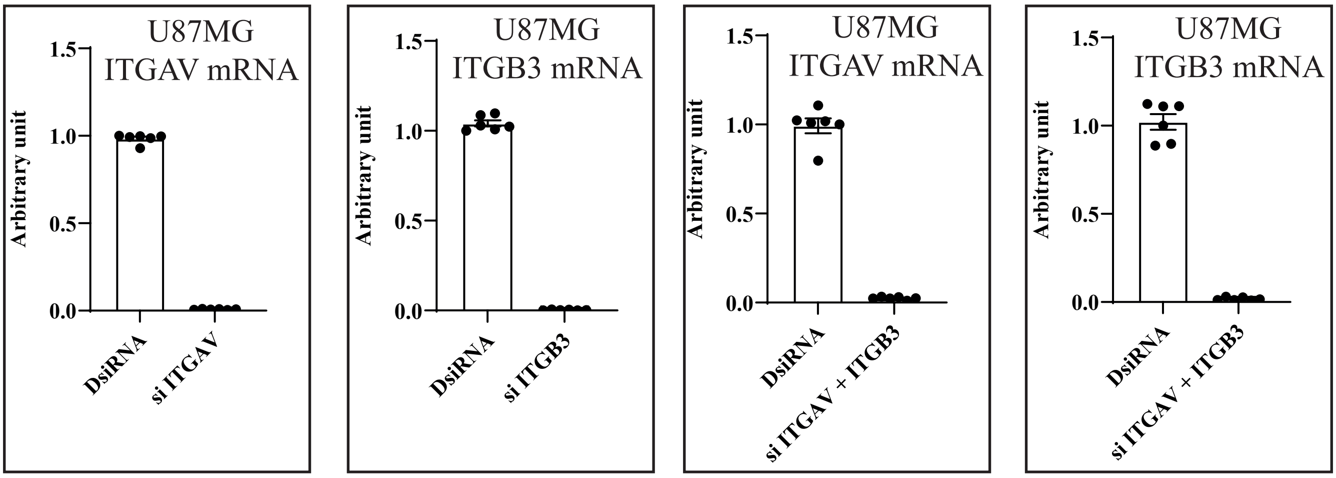

Table 1. siRNA target sequences.

Gene	Sense	Antisense
DsiRNA Controls	CGUUAAUCGCGUAUAAUACGCGUAT	AUACGCGUAUUAUACGCGAUUAACGAC
ITGAV (Mouse)	ACAGUUGACUUCAUAAGC	UUCAGUAGCUUAUGAAGU
ITGB3 (Mouse)	CUAGGCAAGAACAUUACC	AUCAGUUGGUAAUGUUCU
ITGAV (Human)	GACUGAGCUAAUCUUGAG	AUAUAUUCUCAAGAUUAG
ITGB3 (Human)	GGUCUCUUUCAGUAUCAA	UAGAAUGUUGAUACUGAA

Table 2. Primer sequences.

Gene	Sense	Antisense
ITGAV (Mouse)	TGTGAAGGCGCAGAATCAAG	AATGCACAGGACAGTCTTGC
ITGB3 (Mouse)	GGCAAAAACGCCGTGAATTG	TTCCACCACATAGAGGACTGC
GAPDH (Mouse)	TGCGACTTCAACAGCAACTC	ATGTAGGCCATGAGGTCCAC
ITGAV (Human)	TTCTTCCGATTCCAAACTGG	TGCCTTGCTGAATGAACTTG
ITGB3 (Human)	TGGCAGCTGTGTCTGTATCC	CCCCGGTCAAACTTCTTACA
GAPDH (Human)	TGAGTCCTTCCACGATACCA	GATCATCAGCAATGCCTCCT
