# Supplementary material for: Gadolinium-based contrast agent accelerates the migration of astrocyte via integrin αvβ3 signaling pathway
Source: Sci Rep. 2022 Apr 7;12:5850. doi: 10.1038/s41598-022-09882-7 (PMC8990080; doi:10.1038/s41598-022-09882-7)
Supplement: Supplementary file 9 — Supplementary Information 9. [file 41598_2022_9882_MOESM9_ESM.pdf]

Full-Length Blots Original Images

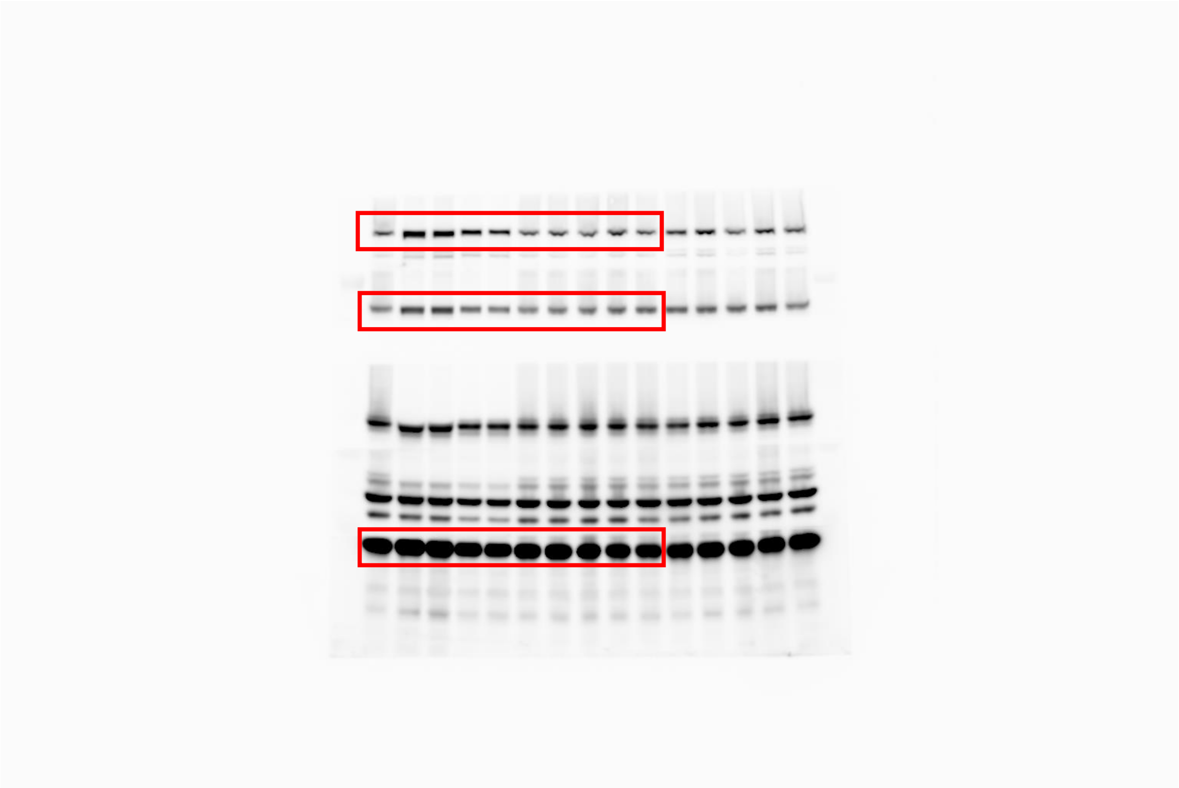

Figure 4I. Talin-1 (270 kD), α-actinin (100 kD) and GAPDH (36 kD)

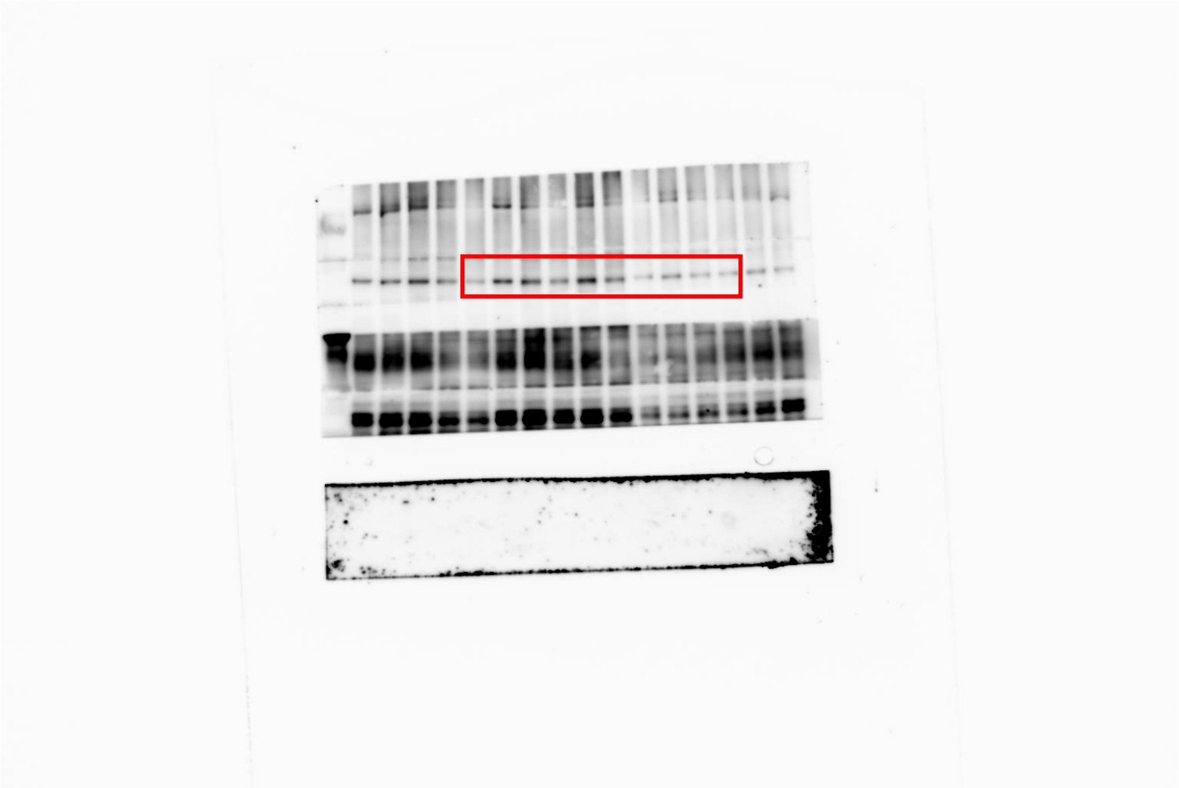

Figure 4I. Vinculin (124 kD)

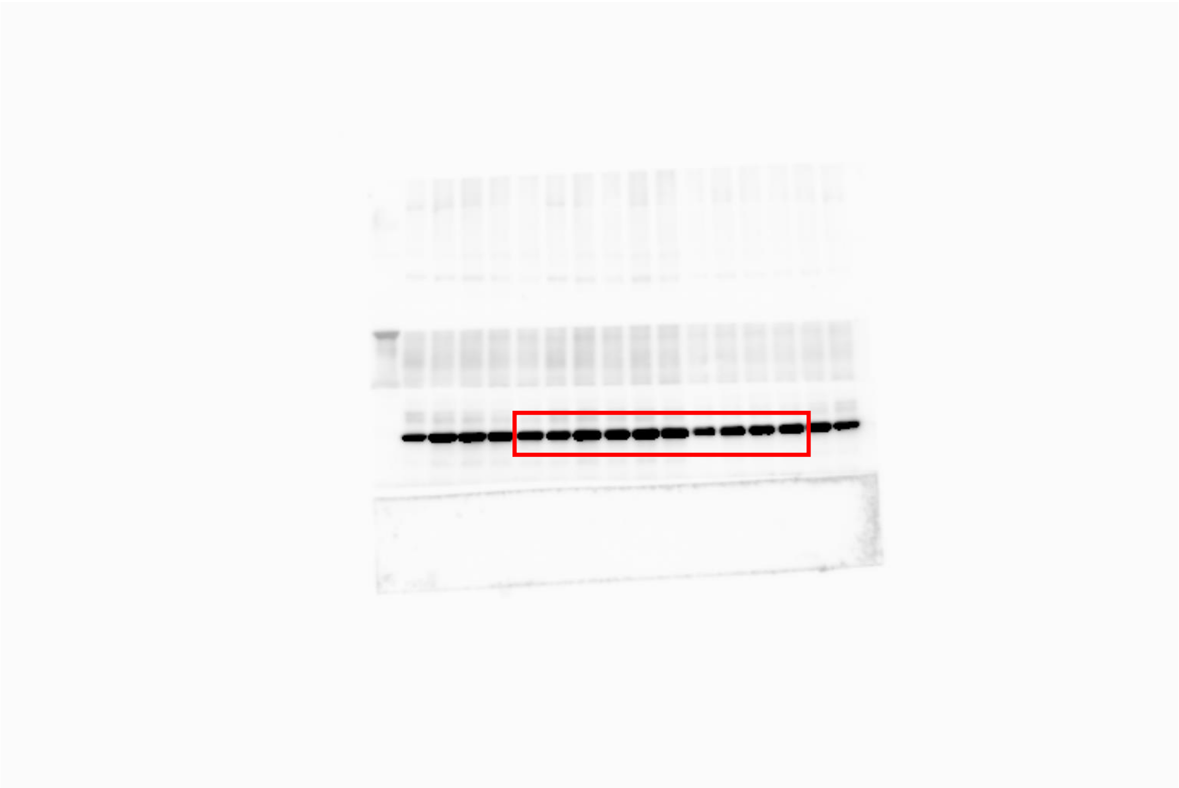

GAPDH (36 kD)

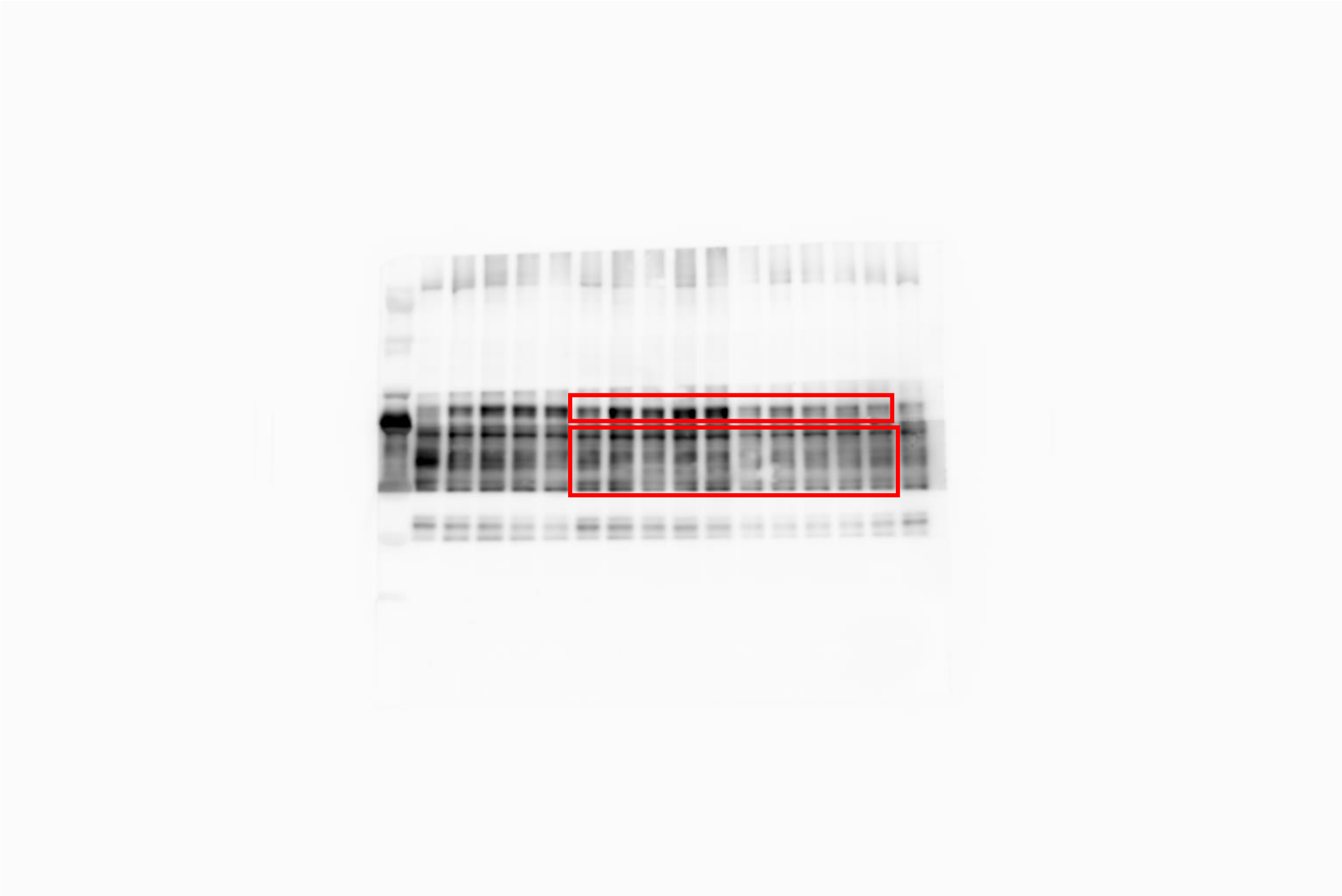

Figure 4I. Cortactin (80,85 kD) and Paxillin (54, 62, 68 kD)

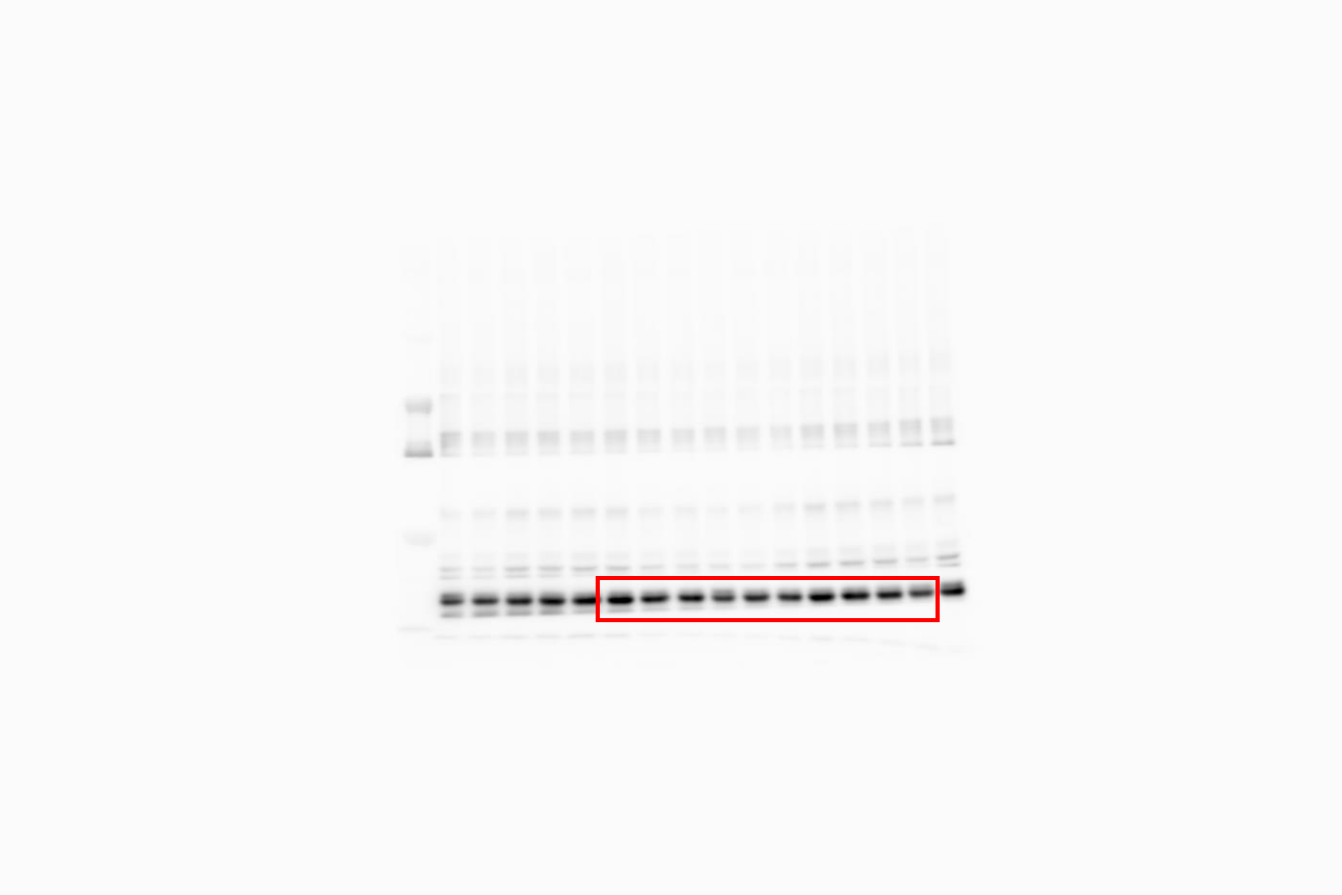

GAPDH (36 kD)

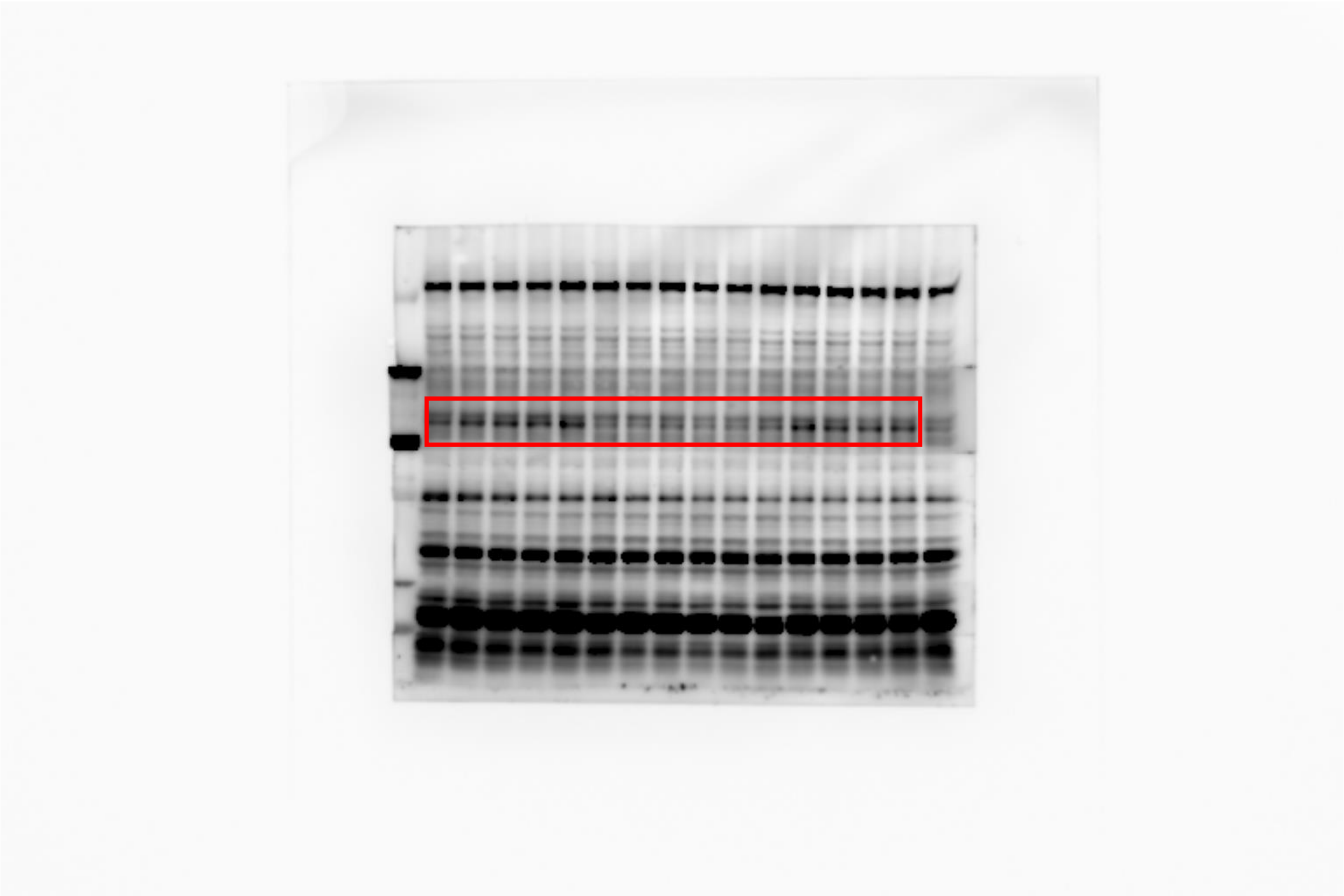
Figure 6A. pFAK (125 kD)

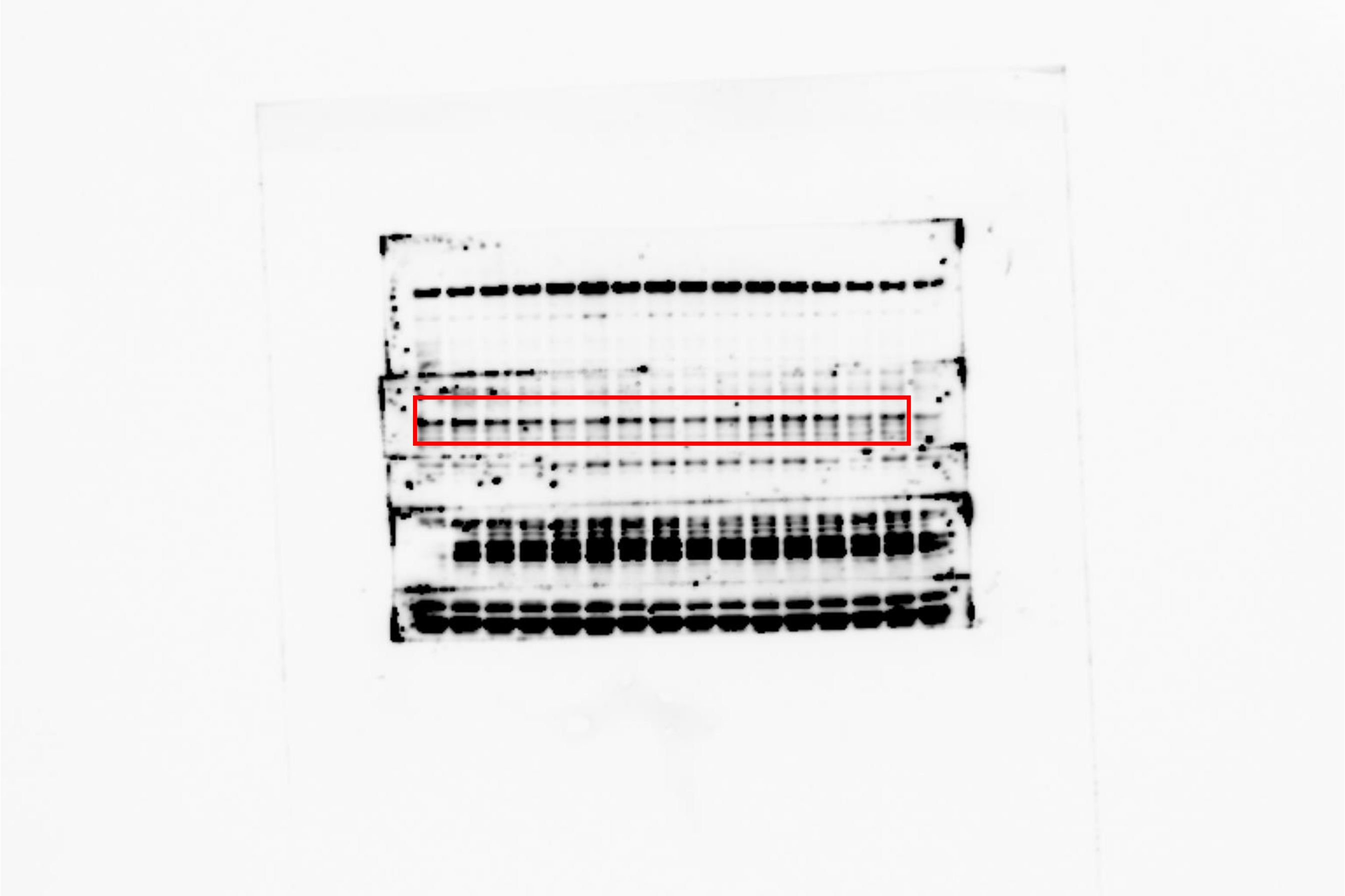
Figure 6A. FAK (125 kD)

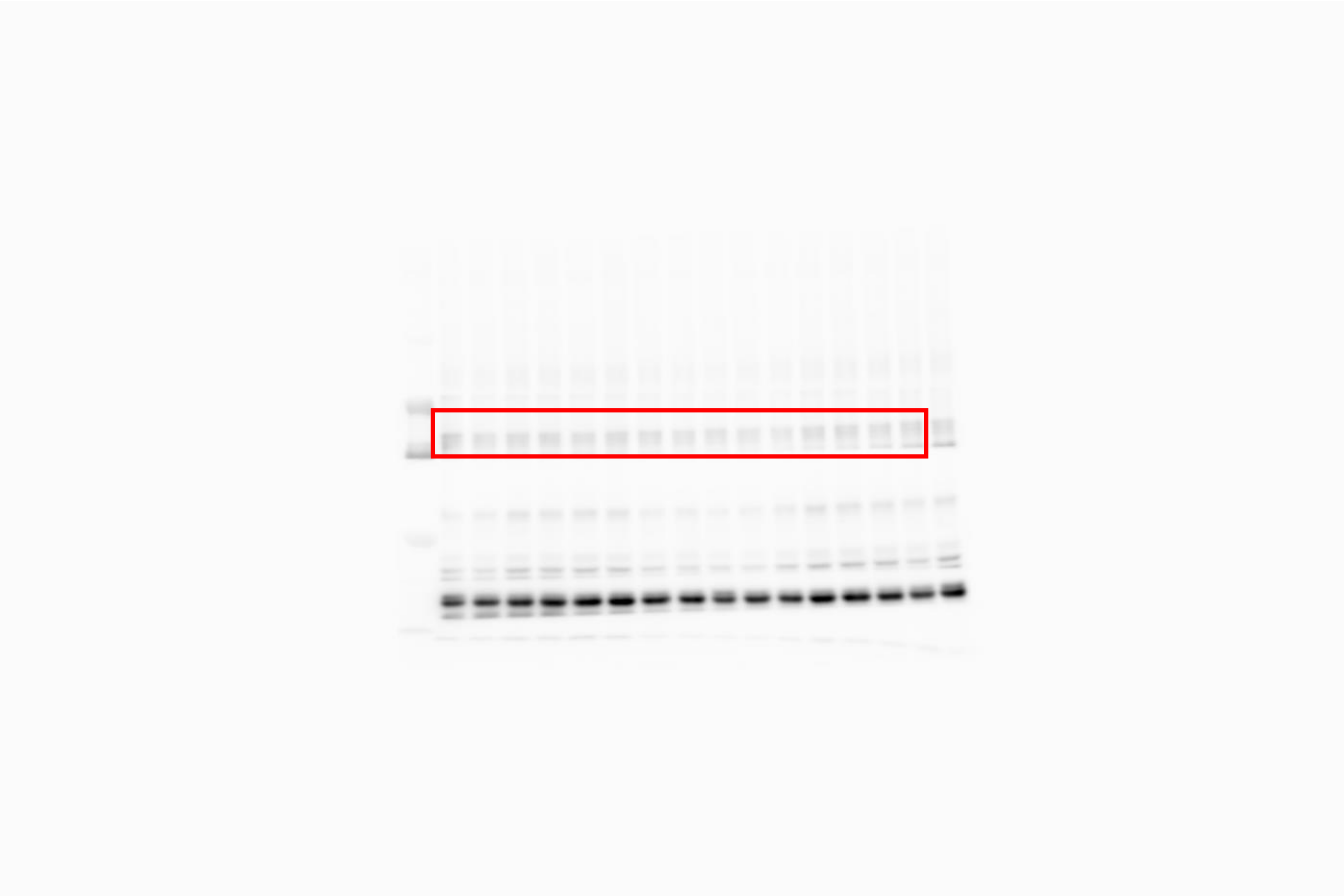

Figure 6A. pFAK (125 kD)

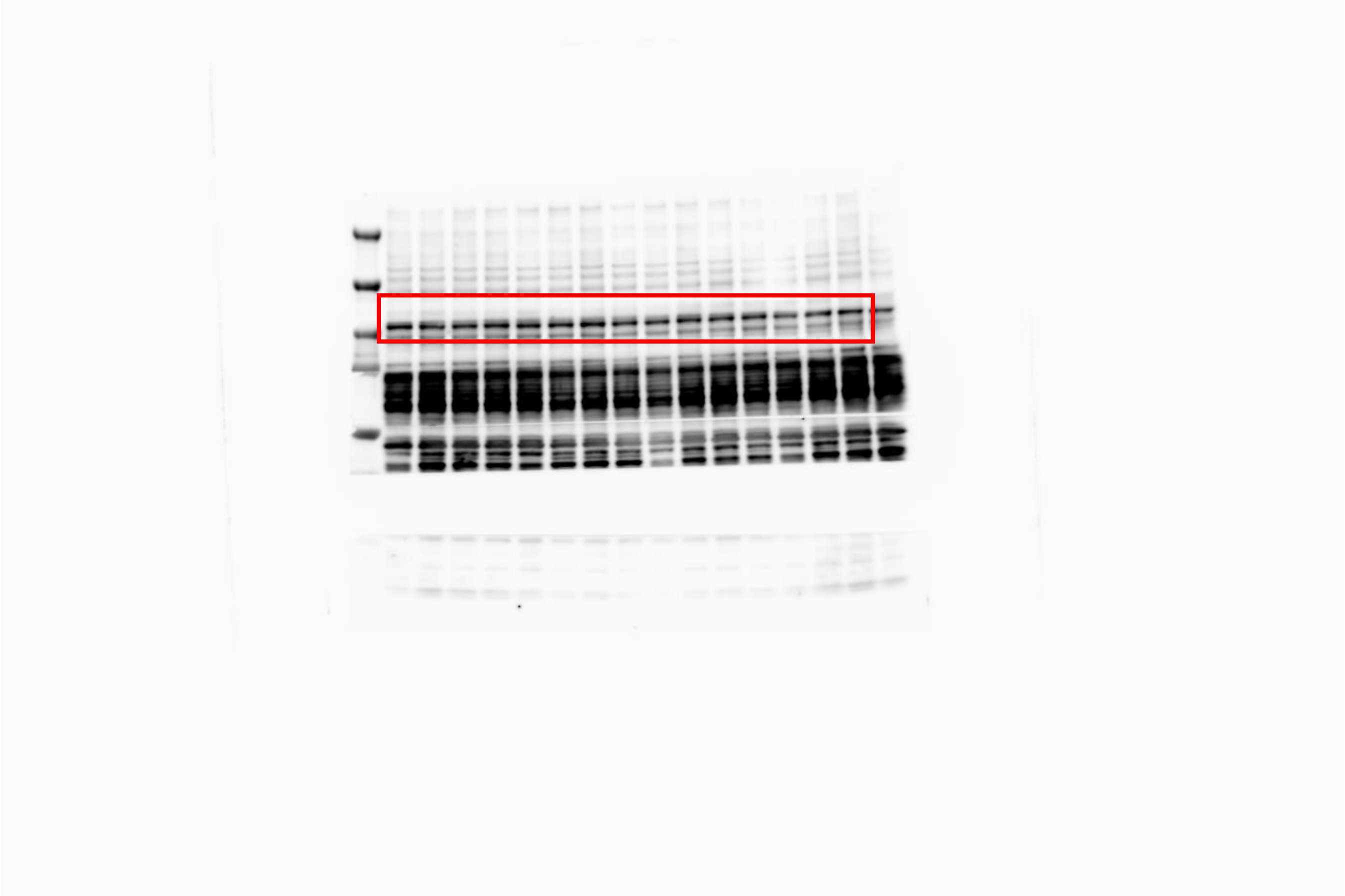

Figure 6A. FAK (125 kD)

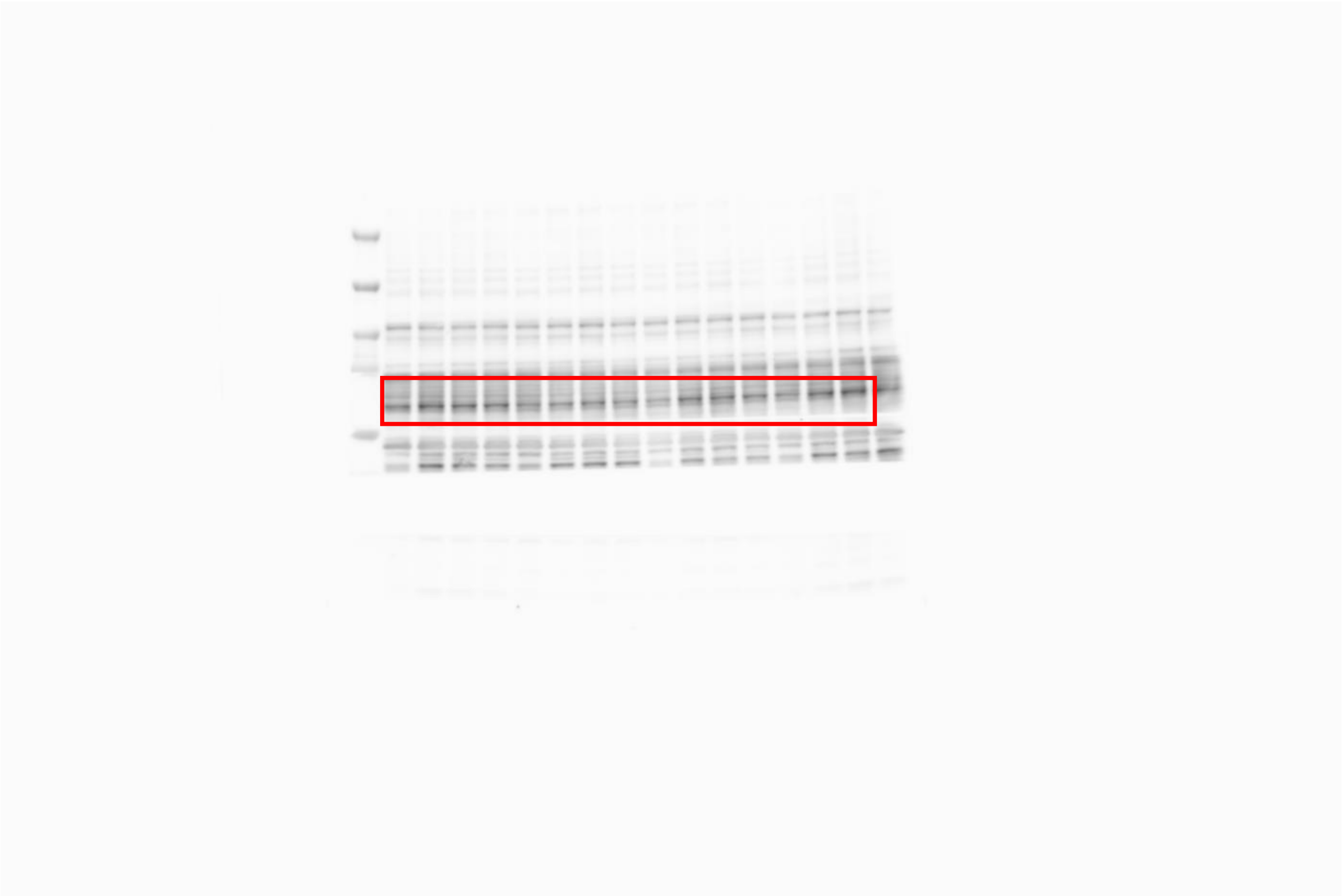

Figure 6A. pAkt (60 kD)

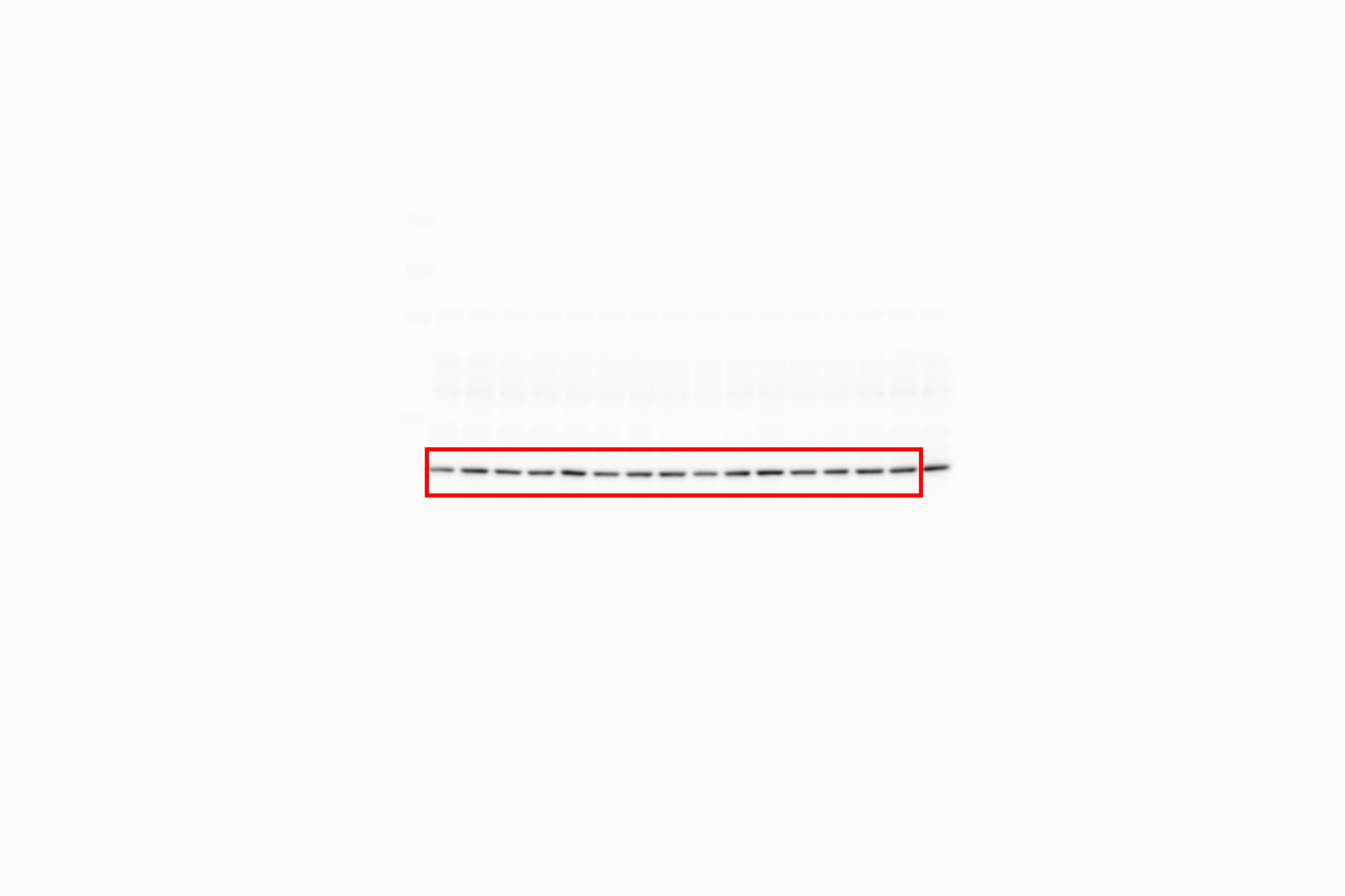

Figure 6A. Akt (60 kD)

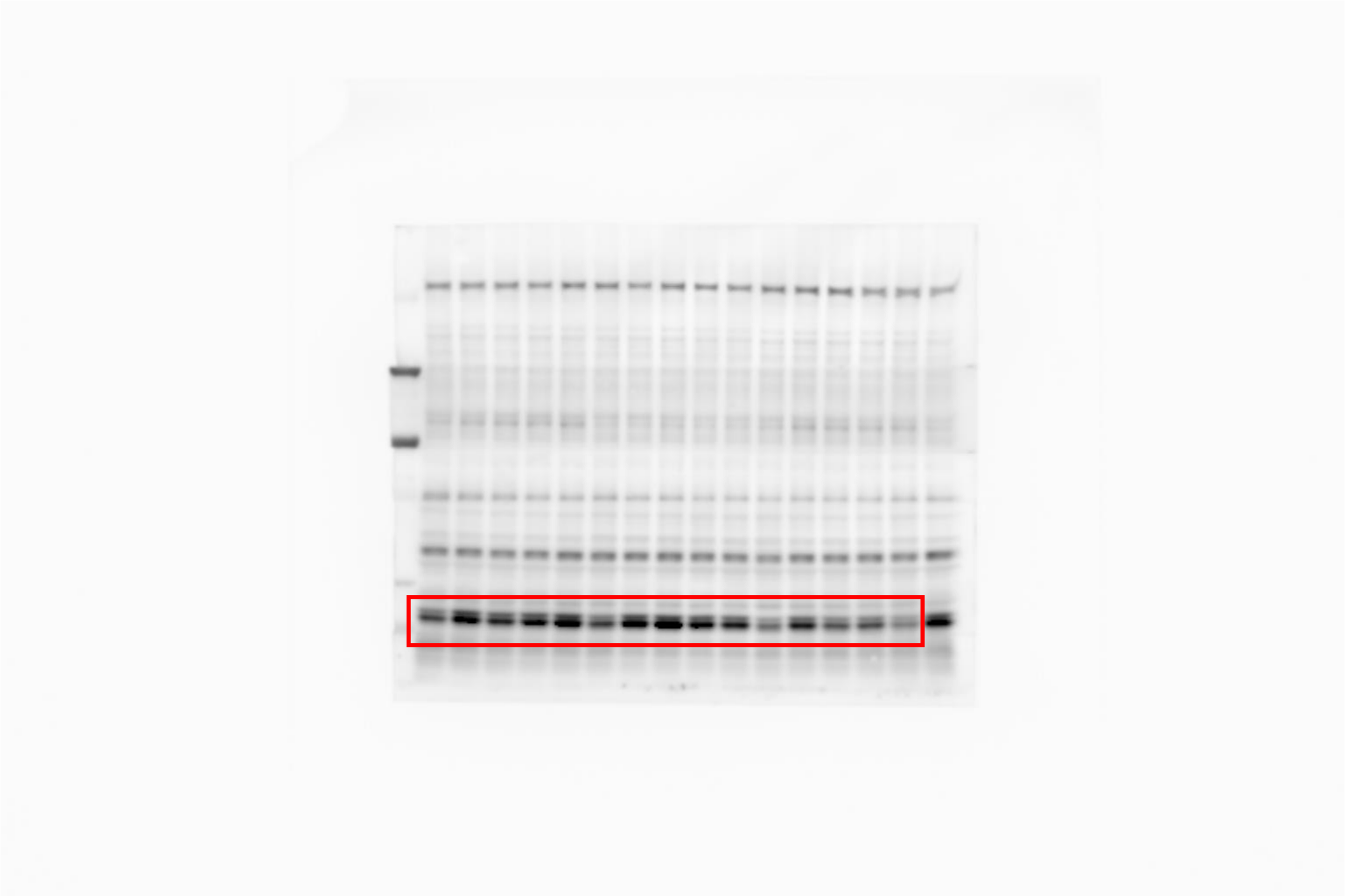

Figure 6A. pERK1/2 (44/42 kD)

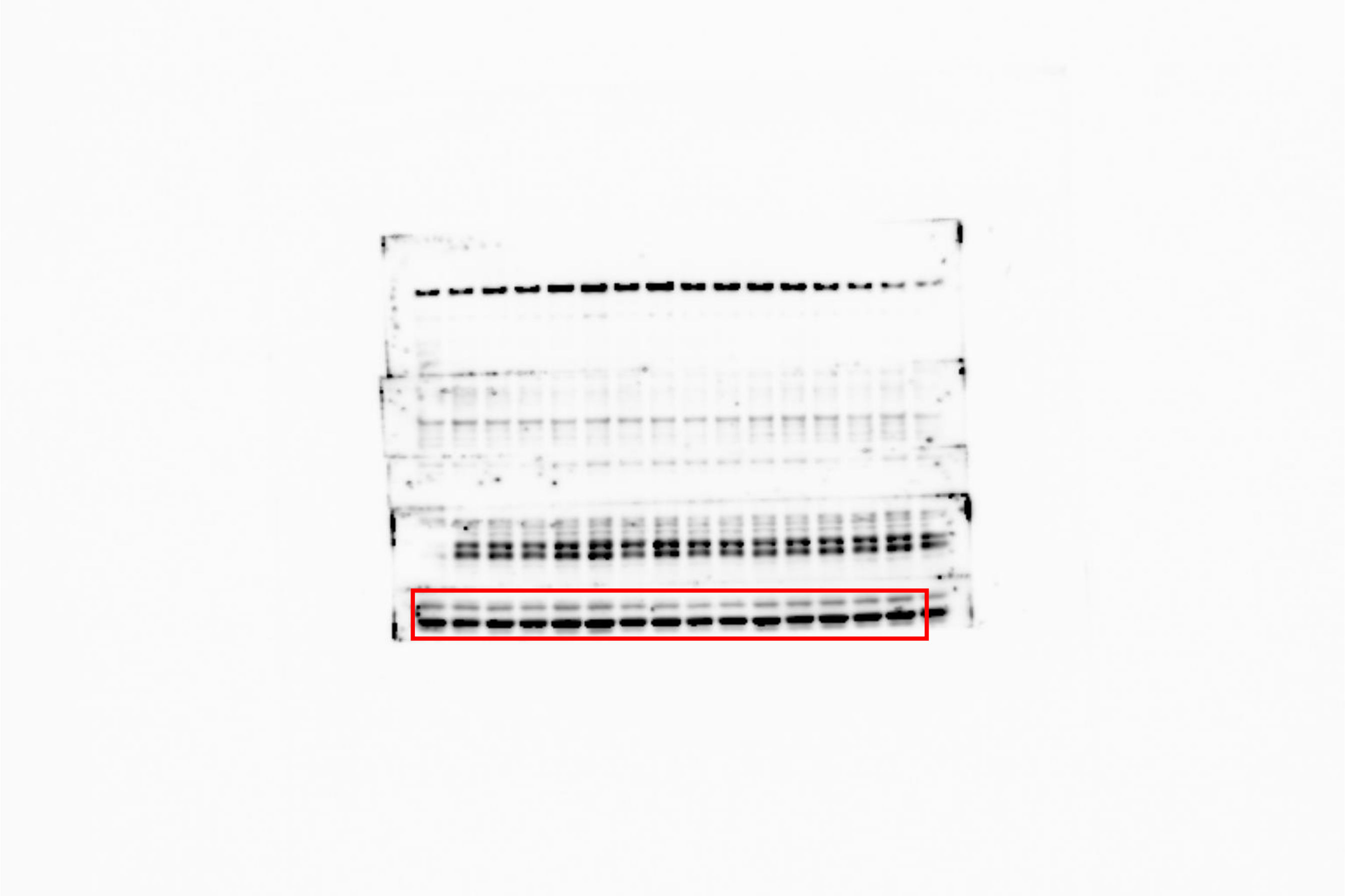

Figure 6A. ERK1/2 (44/42 kD)

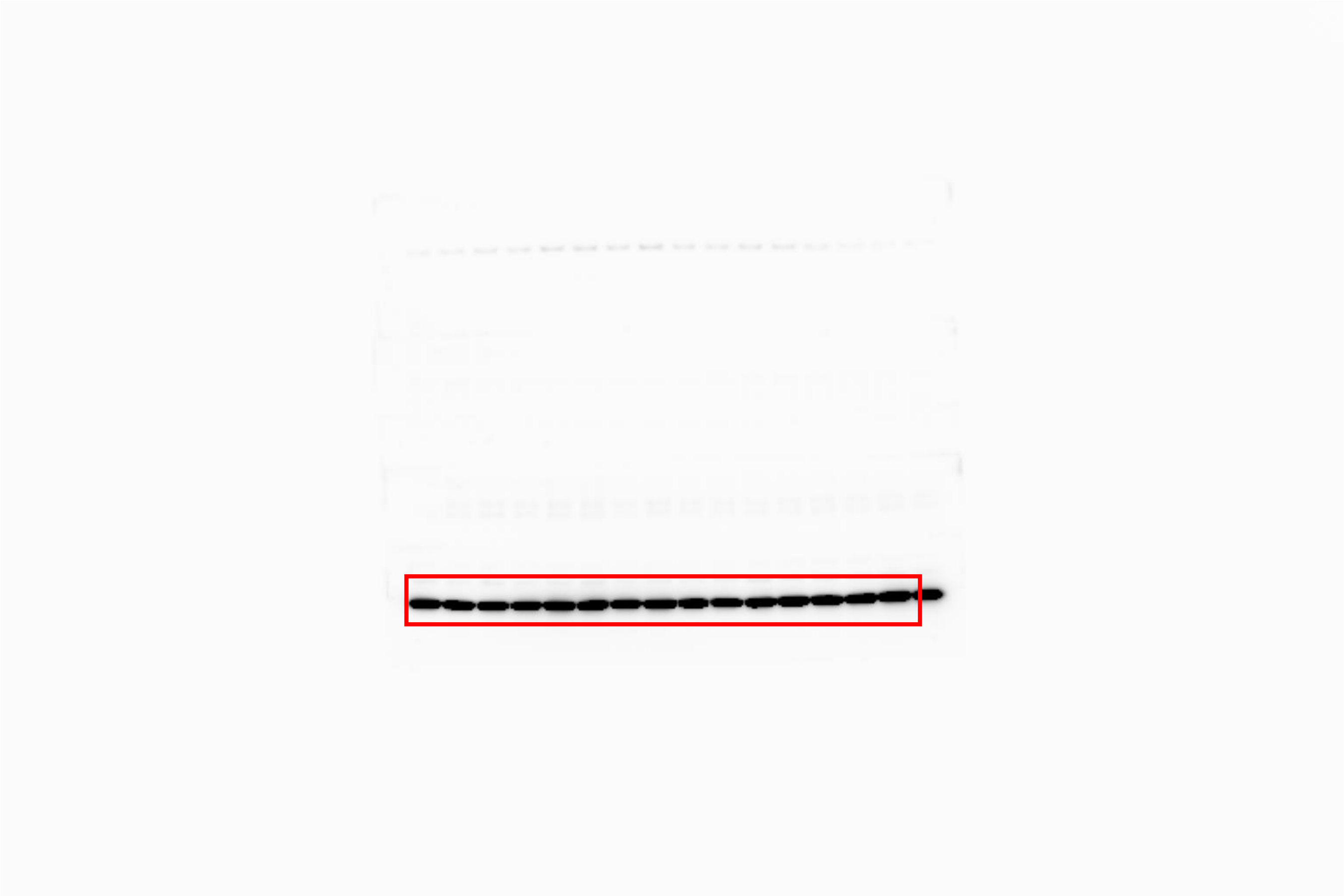

Figure 6A. GAPDH (36 kD)

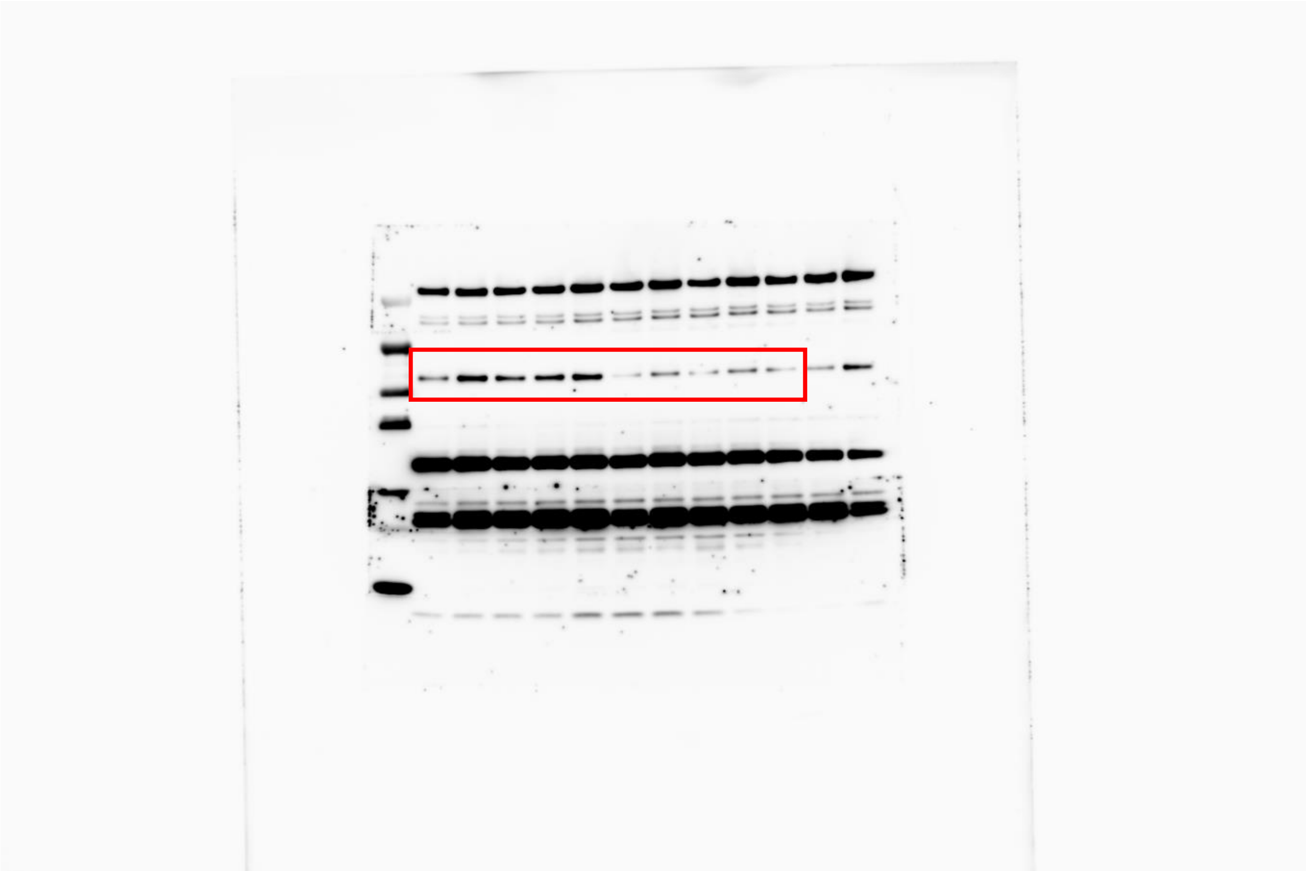

Figure 6F. pFAK (125 kD)

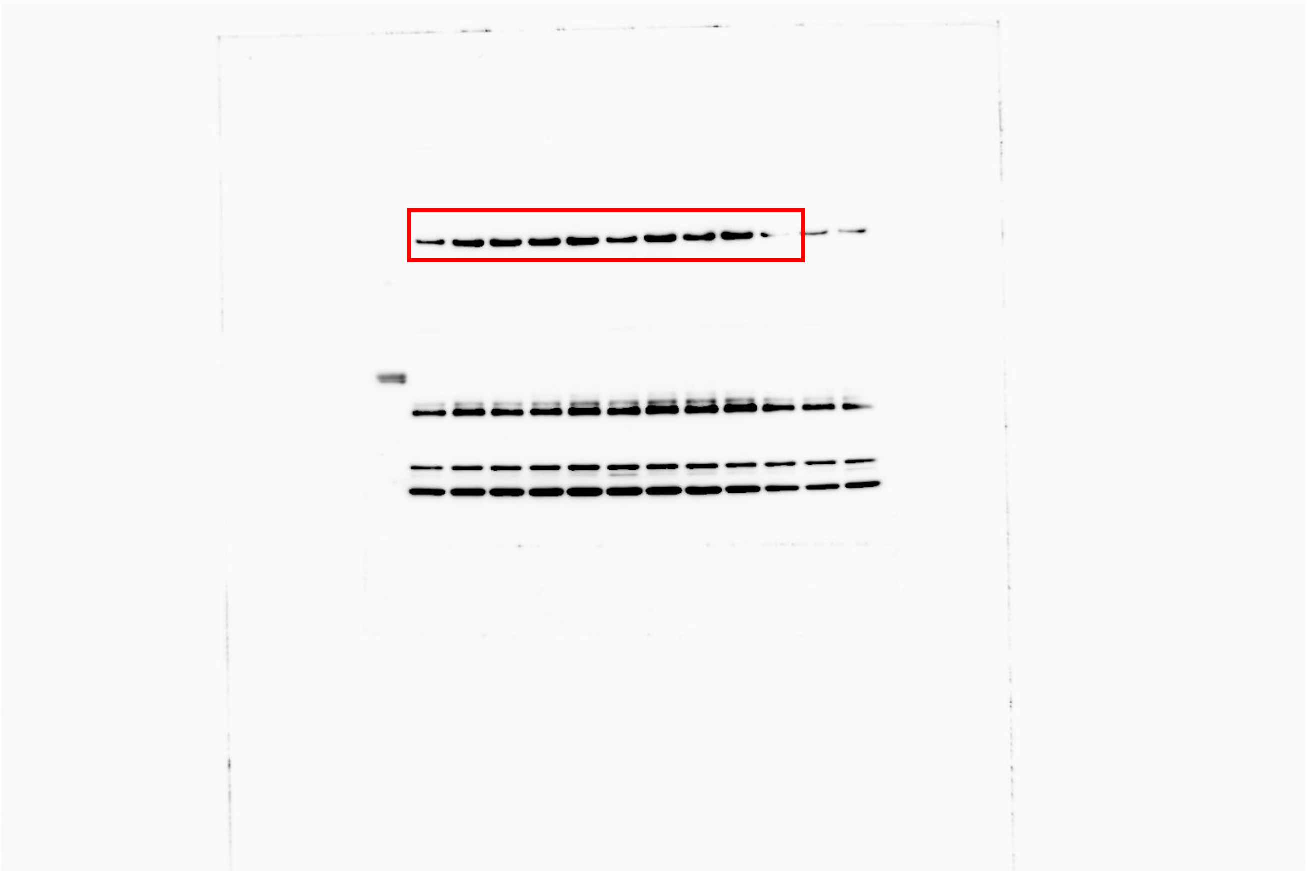

Figure 6F. FAK (125 kD)

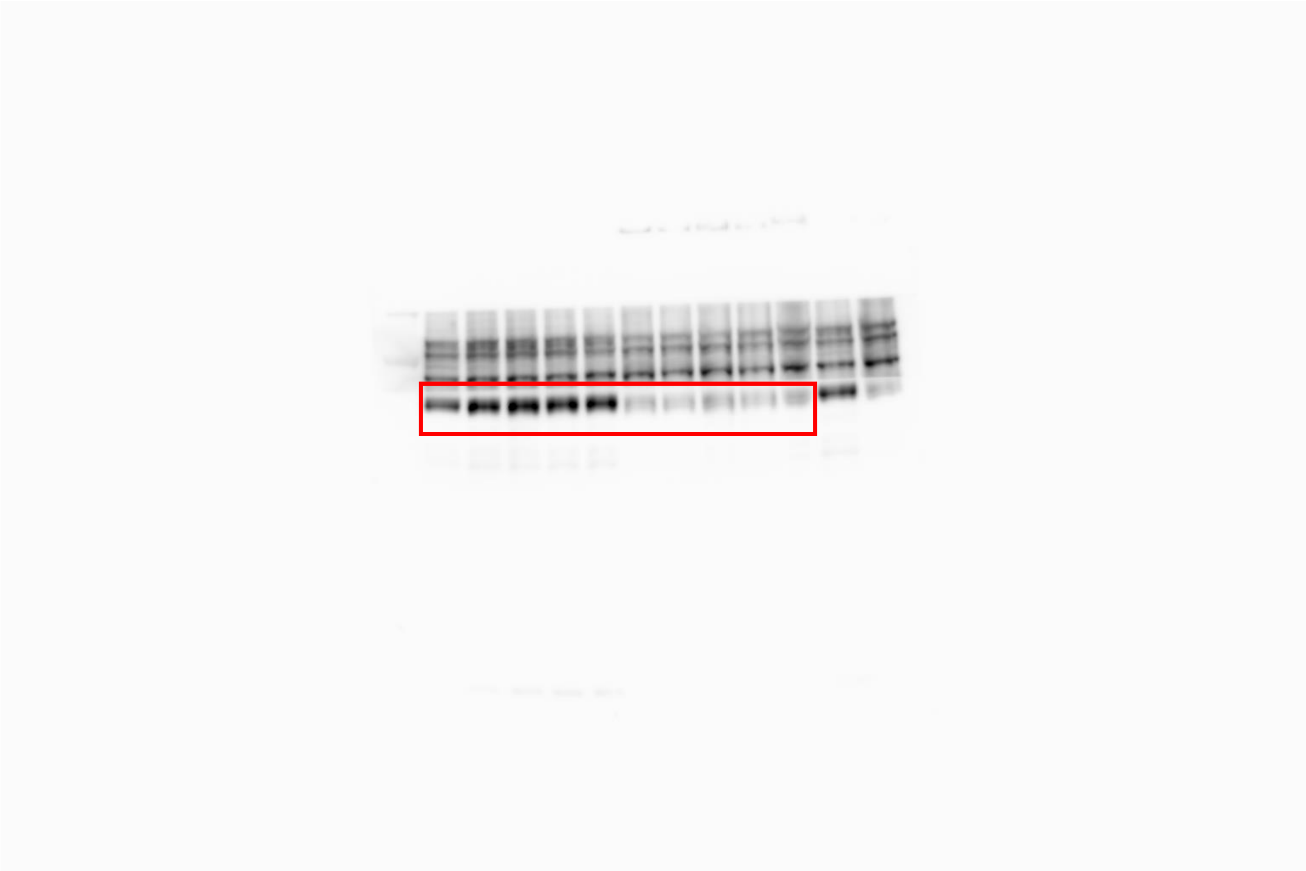

Figure 6F. pFAK (125 kD)

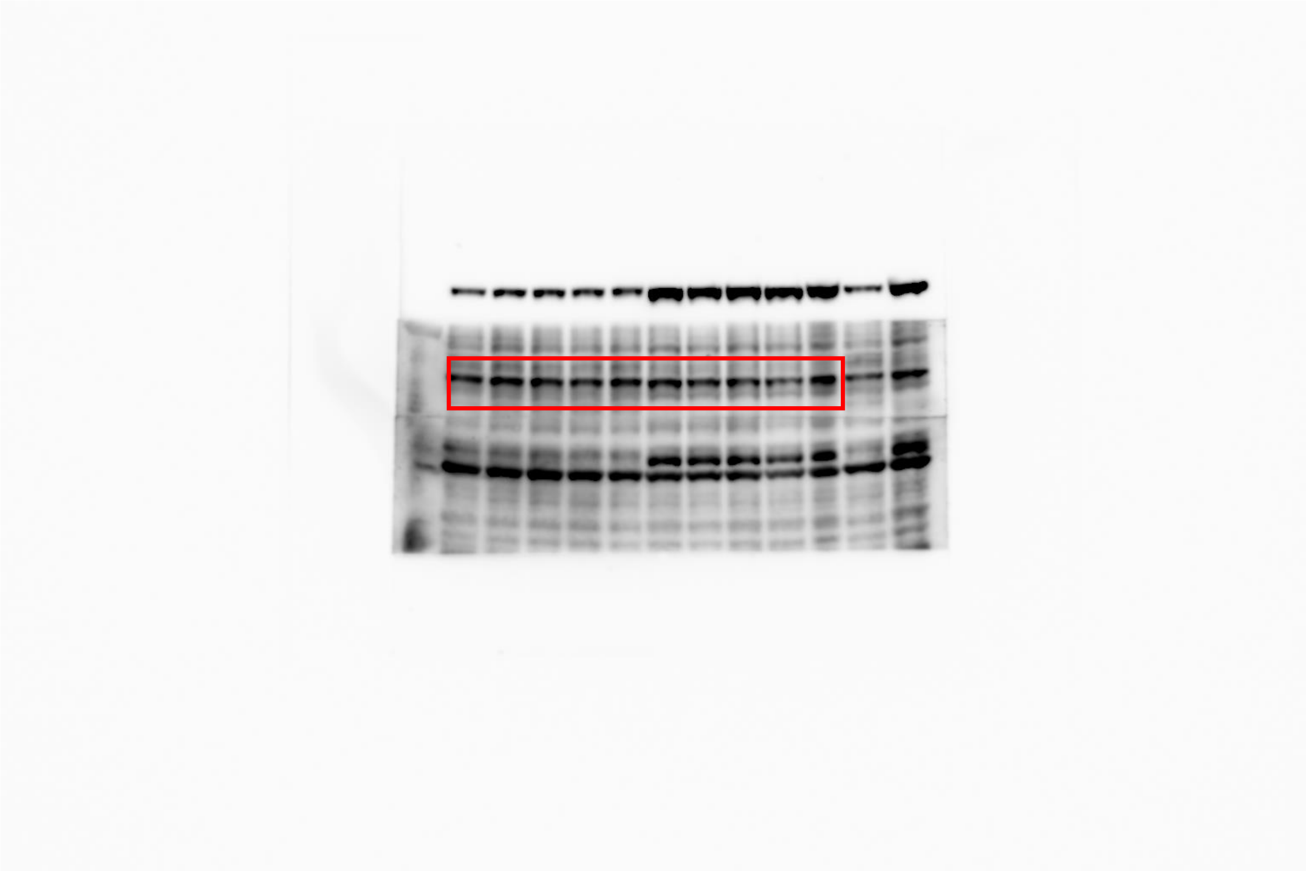

Figure 6F. FAK (125 kD)

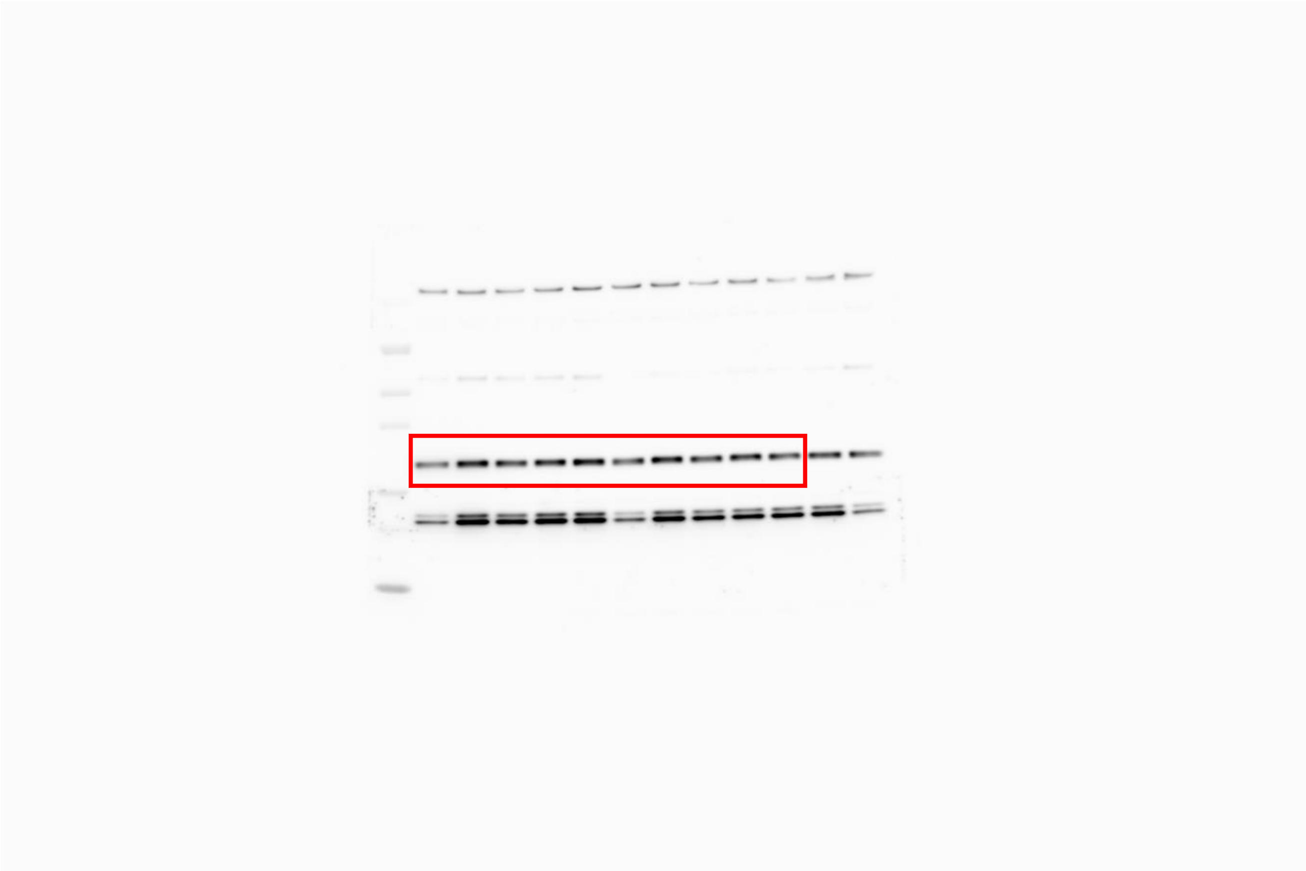

Figure 6F. pAkt (60 kD)

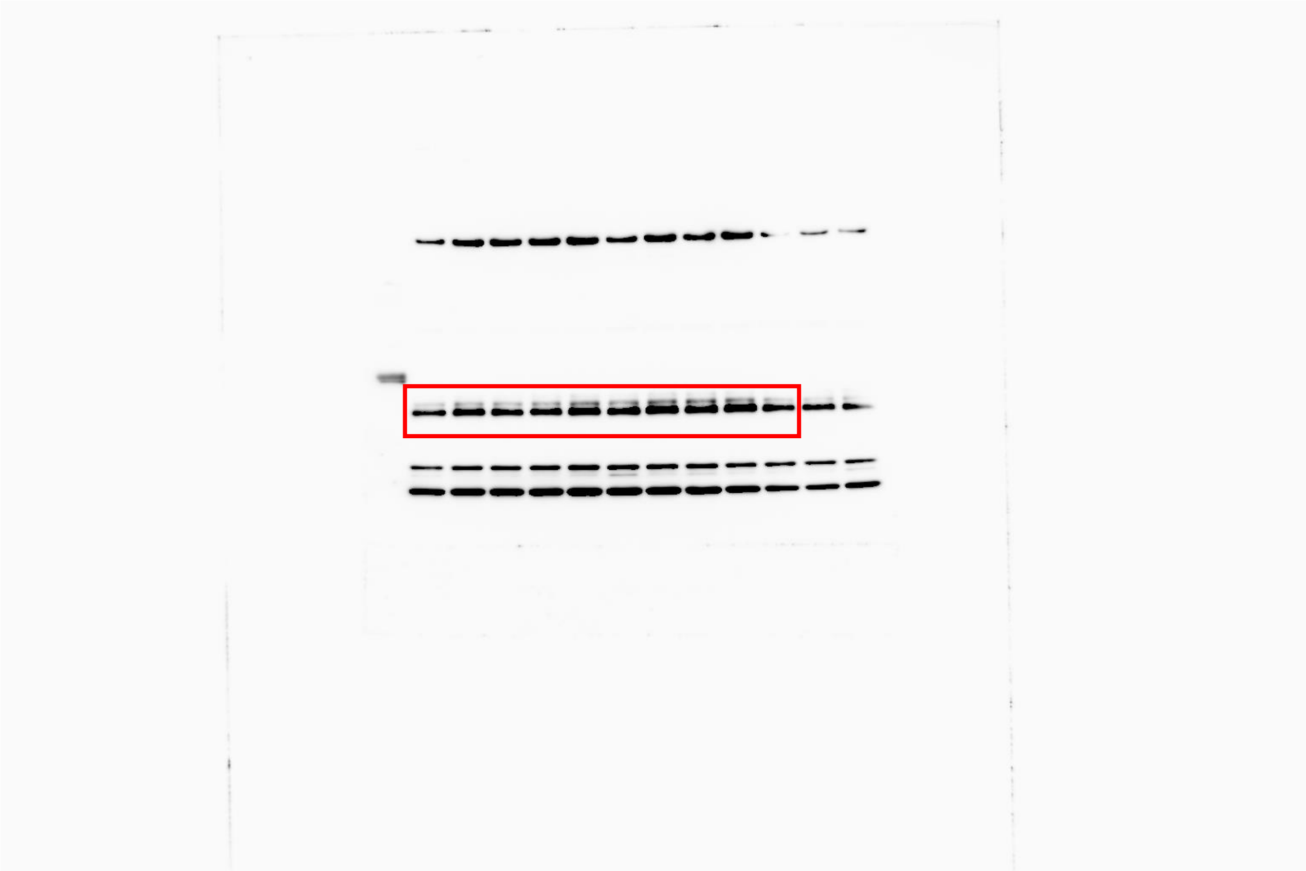

Figure 6F. Akt (60 kD)

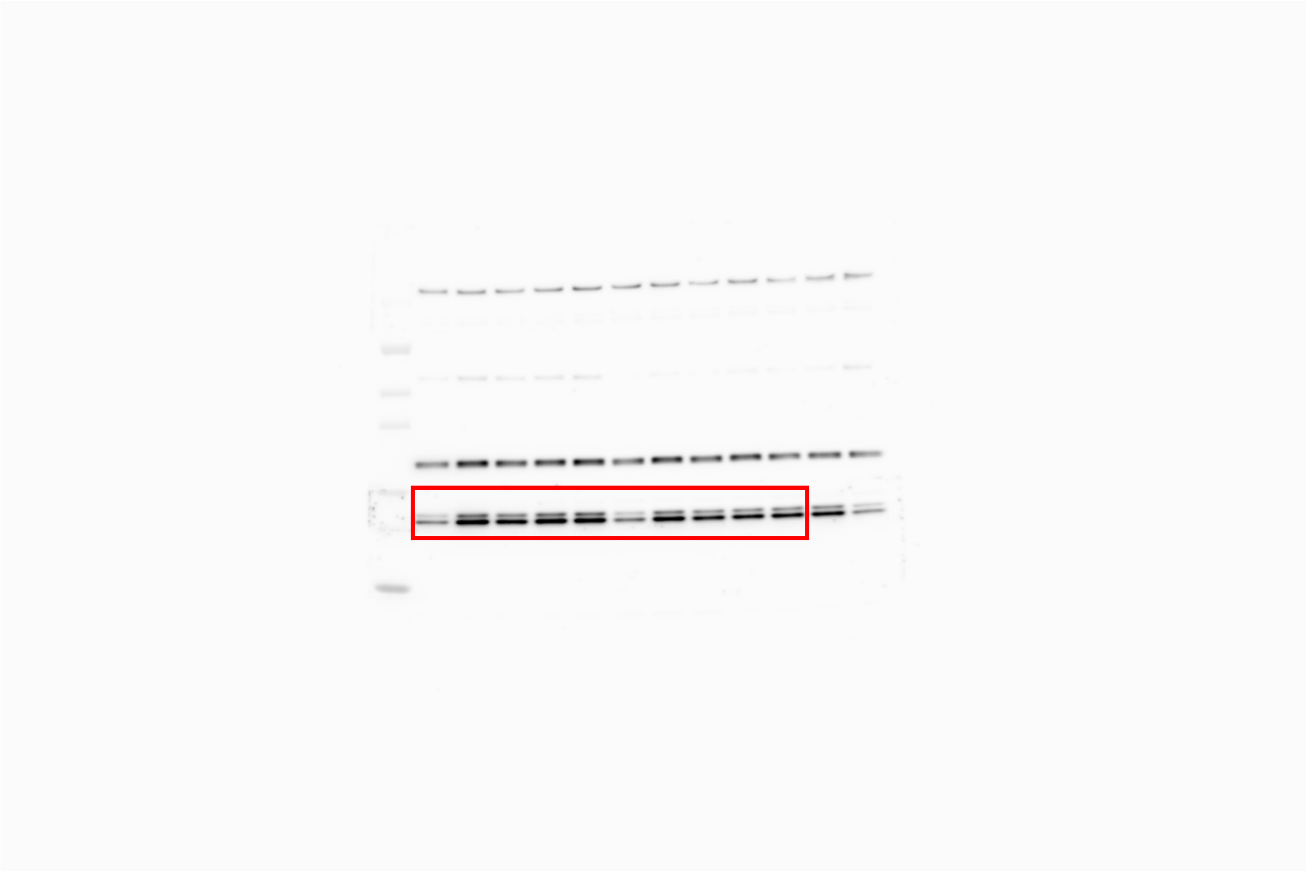

Figure 6F. pERK1/2 (44/42 kD)

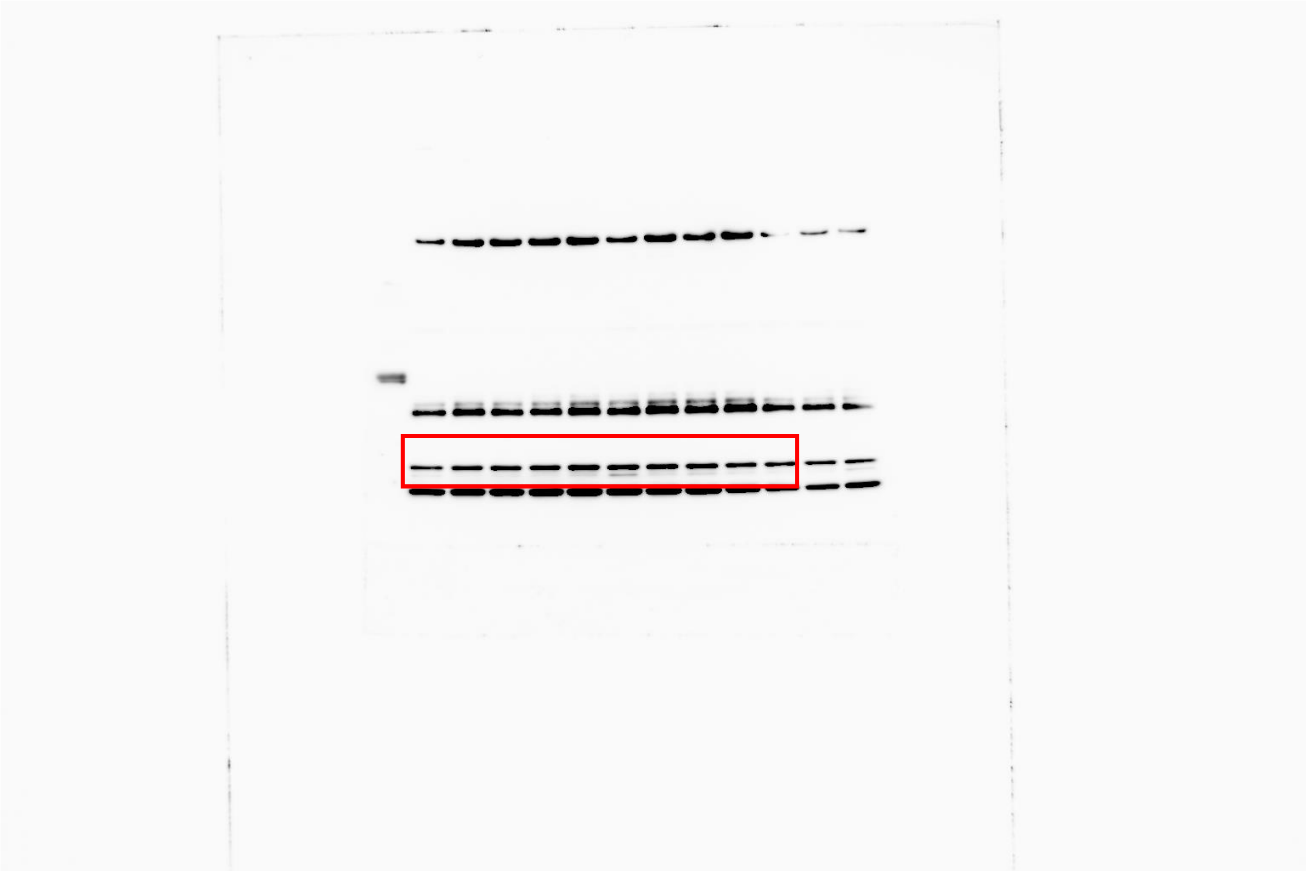

Figure 6F. ERK1/2 (44/42 kD)

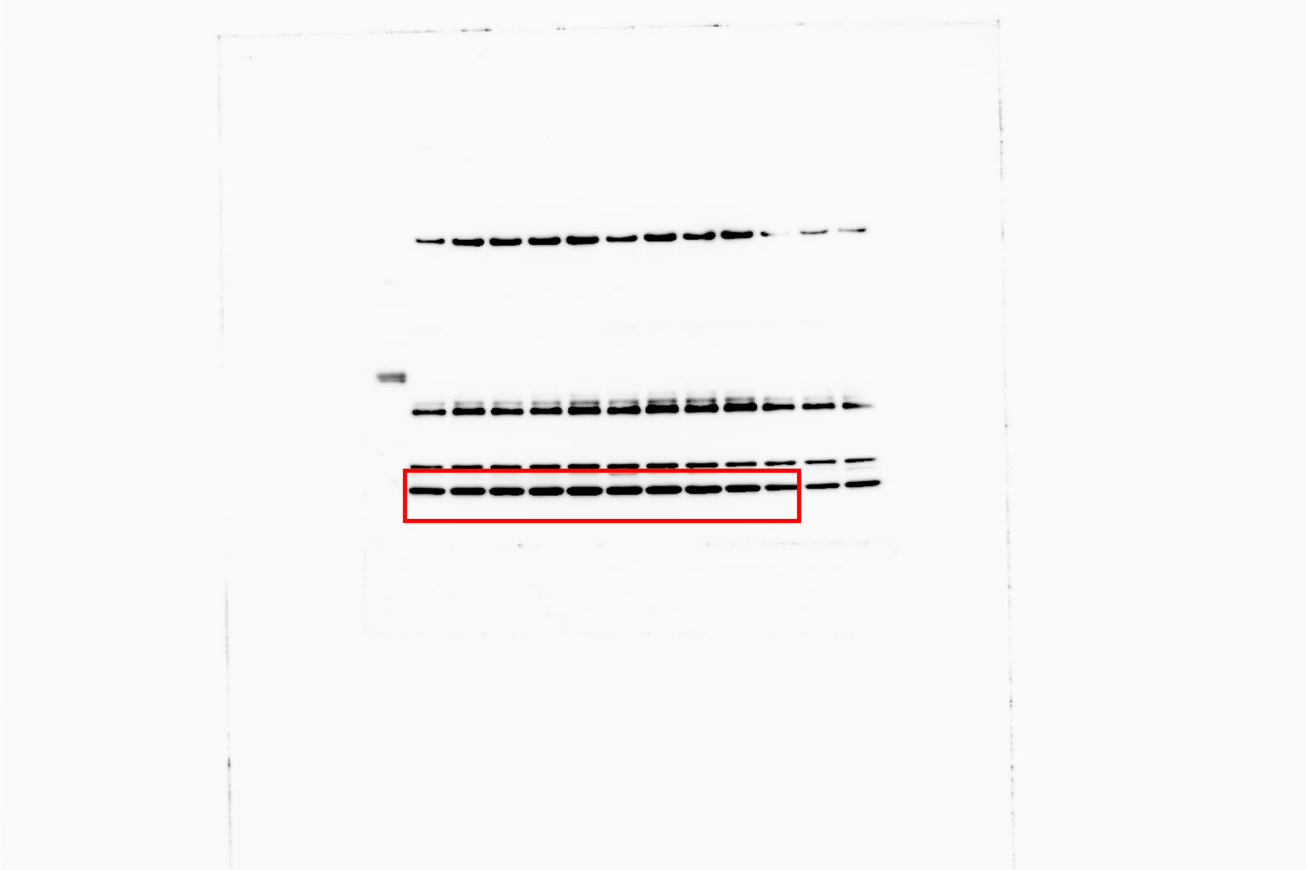

Figure 6F. GAPDH (36 kD)

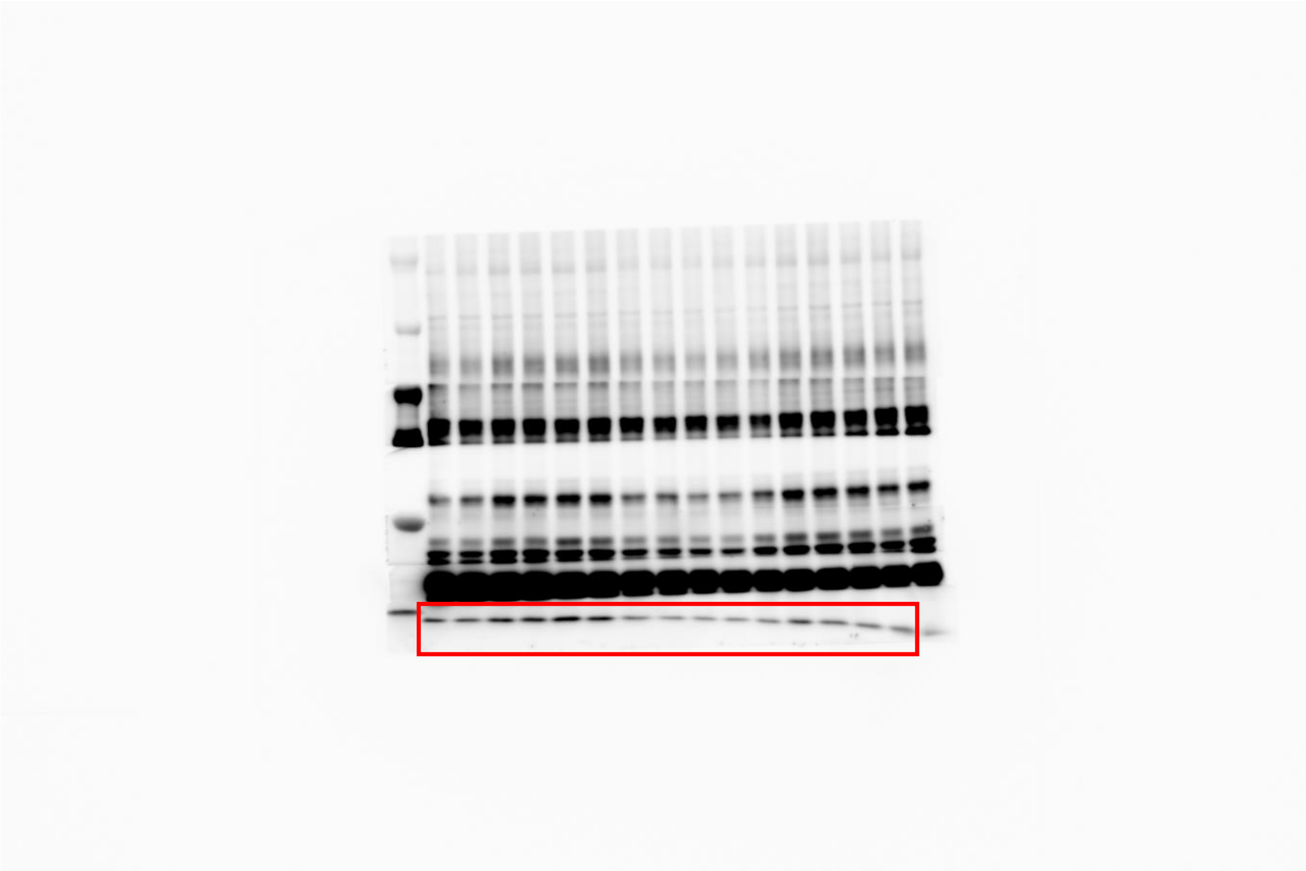

Figure 7A. pRac/Cdc42 (28 kD)

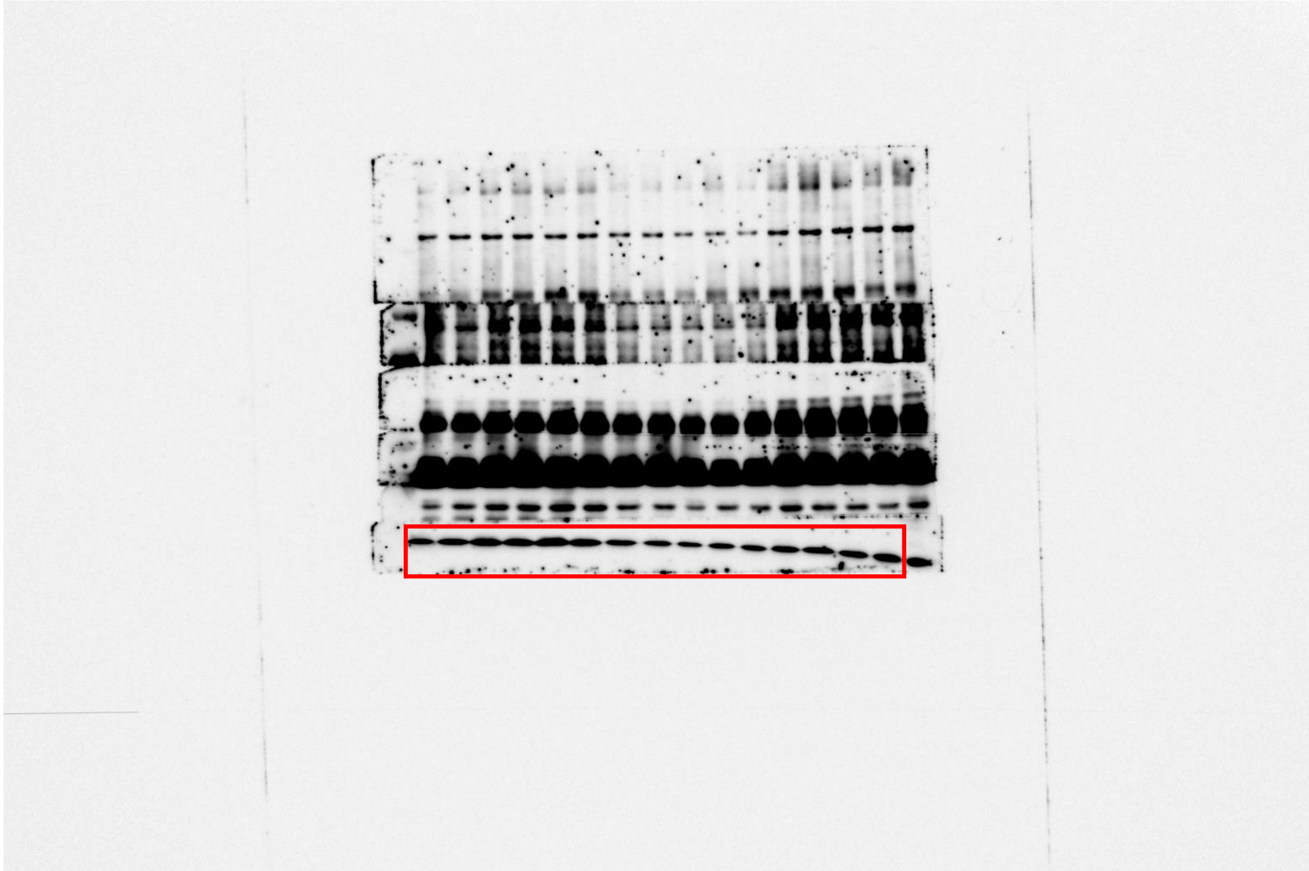

Figure 7A. Rac/Cdc42 (28 kD)

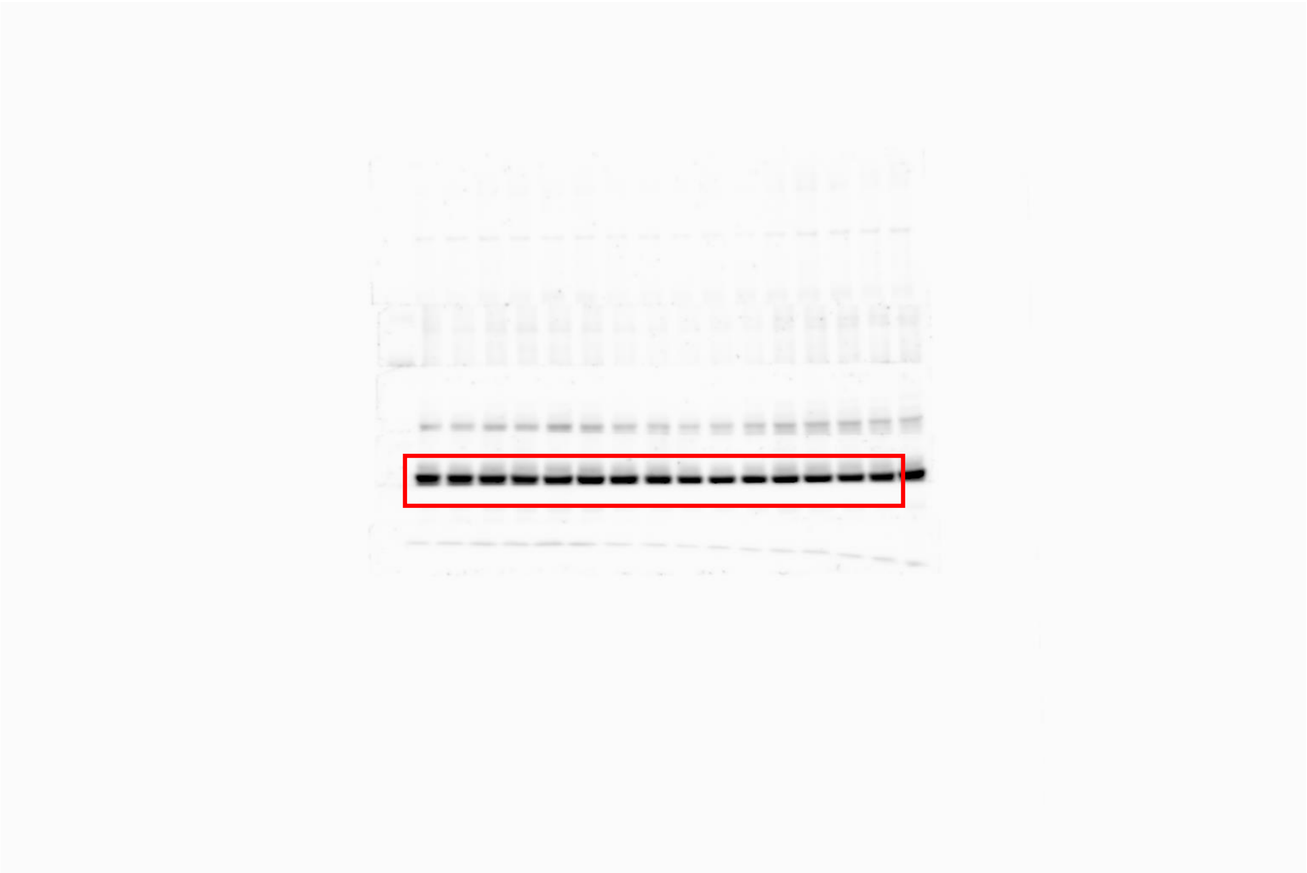

Figure 7A. GAPDH (36 kD)

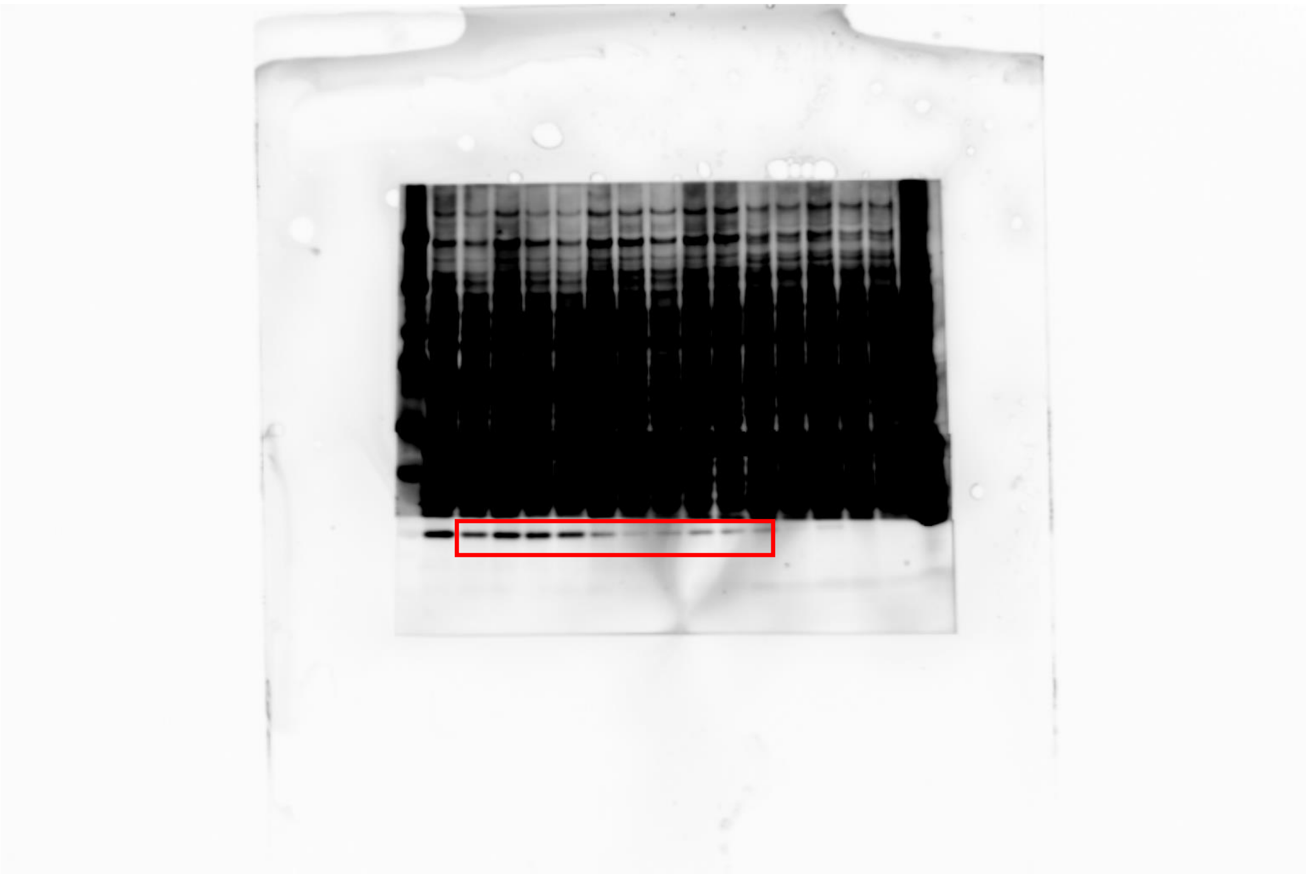

Figure 7C. pRac/Cdc42 (28 kD)

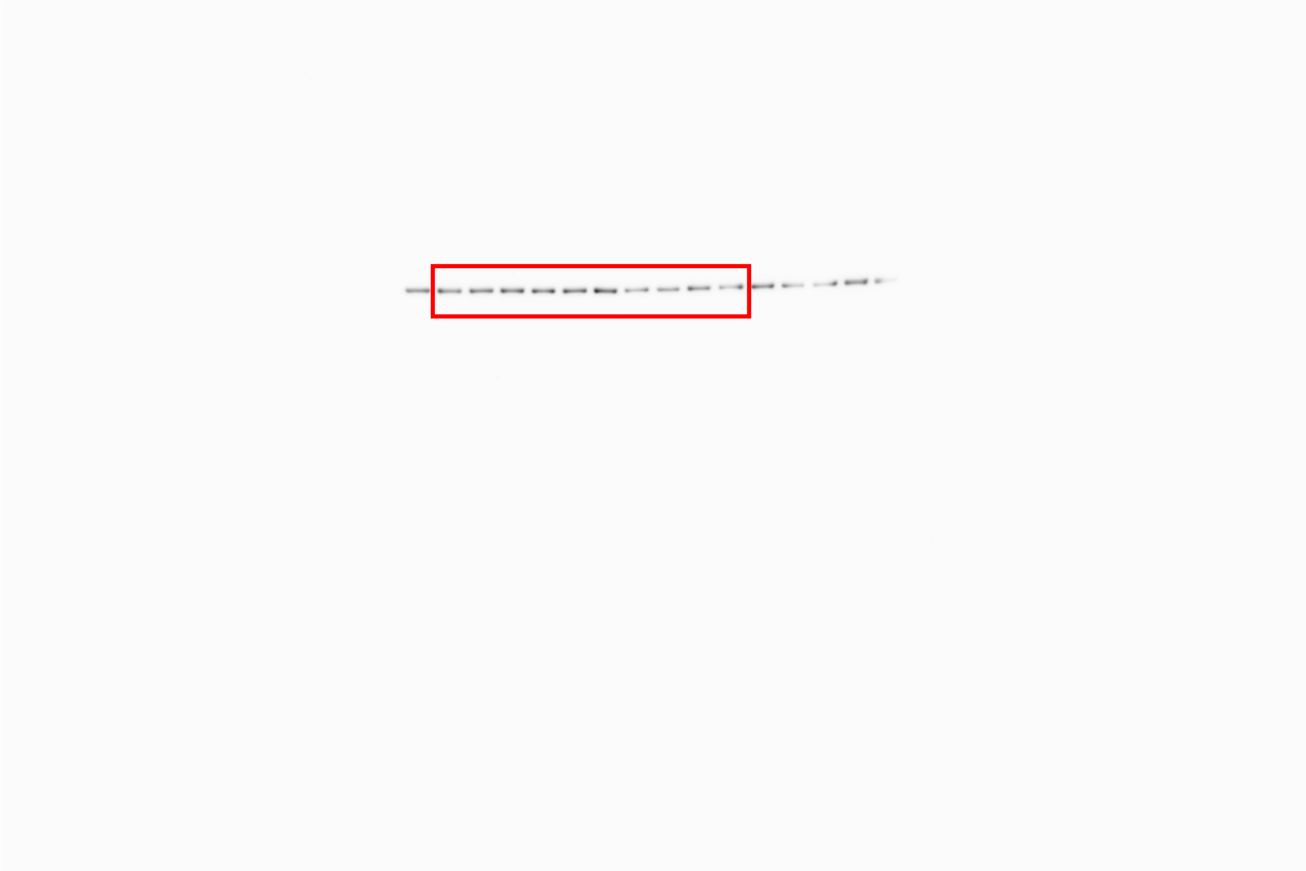

Figure 7C. Rac/Cdc42 (28 kD)

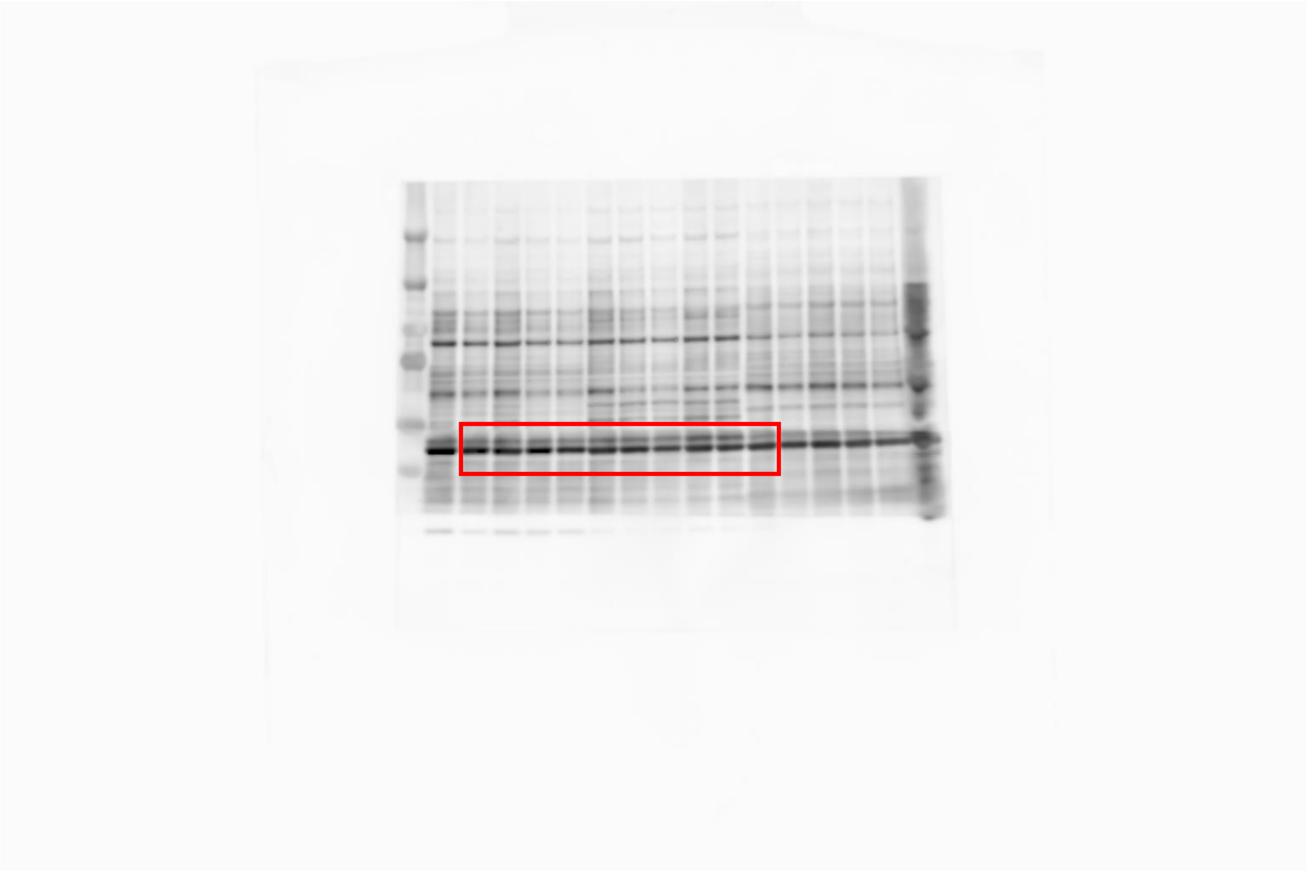

Figure 7C. GAPDH (36 kD)

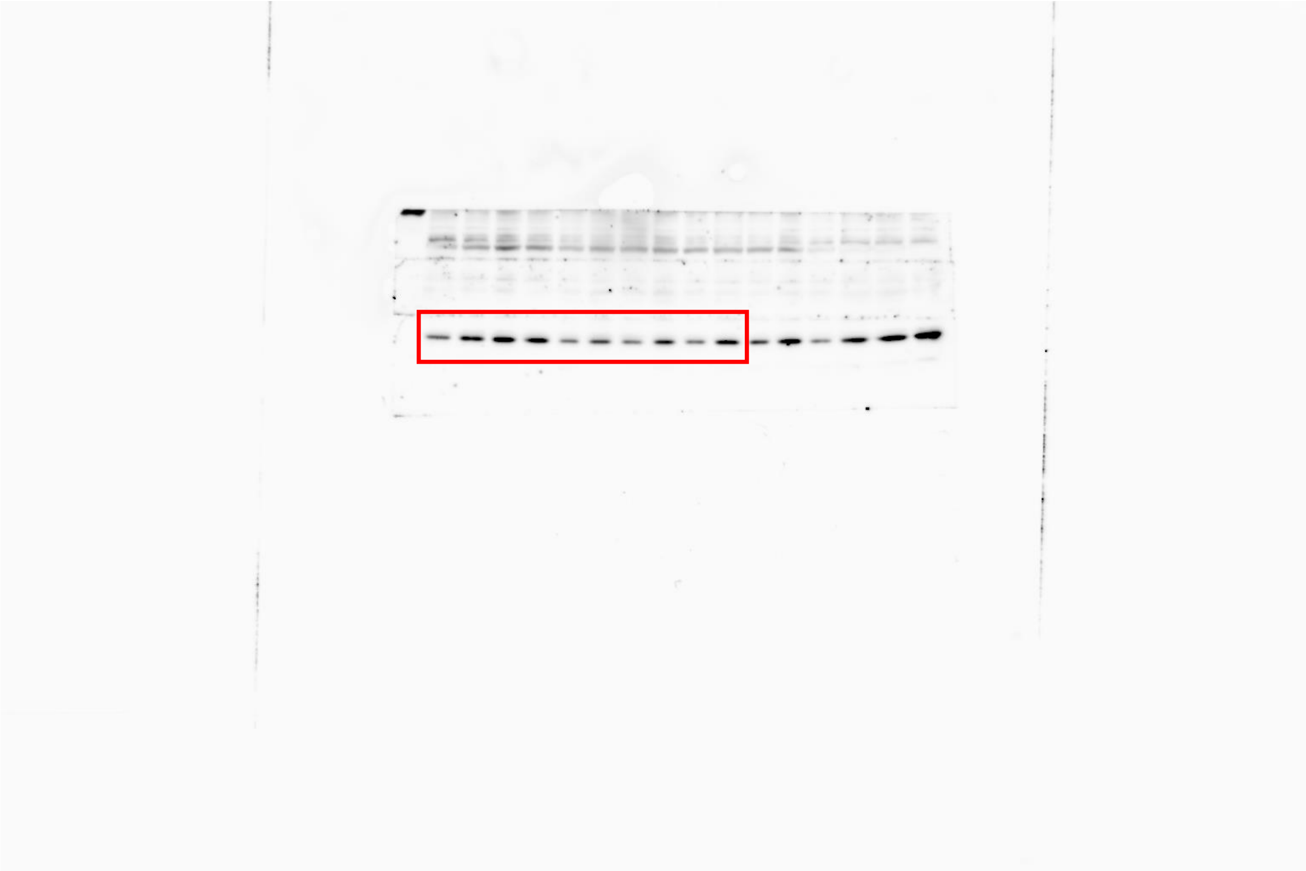

Figure 7E. pRac/Cdc42 (28 kD)

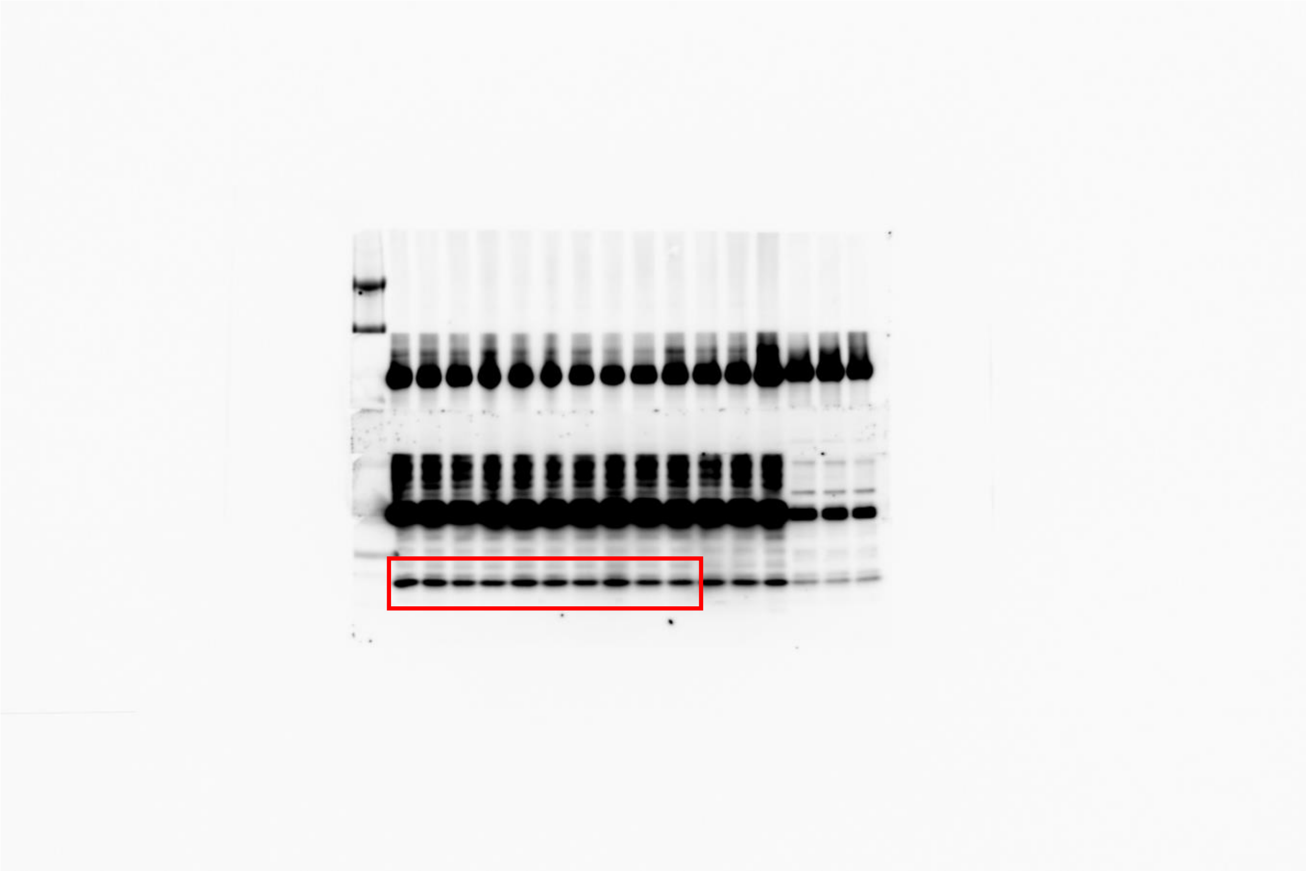

Figure 7E. Rac/Cdc42 (28 kD)

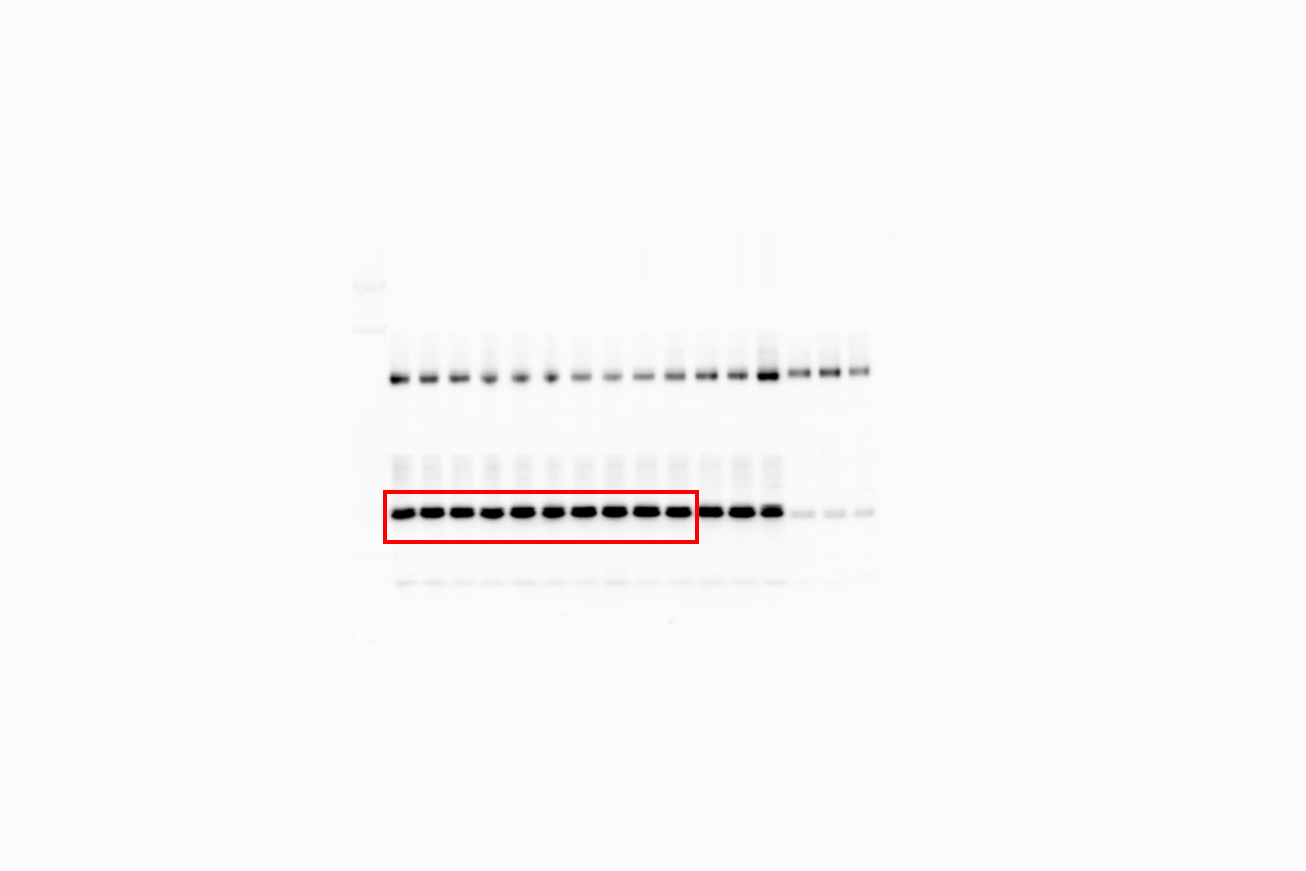

Figure 7E. GAPDH (36 kD)

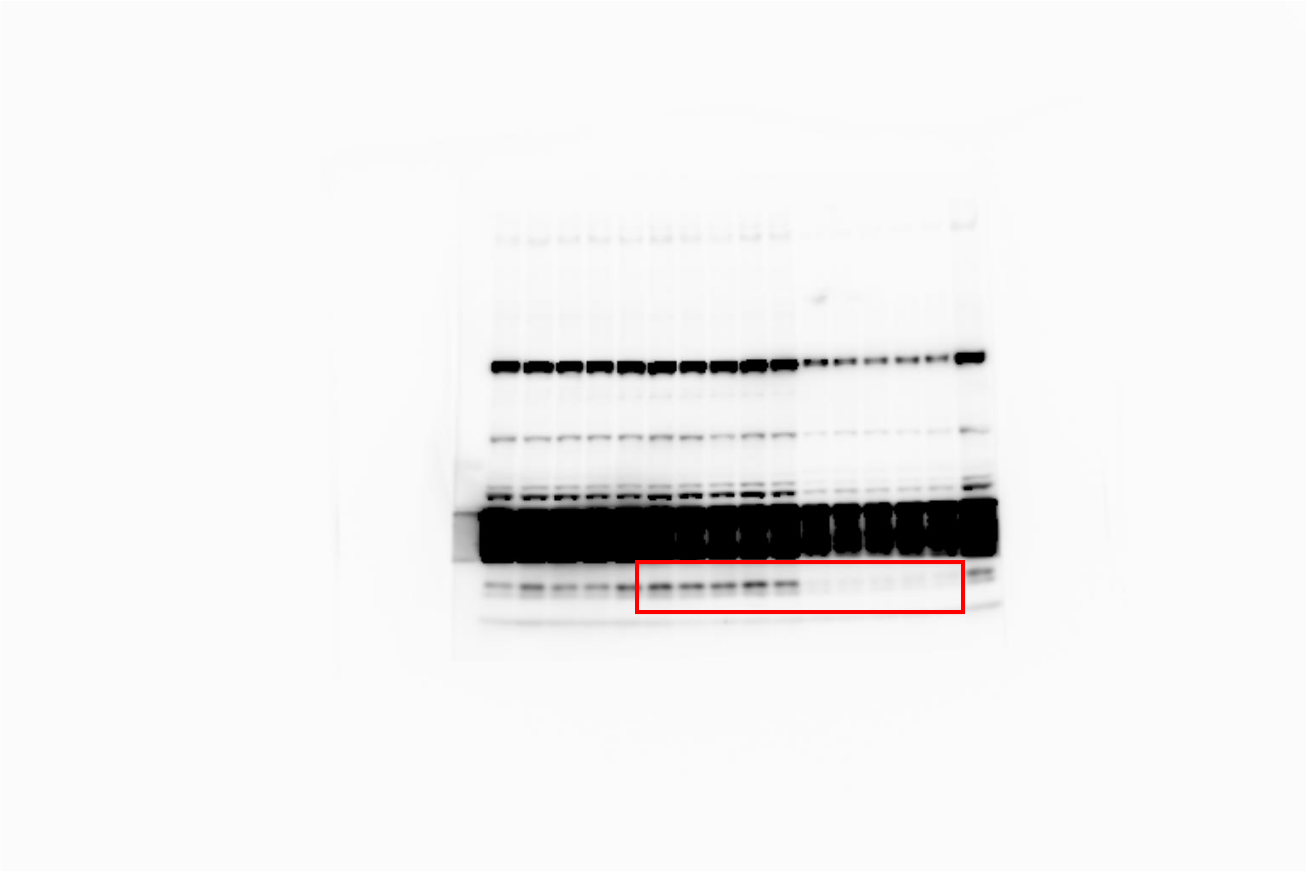

Figure 7G. pRac/Cdc42 (28 kD)

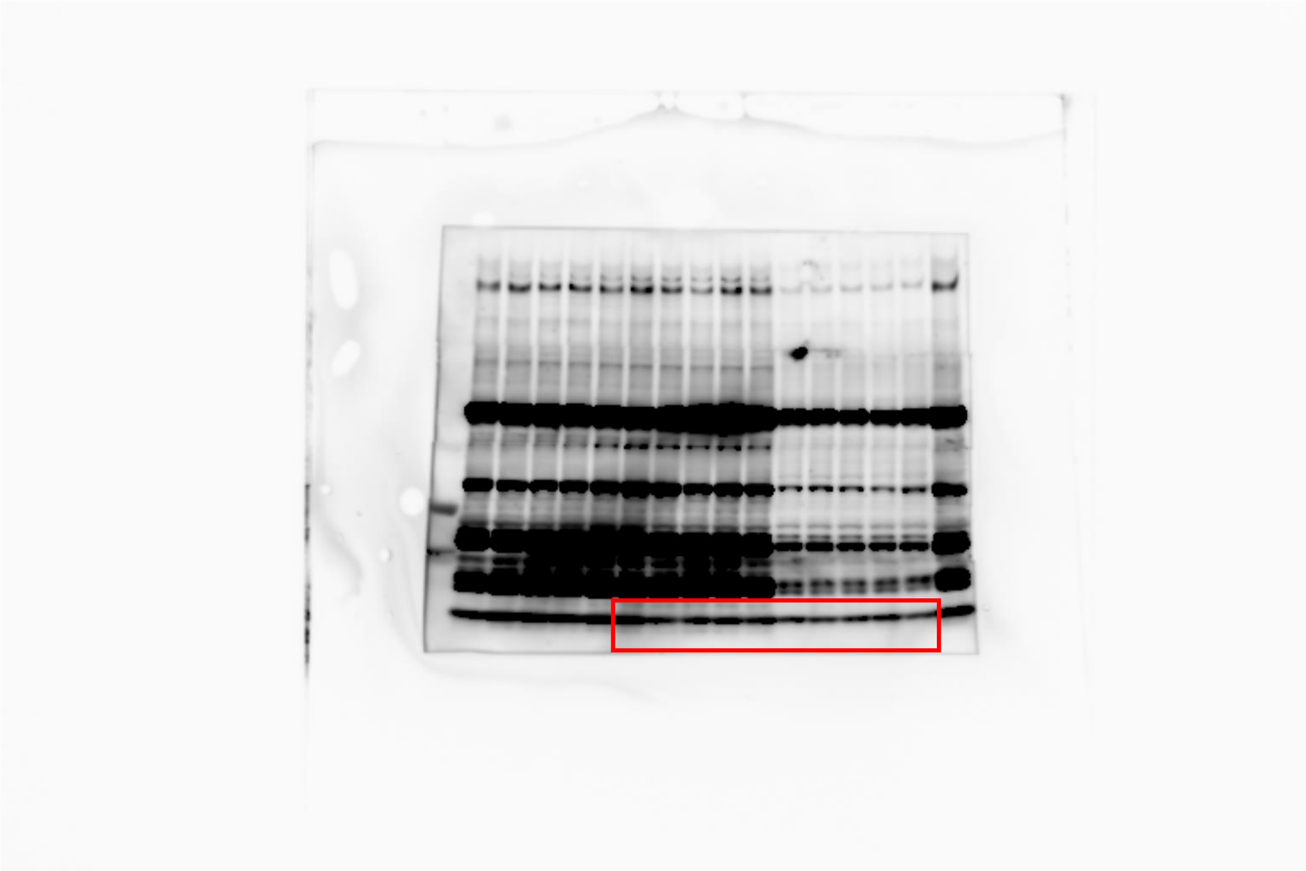

Figure 7G. Rac/Cdc42 (28 kD)

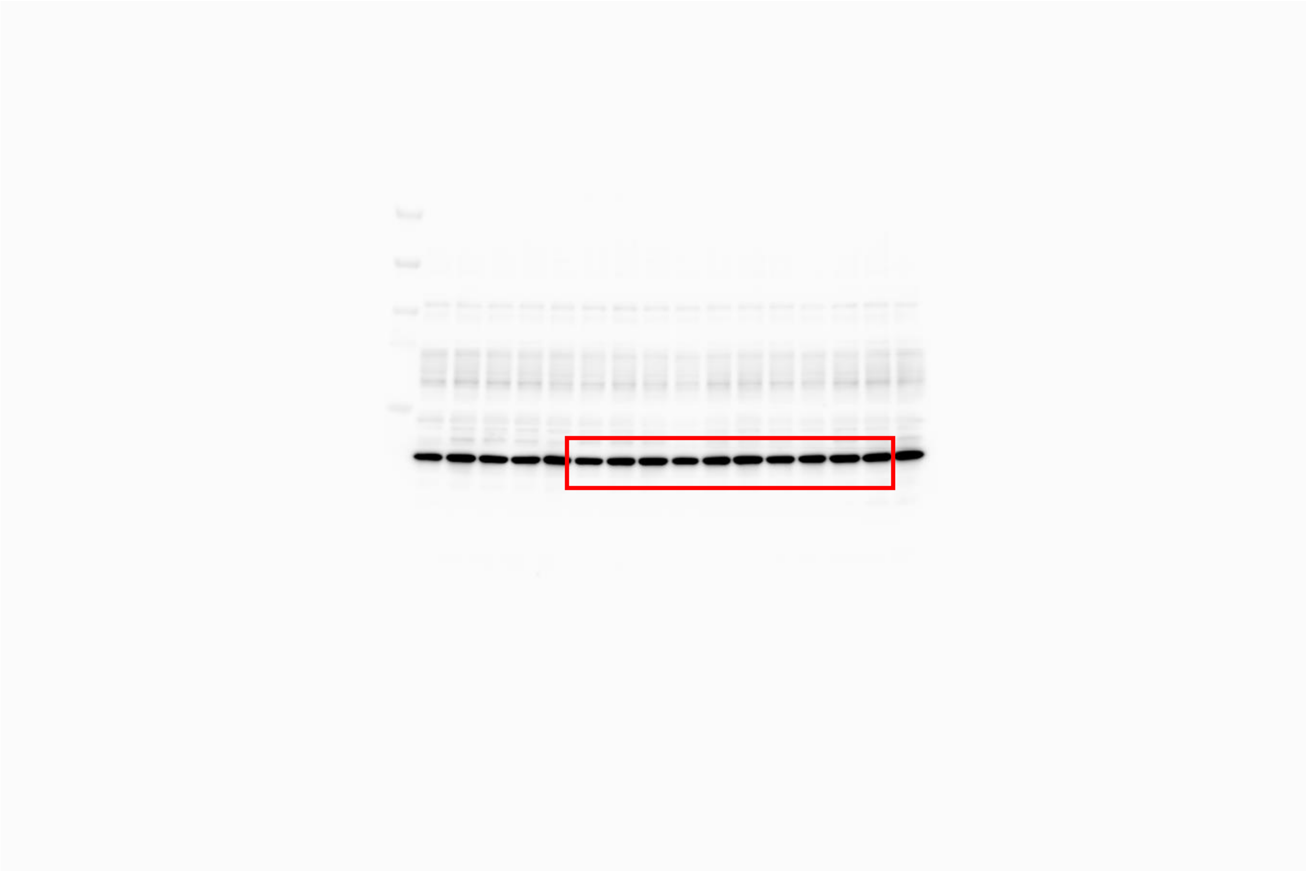

Figure 7G. GAPDH (36 kD)

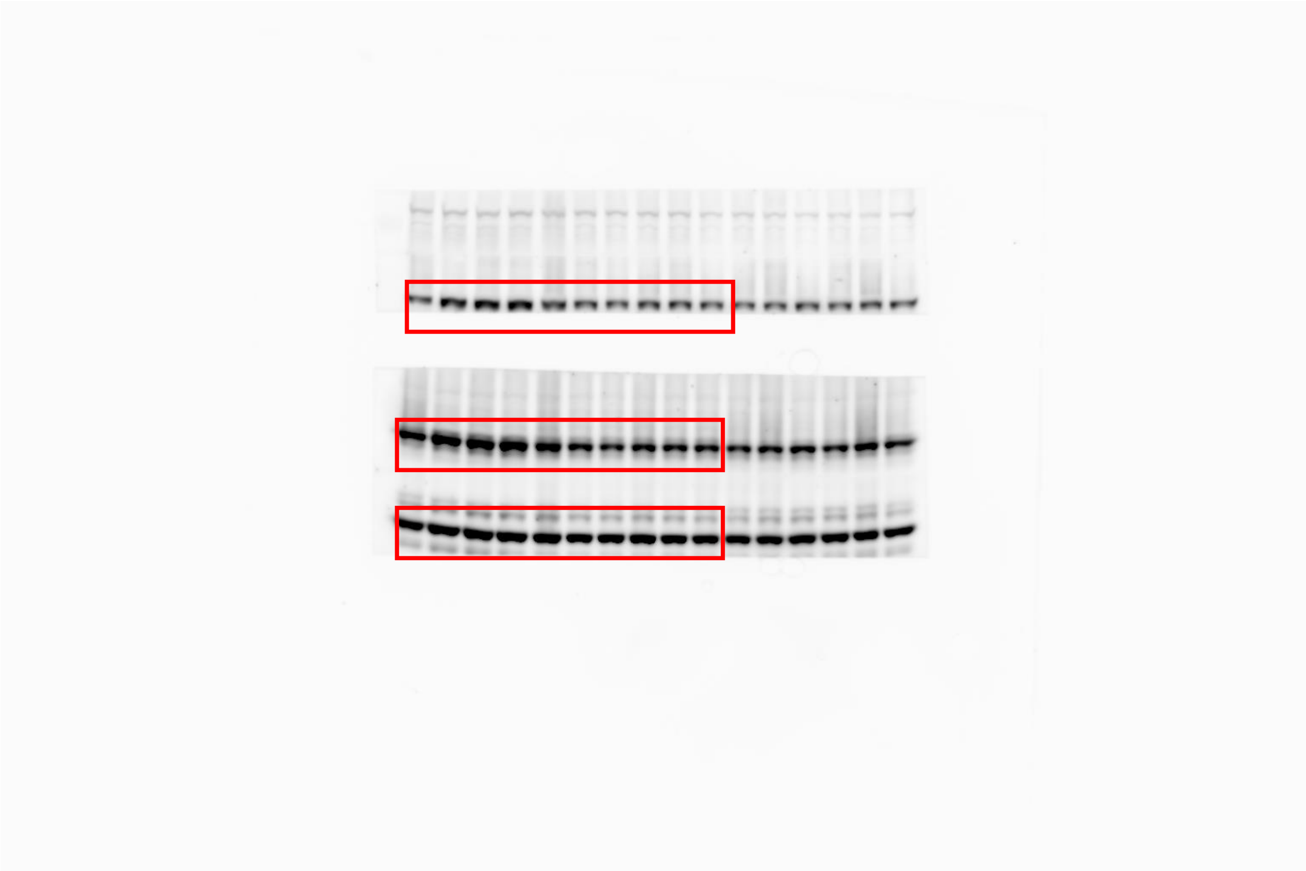

Supplementary Figure S6A. Talin-1 (270 kD), α-actinin (100), and GAPDH (36 kD)

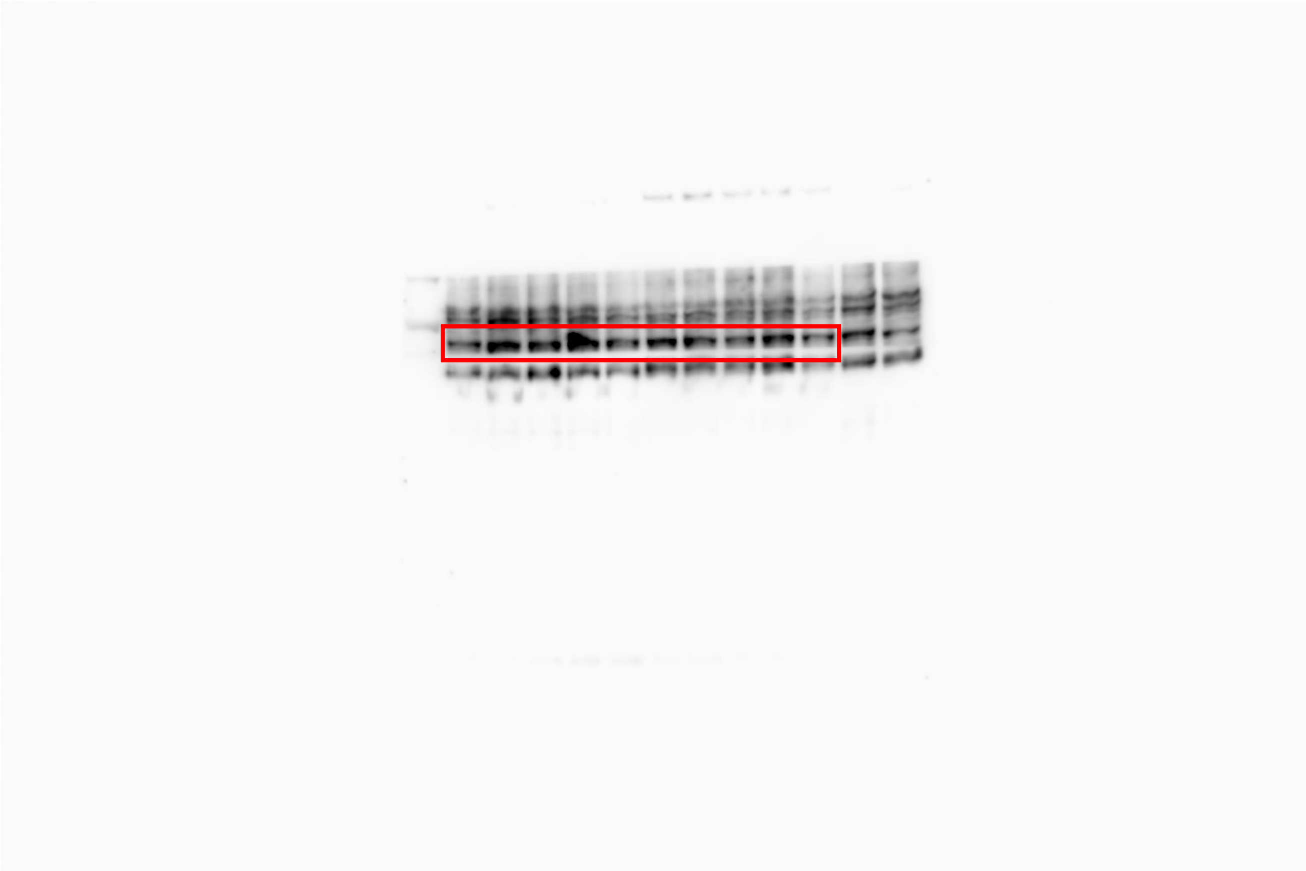

Supplementary Figure S6A. Vinculin (124 kD)

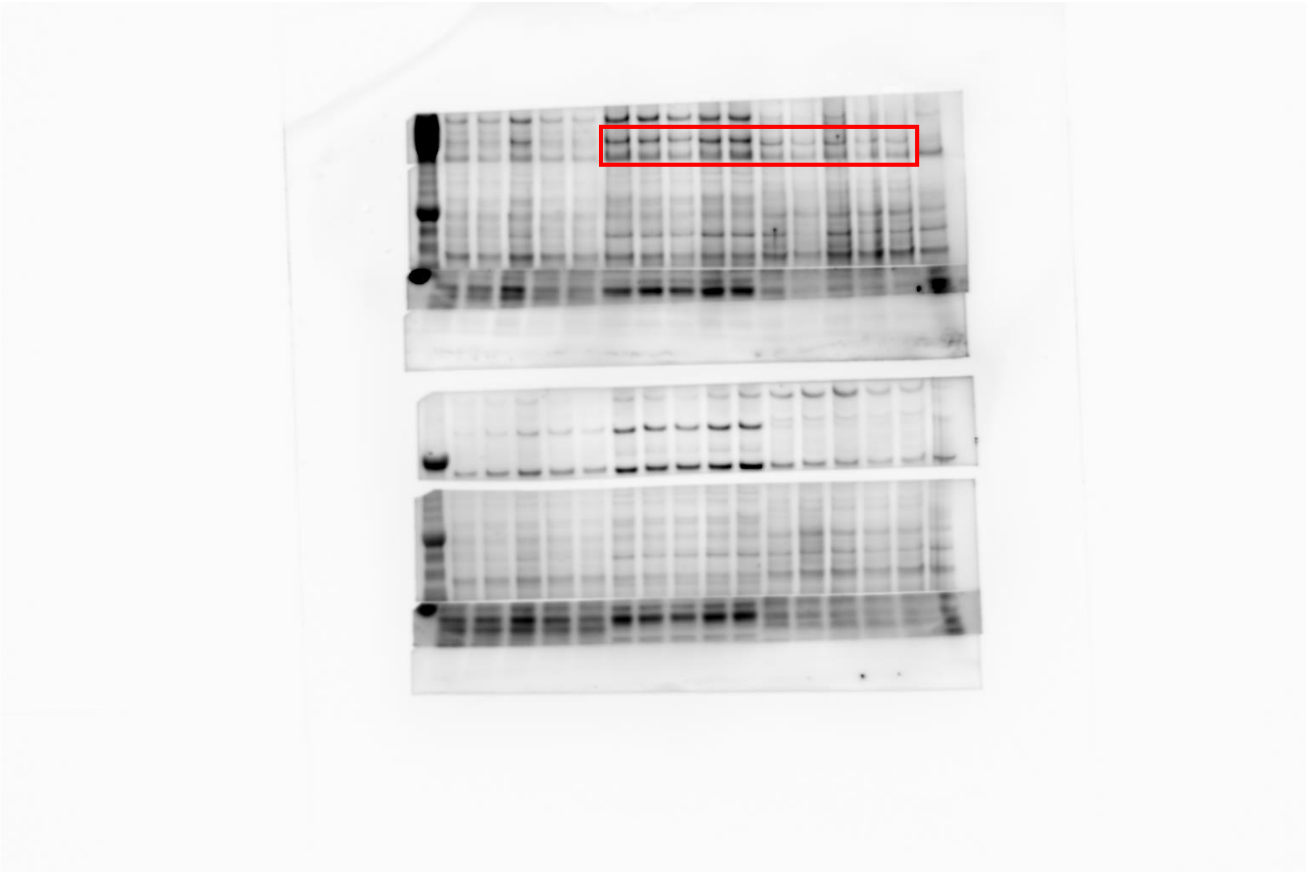

Supplementary Figure S6A. Cortactin (80,85 kD)

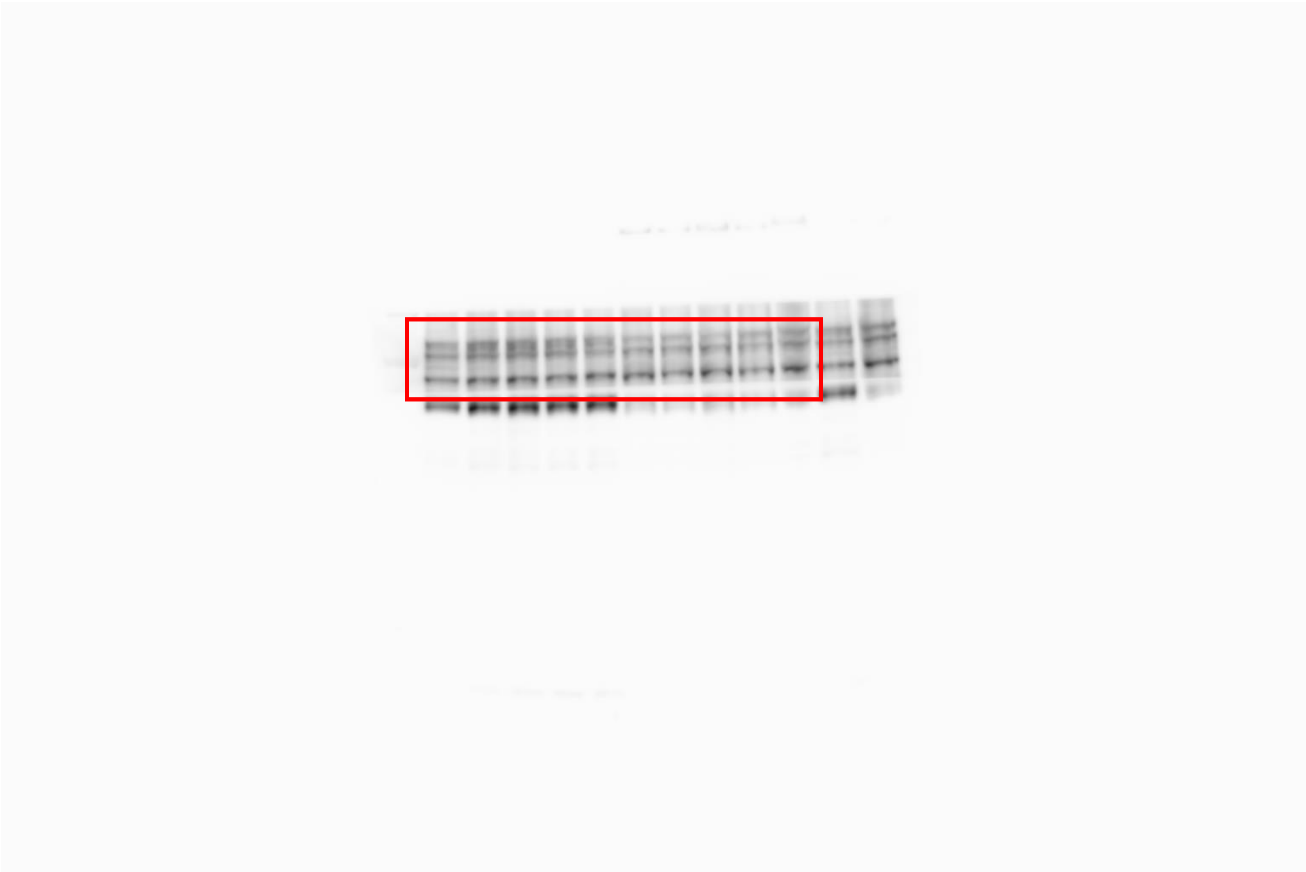

Supplementary Figure S6A. Paxillin (54,62,68 kD)

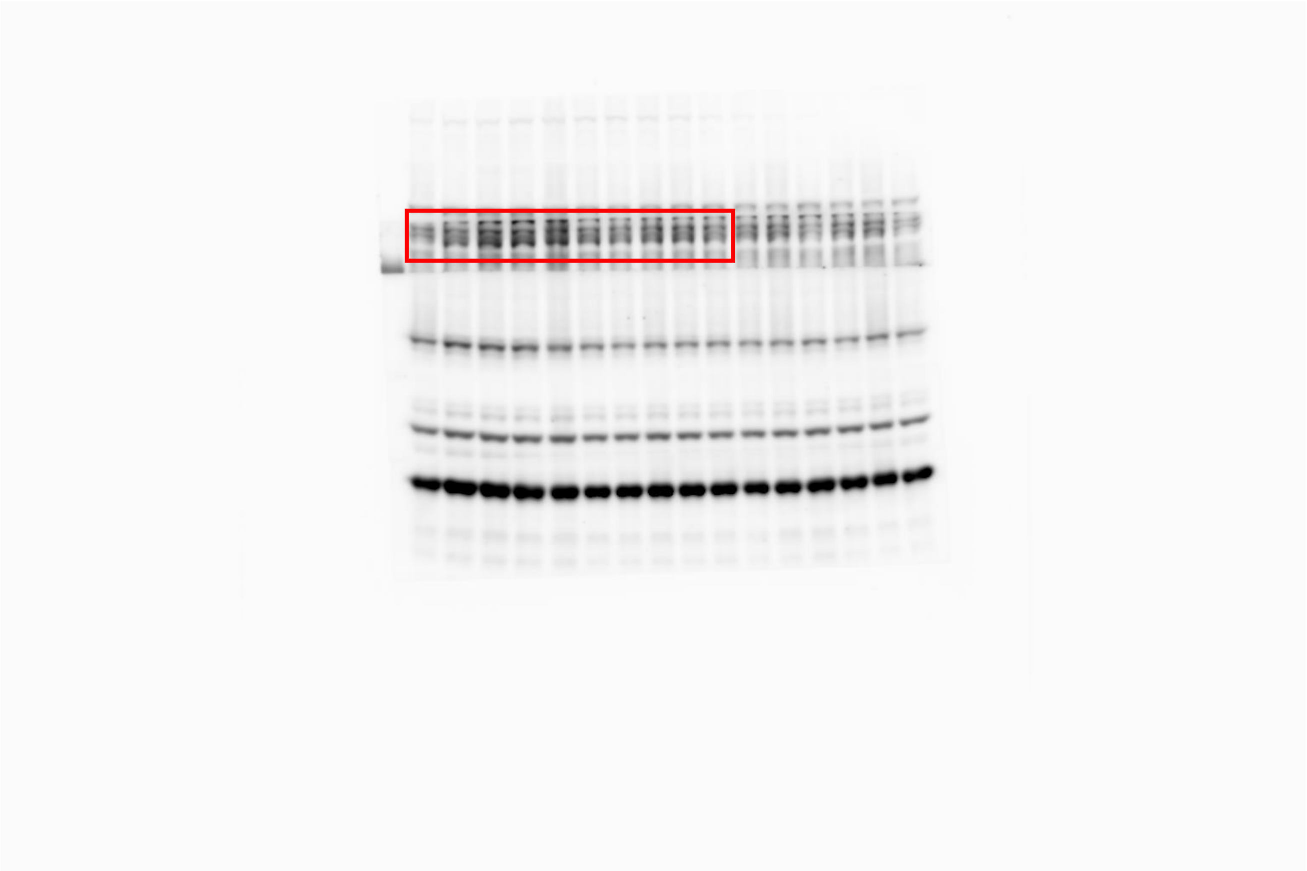

Supplementary Figure S6G. pFAK (125 kD)

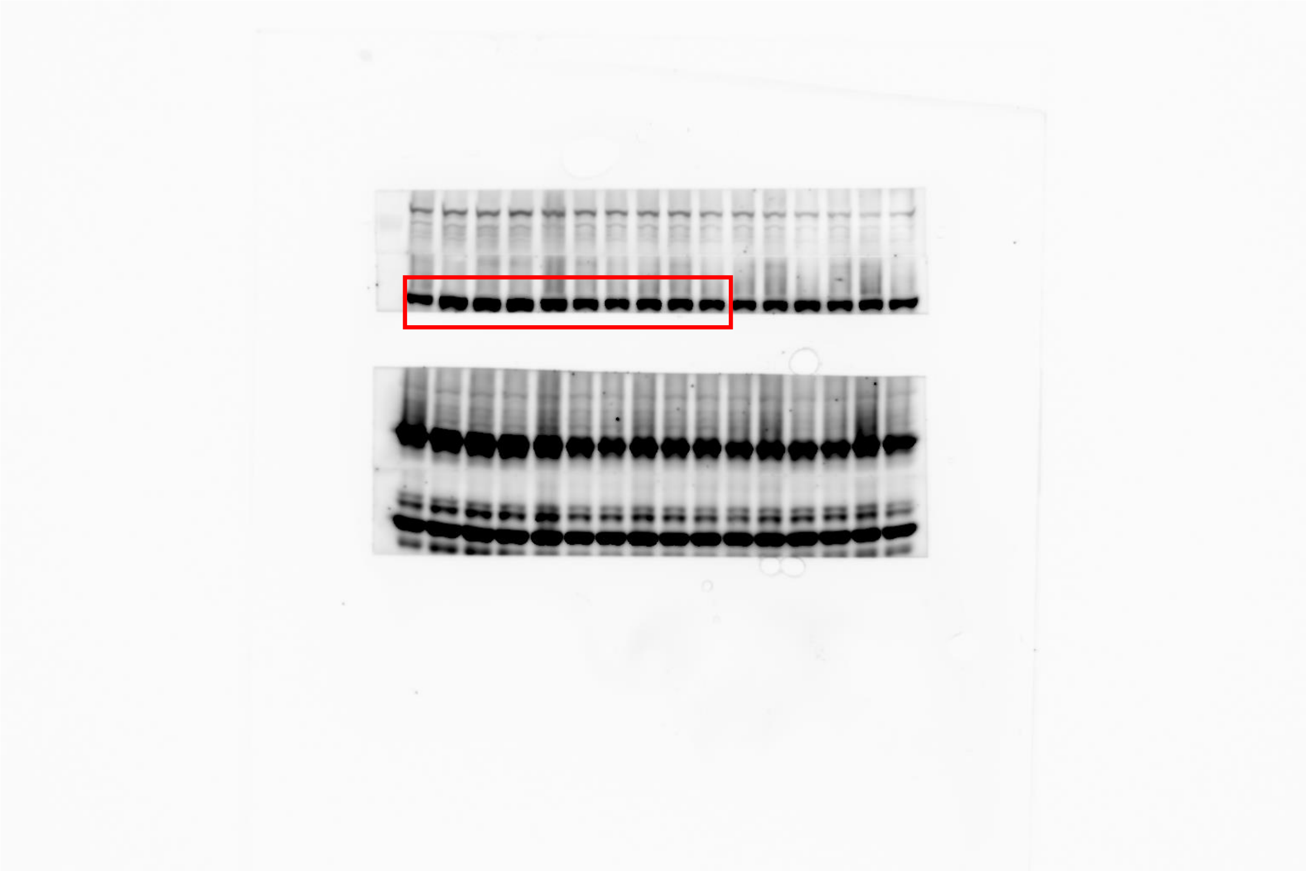

Supplementary Figure S6G. FAK (125 kD)

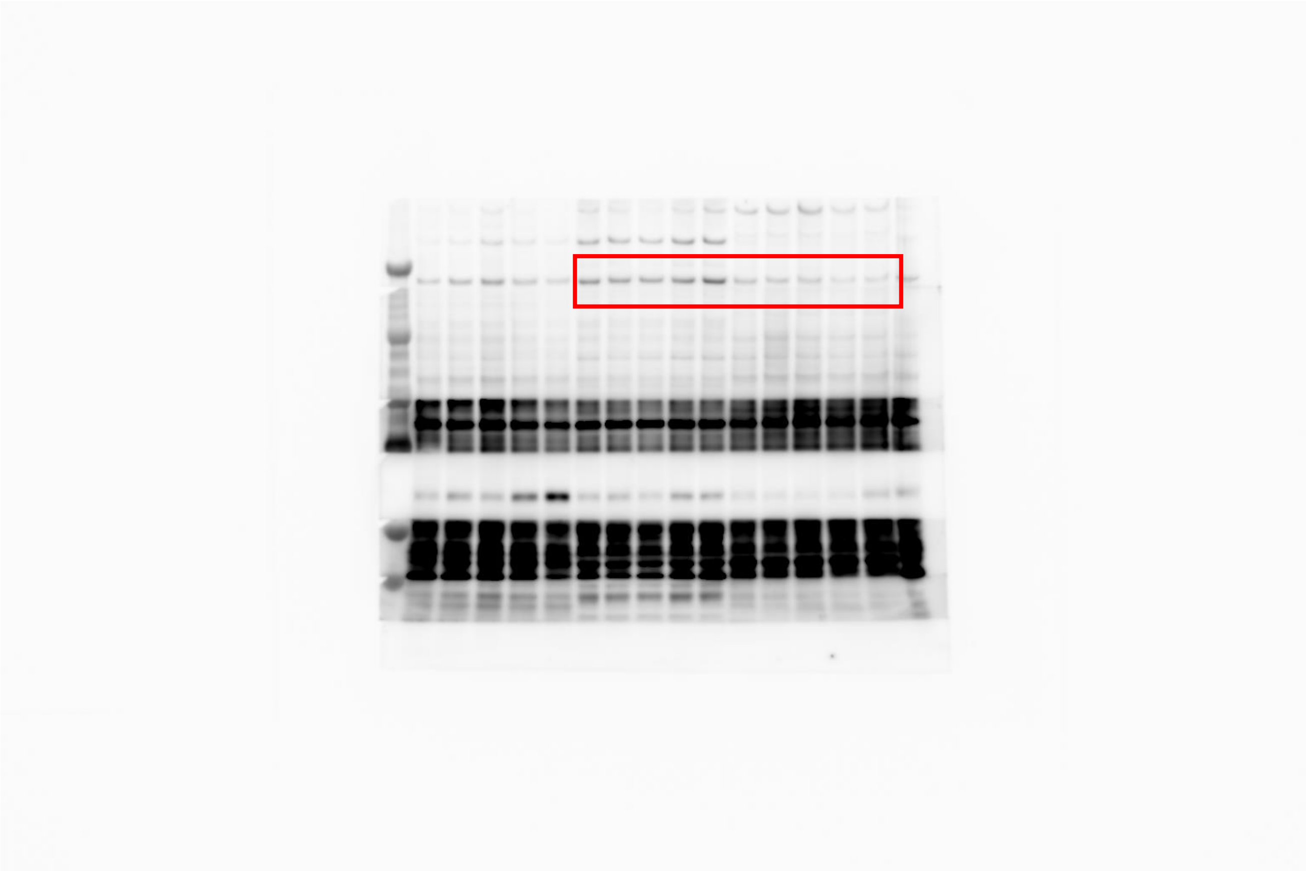

Supplementary Figure S6G. pFAK (125 kD)

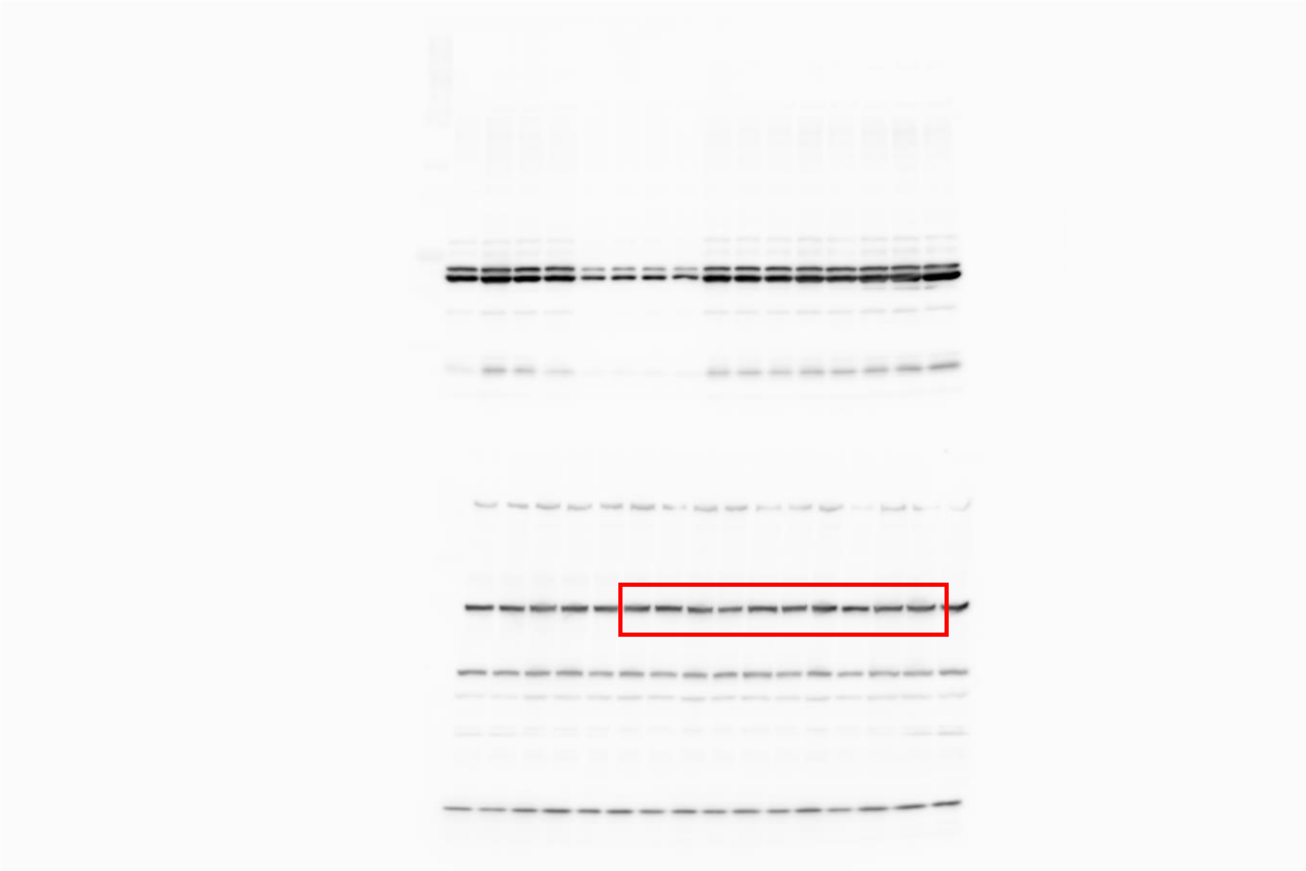

Supplementary Figure S6G. FAK (125 kD)

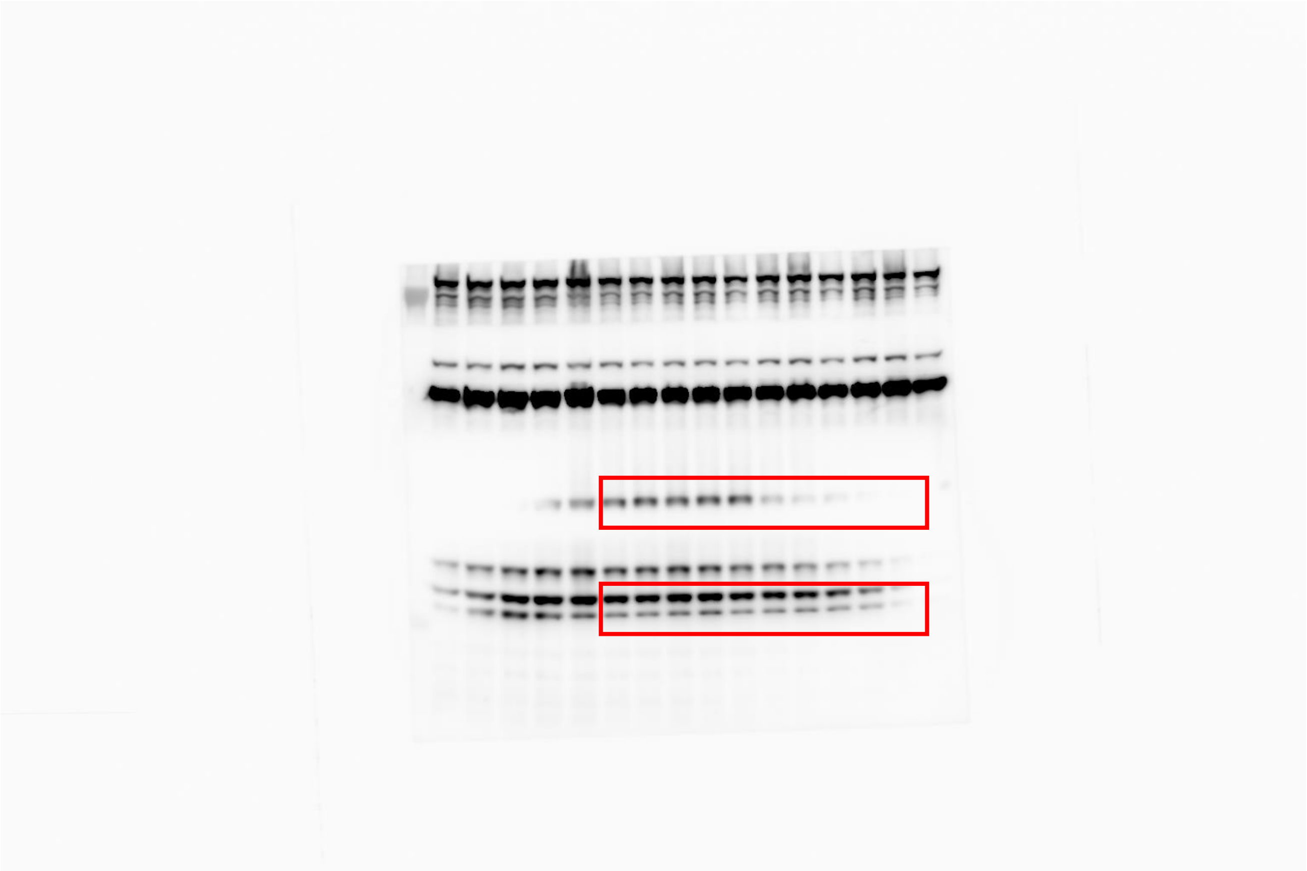

Supplementary Figure S6G. pAkt (60 kD), and pERK1/2 (42/44)

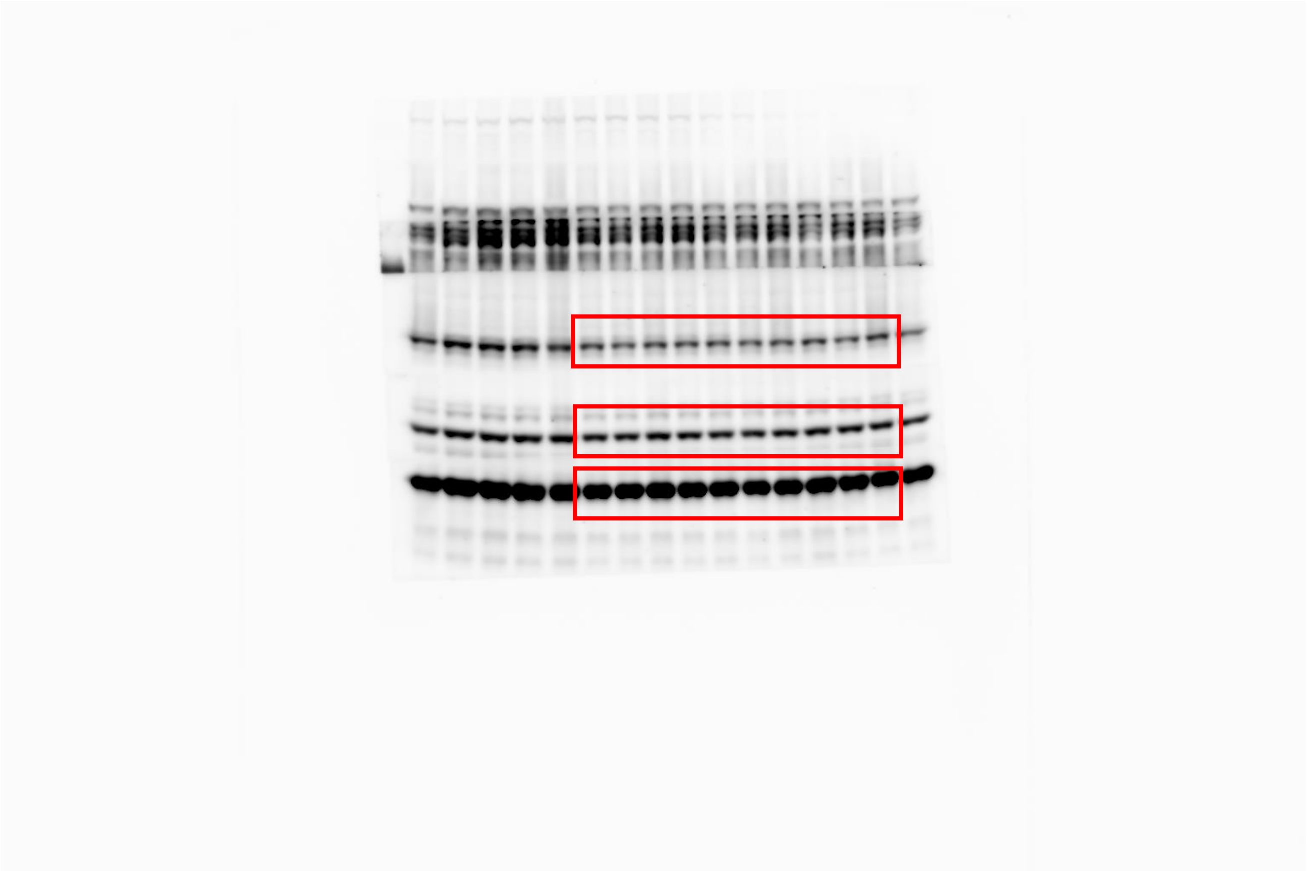

Supplementary Figure S6G. Akt (60 kD), ERK1/2 (42/44), and GAPDH (36 kD)

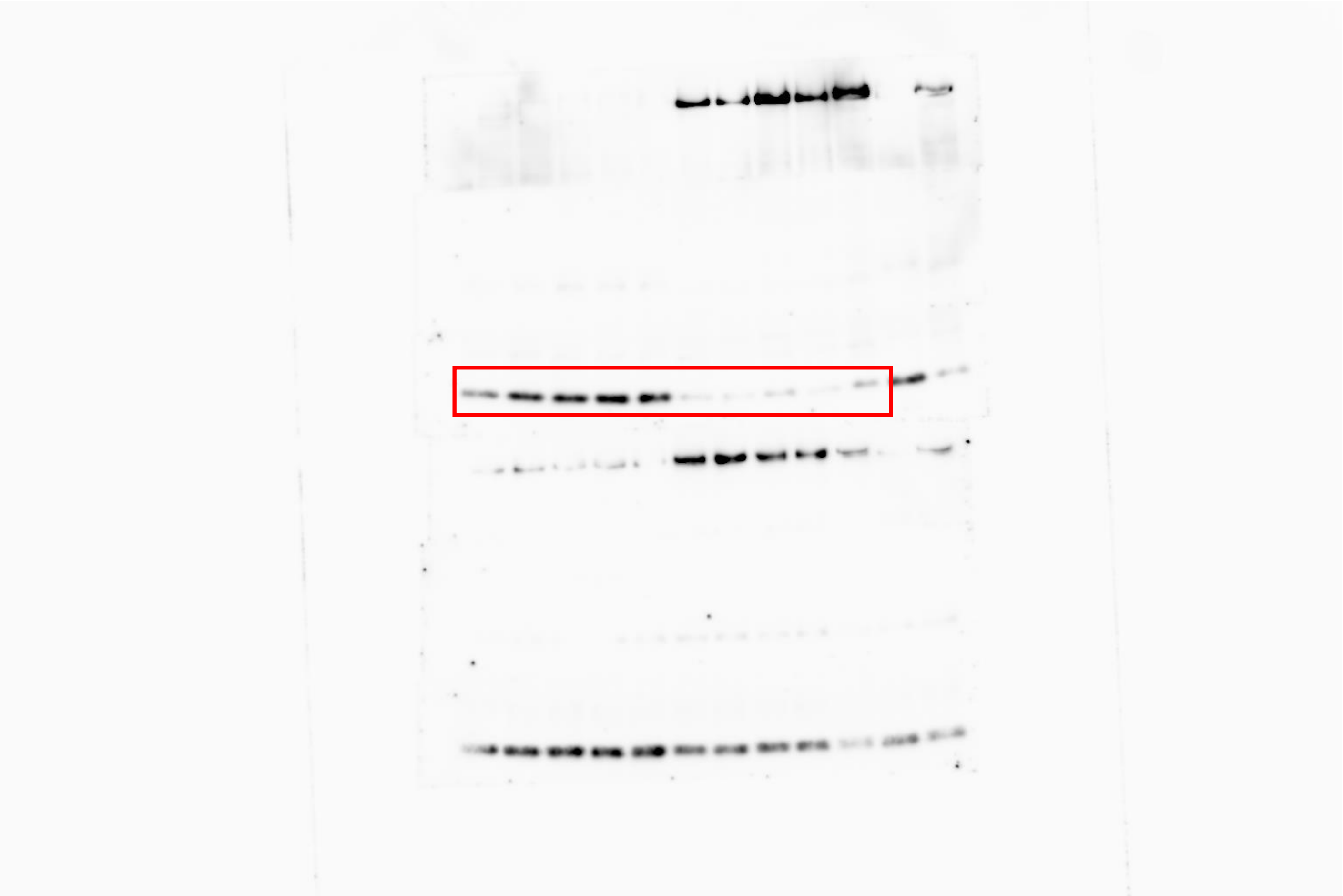

Supplementary Figure S6L. pRac/Cdc42 (28 kD)

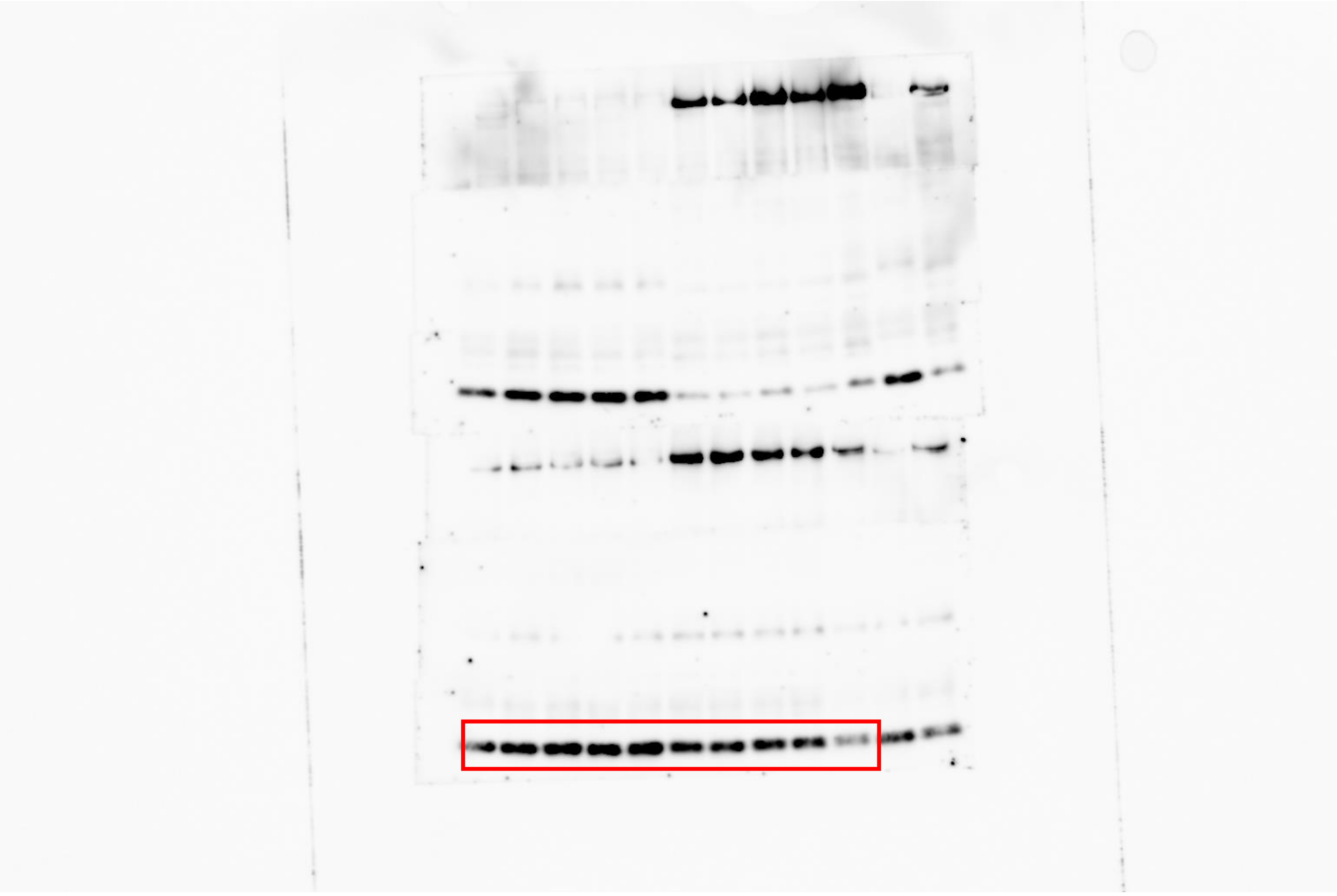

Supplementary Figure S6L. Rac/Cdc42 (28 kD)

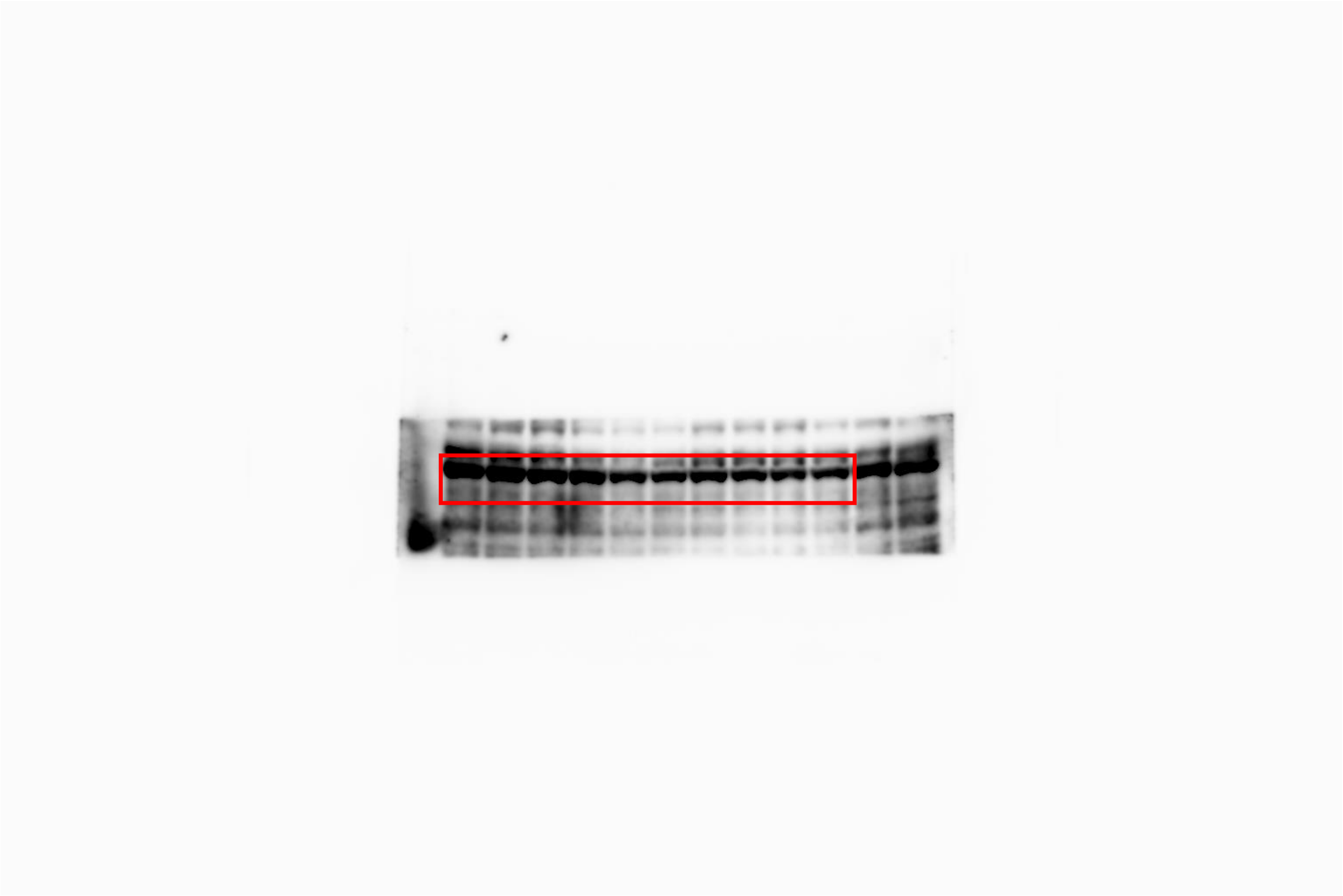

Supplementary Figure S6L. GAPDH (36 kD)

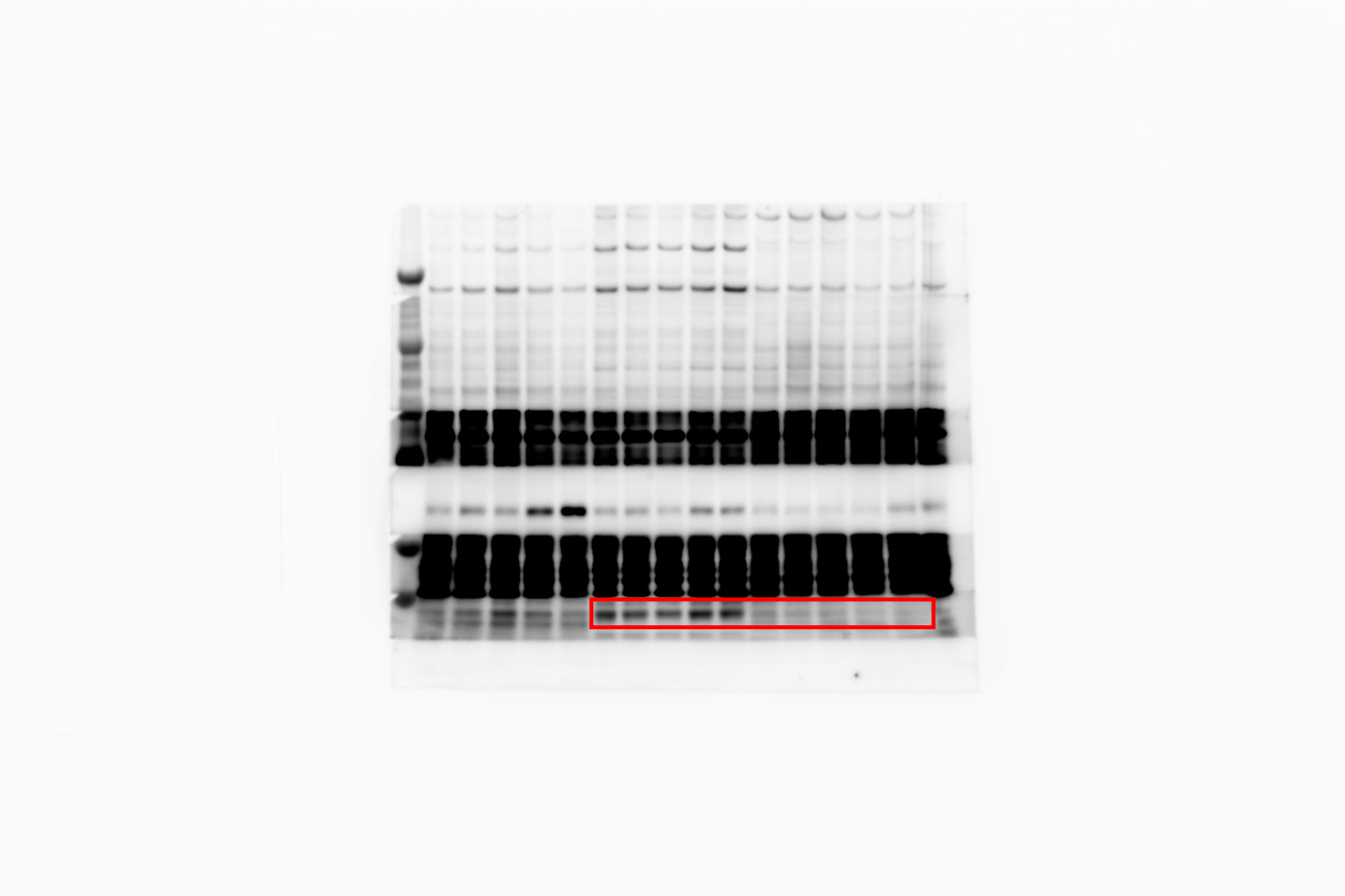

Supplementary Figure S6N. pRac/Cdc42 (28 kD)

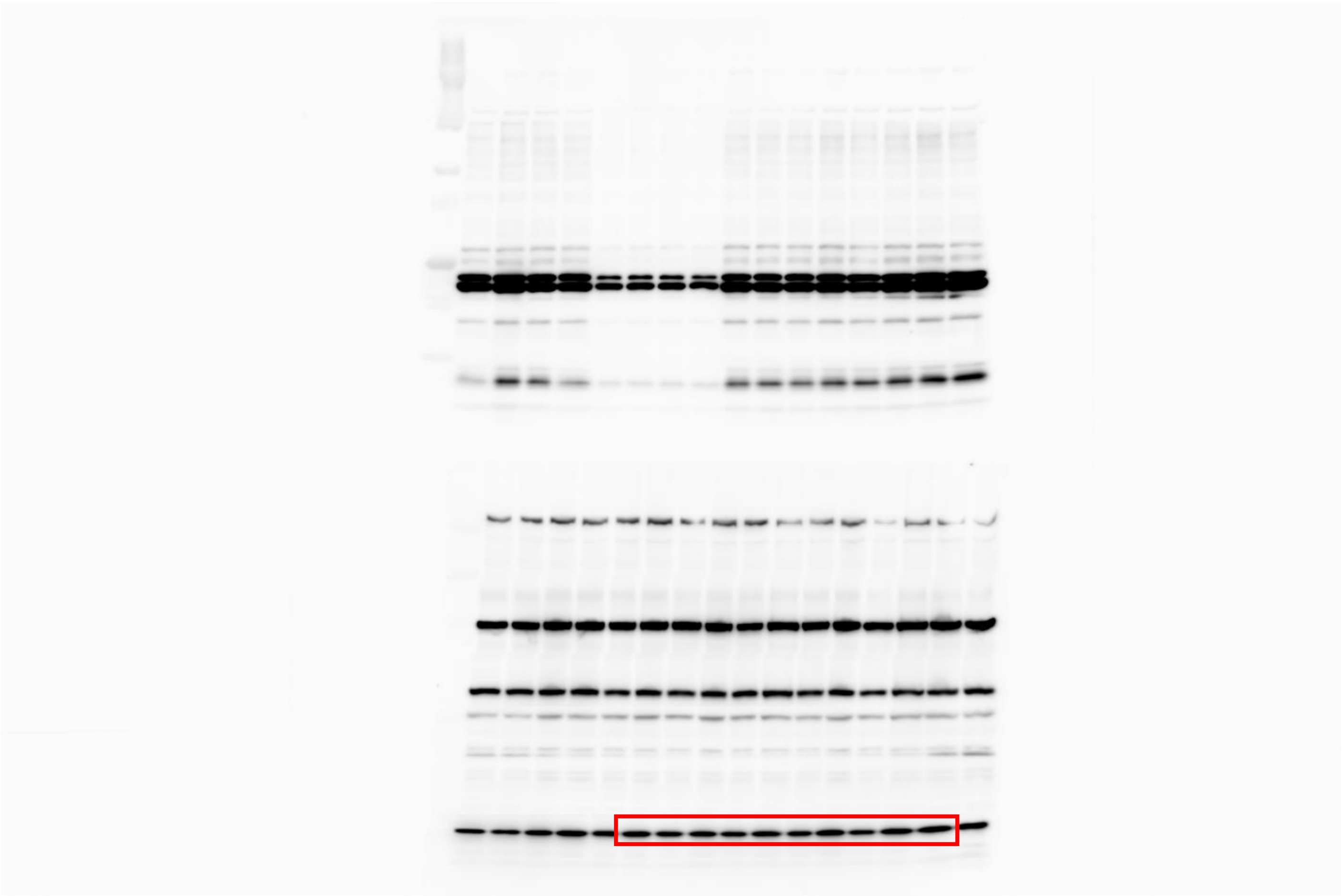

Supplementary Figure S6N. Rac/Cdc42 (28 kD)

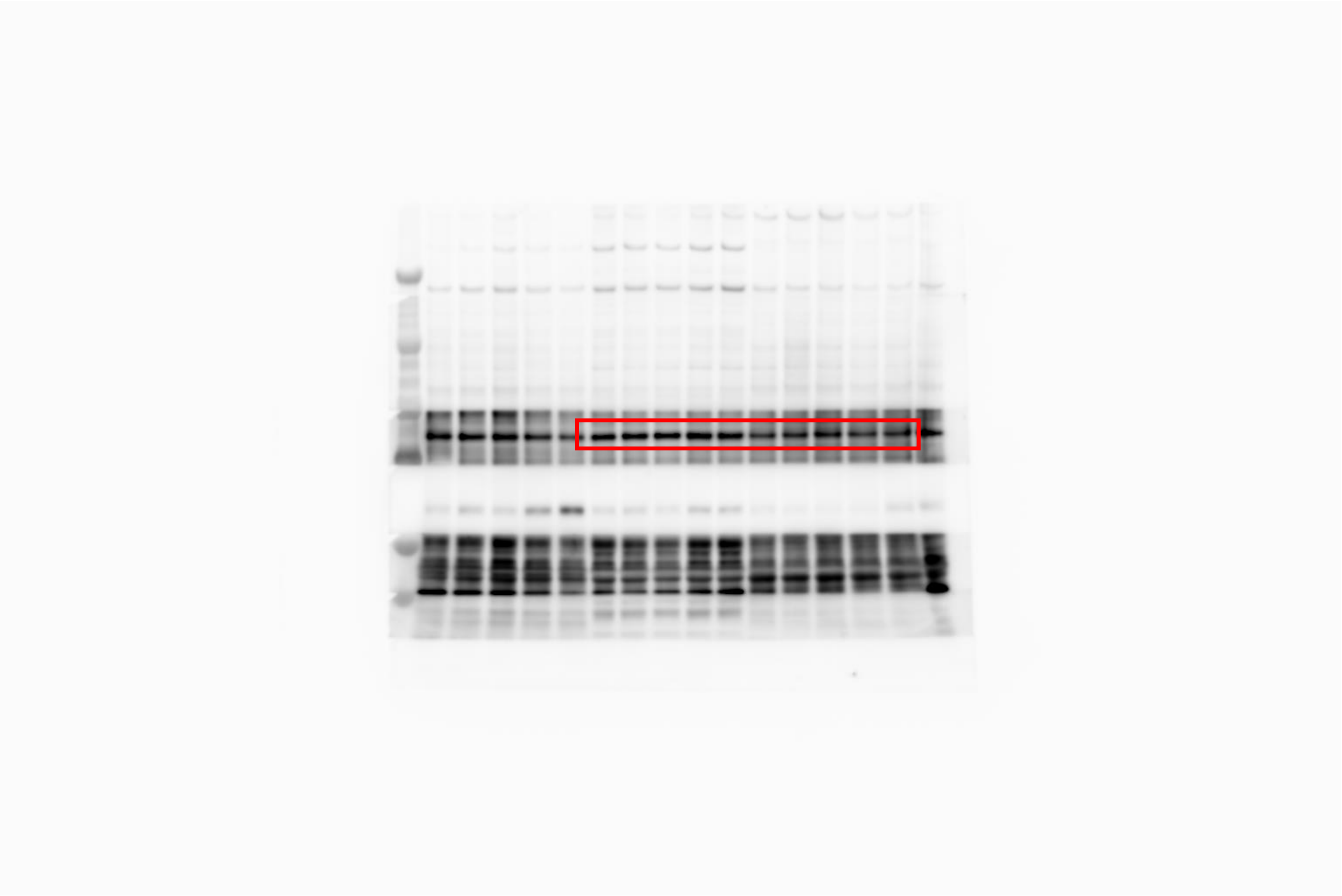

Supplementary Figure S6N. GAPDH (36 kD)
