# Supplementary material for: Gadolinium-based contrast agent accelerates the migration of astrocyte via integrin αvβ3 signaling pathway
Source: Sci Rep. 2022 Apr 7;12:5850. doi: 10.1038/s41598-022-09882-7 (PMC8990080; doi:10.1038/s41598-022-09882-7)
Supplement: Supplementary file 10 — Supplementary Information 10. [file 41598_2022_9882_MOESM10_ESM.pdf]

Supplementary Information

Gadolinium-based contrast agent accelerates the migration of astrocyte *via* integrin αvβ3 signaling pathway

Winda Ariyani[1,2*], Wataru Miyazaki[2,3], Yoshito Tsushima[4], Noriyuki Koibuchi[2*].

[1] Research Fellow of Japan Society for the Promotion of Science, 5-3-1 Kojimachi, Chiyoda-ku, Tokyo 102-0083, Japan, winda@gunma-u.ac.jp.

[2] Department of Integrative Physiology, Gunma University Graduate School of Medicine, 3-39-22 Showa-machi Maebashi, Gunma 371-8511, Japan, nkoibuch@gunma-u.ac.jp.

[3] Department of Bioscience and Laboratory Medicine, Hirosaki University Graduate School of Health Science, 66-1 Hon-cho Hirosaki, Aomori 036-8564 Japan, miya@hirosaki-u.ac.jp.

[4] Department of Diagnostic Radiology and Nuclear Medicine, Gunma University Graduate School of Medicine, 3-39-22 Showa-machi Maebashi, Gunma 371-8511, Japan, yoshitotsushima@gunma-u.ac.jp.

Running title: GBCA effects on astrocyte migration

*Correspondence authors:
Winda Ariyani, PhD.
Noriyuki Koibuchi, MD., PhD.
Department of Integrative Physiology, Gunma University, Graduate School of Medicine.
3-39-22 Showamachi Maebashi, Gunma 371-8511 Japan.
Phone No.: +81-27-220-7923
FAX No.: +81-27-220-7926
E-mail address: winda@gunma-u.ac.jp; nkoibuch@gunma-u.ac.jp

Supplementary Figure Legends

Supplementary Figure S1. GBCAs increased astrocyte migration in C6 and U87MG cells. Quantitative analysis of the effect of GBCAs (1–100 nM) on cell invasion was performed by matrigel invasion assay in C6 (A) and U87MG (B) cells. The total number of cells was quantified using ImageJ software (NIH). Data are expressed as the mean ± SEM of at least three independent experiments. $^{****}p$ <0.0001, $^{**}p$ <0.01, $^{*}p$ <0.05, indicates statistical significance were analyzed by ANOVA continue with post hoc Bonferroni's or Turkey test compared with the control. Both analysis showed the same statistical significances.

Supplementary Figure S2. (A) Quantitative analysis of the effects of Rhosin, CASIN, ML–141, and/or EHT–1864 on GBCAs-accelerated cell migration, measured using matrigel invasion assays using cortical astrocytes. (B) Quantitative analysis of the effects of Rhosin, CASIN, ML–141, and/or EHT–1864 on GBCAs-accelerated cell adhesion, measured using cell adhesion assays using cortical astrocytes. The total number of cells was quantified using ImageJ software (NIH). Data are expressed as the mean ± SEM of at least three independent experiments. $^{****}p$ <0.0001, indicates statistical significance were analysed by two-way ANOVA continue with post hoc Bonferroni's or Turkey test compared with the control. $^{\#\#\#\#}p$ <0.0001, $^{\#\#}p$ <0.01, $^{\#}p$ <0.05, indicates statistical significance were analysed by two-way ANOVA continue with post hoc Bonferroni's or Turkey. Both analysis showed the same statistical significances.

Supplementary Figure S3. Effects of GBCAs on integrin αvβ3, phosphorylation of ERK1/2 and Akt. Representative photomicrographs showing immunocytochemistry results for (A) integrin αvβ3 and (B) phosphorylated proteins of p-FAK, p-ERK1/2, and p-Akt with F-actin and DAPI

staining to examine the effects of GBCAs in U87MG cells. After fixation, cells were incubated with mouse anti-human integrin αvβ3 monoclonal antibody (1:200; Milipore, CA, USA), rabbit anti-phospho-ERK1/2 (T202/Y204) (1:200; Cell Signaling) antibody, or rabbit anti-phospho-Akt (S473) (1:200; Cell Signaling) antibody, followed by incubation with donkey anti-rabbit IgG (H+L) secondary antibody, Alexa Fluor 594 conjugate (1:200; Thermo Fisher Scientific) and CytoPainter Phalloidin-iFluor 488 reagent (AbCam). The cell nuclei were also stained with DAPI. Cells were then inspected under a laser confocal scanning microscope (ZEISS LSM 880, Carl Zeiss Microscopy GmbH, Jena, Germany). Bars represent 50 µm.

Supplementary Figure S4. Representative photomicrographs showing the effects Cyclo (Ala-Arg-Gly-Asp-3-Aminomethylbenzoyl) or cRGDfV on Omniscan–accelerated cell adhesion measured using cell adhesion assays. Bars represent 50 µm.

Supplementary Figure S5. GBCAs effect after siRNA transfection. The change in *ITGAV or ITGB3* mRNA expression levels in Astrocytes (A) and U87MG cells (B) after siRNA transfection. All experiments were repeated 3 times, using independent RNA preparations to confirm the consistency of the results. The mRNA levels were normalized by the mRNA level of GAPDH. Table 1. siRNA target sequences. Table 2. List of qRT-PCR primer sequences.

Supplementary Figure S6. Effects integrin inhibitor in GBCAs induced integrin signaling pathways. (A) Representative blots of talin-1, vinculin, α-actinin, cortactin, paxillin, and GAPDH levels of 60 min GBCAs exposure after co-exposure with 100 nM of cRGDfV in U87MG cells. Quantitative analysis of GBCAs effect on the protein expression levels of talin-1 (B), vinculin (C),

α-actinin (D), cortactin (E), and paxillin (F) after co-exposure with 100 nM cRGDfV. (G) Representative blots of pFAK, FAK, pAkt, Akt, pERK1/2 and ERK1/2 levels of GBCAs exposure after co-exposure with 100 nM of cRGDfV. Quantitative analysis of GBCAs effect on the protein expression levels of FAK (Y397) (H), FAK (Y925) (I), Akt (S473) (J), and ERK1/2 (T202/Y204) (K) after co-exposure with 100 nM of cRGDfV. Representative blots of pRac1/Cdc42 levels of GBCAs exposure after knockdown of ITGAV and ITGB3 (L) or coexposure with cRGDfV (N). Quantitative analysis of GBCAs effect on the protein expression levels of Rac1/Cdc42 (S71) after knockdown of ITGAV and ITGB3 (M) or co-exposure with cRGDfV (O). The blots were quantified using Fiji ImageJ software (NIH). Data are expressed as the mean ± SEM of at least three independent experiments. $^{****}p$ <0.0001, $^{***}p$ <0.001, $^{**}p$ <0.01, $^{*}p$ <0.05, indicates statistical significance were analyzed by two-way ANOVA continue with post hoc Bonferroni's or Turkey test compared with the control. $^{\#\#\#\#}p$ <0.0001, $^{\#\#\#}p$ <0.001, $^{\#\#}p$ <0.01, $^{\#}p$ <0.05, indicates statistical significance were analysed by two-way ANOVA continue with post hoc Bonferroni's or Turkey. Both analysis showed the same statistical significances.

Supplementary Figure S7. Effects of GBCAs or $GdCl_3$ exposure on the cellular viability. C6 cells were exposed to several concentrations of $GdCl_3$ (A), Omniscan (B), Magnescope (C), Magnevist (D), and Gadovist (E) for 24 h. Cell viability was determined by MTS cell proliferation assays. Data are presented as means ± SEM of experiments performed in triplicate. $^{****}p$ <0.0001, $^{***}p$ <0.001, $^{**}p$ <0.01, $^{*}p$ <0.05, indicates statistical significance were analyzed by two-way ANOVA continue with post hoc Bonferroni's or Turkey test compared with the control (no Gd treatment). Both analysis showed the same statistical significances.

Supplementary Figure S8. Effects of $GdCl_3$ exposure on the cell migration. Quantitative analysis of the effect of 10 nM $GdCl_3$ on (A) cell migration measured by matrigel invasion assay in astrocytes, (B) cell invasion measured by matrigel invasion assay in astrocytes, and (C) cell migration measured by wound healing assay in C6 cells. Data are presented as means ± SEM of experiments performed in triplicate. $^{****}p$ <0.0001, $^{***}p$ <0.001, $^{**}p$ <0.01, $^{*}p$ <0.05, indicates statistical significance were analysed by two-way ANOVA continue with post hoc Bonferroni's or Turkey. Both analysis showed the same statistical significances.

Supplementary Figure S9. Original images of full-length blots. The target protein is indicated in the red rectangle.
